# Supplementary figures and images for: Mapping QTLs underpin nutrition components in aromatic rice germplasm
Source: PLoS One. 2020 Jun 11;15(6):e0234395. doi: 10.1371/journal.pone.0234395 (PMC7289389; doi:10.1371/journal.pone.0234395)

Supplementary file 2

Figure S1: Genetic map, frequency distribution pattern of phenotypic data.


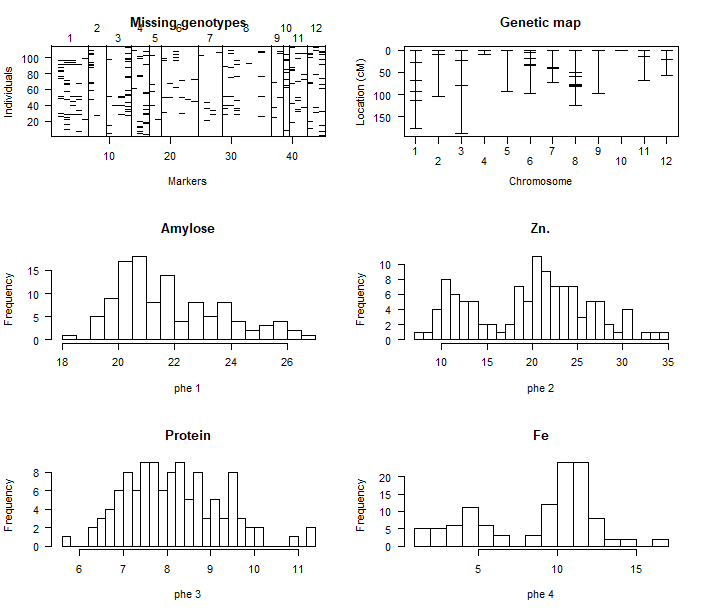

Supplement: S1 Fig — (DOCX) [file pone.0234395.s002.docx]
