# Supplementary material for: Mapping QTLs underpin nutrition components in aromatic rice germplasm
Source: PLoS One. 2020 Jun 11;15(6):e0234395. doi: 10.1371/journal.pone.0234395 (PMC7289389; doi:10.1371/journal.pone.0234395)
Supplement: S1 Table — (DOC) [file pone.0234395.s003.doc]

**Table S1. List of 52 SSR markers used in this study**

| **Name of marker** | **Chro.No.** | **Position (cM)** | **Product size (bp)** | **Forward primer sequence (5 to 3)** | **Reverse primer sequence (5 to 3)** |
| --- | --- | --- | --- | --- | --- |
| RM5 | 1 | 94.9 | 113 | TGCAACTTCTAGCTGCTCGA | GCATCCGATCTTGATGGG |
| RM495 | 1 | 2.8 | 159 | AATCCAAGGTGCAGAGATGG | CAACGATGACGAACACAACC |
| RM431 | 1 | 178.3 | 251 | TCCTGCGAACTGAAGAGTTG | AGAGCAAAACCCTGGTTCAC |
| RM237 | 1 | 115.2 | 130 | CAAATCCCGACTGCTGTCC | TGGGAAGAGAGCACTACAGC |
| RM312 | 1 | 71.6 | 97 | GTATGCATATTTGATAAGAG | AAGTCACCGAGTTTACCTTC |
| RM283 | 1 | 31.4 | 151 | GTCTACATGTACCCTTGTTGGG | CGGCATGAGAGTCTGTGATG |
| RM452 | 2 | 58.4 | 209 | CTGATCGAGAGCGTTAAGGG | GGGATCAAACCACGTTTCTG |
| RM6 | 2 | 154.7 | 163 | GTCCCCTCCACCCAATTC | TCGTCTACTGTTGGCTGCAC Bottom of Form |
| RM322 | 2 | 49.7 | 112 | CAAGCGAAAATCCCAGCAG | GATGAAACTGGCATTGCCTG |
| RM489 | 3 | 29.2 | 271 | ACTTGAGACGATCGGACACC | TCACCCATGGATGTTGTCAG |
| RM338 | 3 | 108.4 | 183 | CACAGGAGCAGGAGAAGAGC | GGCAAACCGATCACTCAGTC |
| OSR13 | 3 | 53.1 | 0 | CATTTGTGCGTCACGGAGTA | AGCCACAGCGCCCATCTCTC |
| RM514 | 3 | 216.4 | 259 | AGATTGATCTCCCATTCCCC | CACGAGCATATTACTAGTGG |
| RM307 | 4 | 0 | 174 | GTACTACCGACCTACCGTTCAC | CTGCTATGCATGAACTGCTC |
| RM537 | 4 | 8.5 | 236 | CCGTCCCTCTCTCTCCTTTC | ACAGGGAAACCATCCTCCTC |
| RM551 | 4 | 8.5 | 192 | AGCCCAGACTAGCATGATTG | GAAGGCGAGAAGGATCACAG |
| RM178 | 5 | 118.8 | 117 | TCGCGTGAAAGATAAGCGGCGC | GATCACCGTTCCCTCCGCCTGC |
| RM413 | 5 | 26.7 | 79 | GGCGATTCTTGGATGAAGAG | TCCCCACCAATCTTGTCTTC |
| RM510 | 6 | 20.8 | 122 | AACCGGATTAGTTTCTCGCC | TGAGGACGACGAGCAGATTC |
| RM454 | 6 | 99.3 | 268 | CTCAAGCTTAGCTGCTGCTG | GTGATCAGTGCACCATAGCG |
| RM170 | 6 | 2.2-7.4 | 121 | TCGCGCTTCTTCCTCGTCGACG | CCCGCTTGCAGAGGAAGCAGCC |
| RM190 | 6 | 7.4 | 124 | GCATTGTCATGTCGAAGCC | CTAGCAGGAACTCCTTTCAGG |
| RM253 | 6 | 37 | 141 | TCCTTCAAGAGTGCAAAACC | GCATTGTCATGTCGAAGCC |
| RM314 | 6 | 33.6 | 118 | CTAGCAGGAACTCCTTTCAGG | AACATTCCACACACACACGC |
| RM455 | 7 | 65.7 | 131 | AACAACCCACCACCTGTCTC | AGAAGGAAAAGGGCTCGATC |
| RM118 | 7 | 96.9 | 156 | CCAATCGGAGCCACCGGAGAGC | CACATCCTCCAGCGACGCCGAG |
| RM125 | 7 | 24.8 | 146 | ATCAGCAGCCATGGCAGCGACC | AGGGGATCATGTGCCGAAGGCC |
| RM10 | 7 | 63.5 | 159 | TTGTCAAGAGGAGGCATCG | CAGAATGGGAAATGGGTCC |
| RM408 | 8 | 0-1.1 | 156 | CAACGAGCTAACTTCCGTCC | ACTGCTACTTGGGTAGCTGACC |
| RM25 | 8 | 52.2 | 146 | GGAAAGAATGATCTTTTCATGG | CTACCATCAAAACCAATGTTC |
| RM44 | 8 | 60.9 | 99 | ACGGGCAATCCGAACAACC | TCGGGAAAACCTACCCTACC |
| RM284 | 8 | 83.7 | 141 | TCCTTGTGAAATCTGGTCCC | GTAGCCTAGCATGGTGCATG |
| RM447 | 8 | 124.6 | 111 | CCCTTGTGCTGTCTCCTCTC | ACGGGCTTCTTCTCCTTCTC |
| RM223 | 8 | 80.5 | 165 | GAGTGAGCTTGGGCTGAAAC | GAAGGCAAGTCTTGGCACTG |
| RM342 | 8 | 78.4 | 141 | CCATCCTCCTACTTCAATGAAG | ACTATGCAGTGGTGTCACCC |
| RM515 | 8 | 80.5 | 211 | TAGGACGACCAAAGGGTGAG | TGGCCTGCTCTCTCTCTCTC |
| RM316 | 9 | 1.8 | 192 | CTAGTTGGGCATACGATGGC | ACGCTTATATGTTACGTCAAC |
| RM215 | 9 | 99.4 | 148 | CAAAATGGAGCAGCAAGAGC | TGAGCACCTCCTTCTCTGTAG |
| RM271 | 10 | 59.4 | 101 | TCAGATCTACAATTCCATCC | TCGGTGAGACCTAGAGAGCC |
| RM287 | 11 | 68.6 | 118 | TTCCCTGTTAAGAGAGAAATC | GTGTATTTGGTGAAAGCAAC |
| RM536 | 11 | 55.1 | 243 | TCTCTCCTCTTGTTTGGCTC | ACACACCAACACGACCACAC |
| RM144 | 11 | 123.2 | 237 | TGCCCTGGCGCAAATTTGATCC | GCTAGAGGAGATCAGATGGTAGTGCATG |
| RM19 | 12 | 20.9 | 226 | CAAAAACAGAGCAGATGAC | CTCAAGATGGACGCCAAGA |
| RM20 | 12 | 0 | 144 | ATCTTGTCCCTGCAGGTCAT | GAAACAGAGGCACATTTCATTG |
| RM277 | 12 | 57.2 | 124 | CGGTCAAATCATCACCTGAC | CAAGGCTTGCAAGGGAAG |

Monomorphic markers

| RM124 | 4 | 150.1 | 271 | ATCGTCTGCGTTGCGGCTGCTG | CATGGATCACCGAGCTCCCCCC |
| --- | --- | --- | --- | --- | --- |
| RM507 | 5 | 0 | 258 | CTTAAGCTCCAGCCGAAATG | CTCACCCTCATCATCGCC |
| RM433 | 8 | 116.0 | 224 | TGCGCTGAACTAAACACAGC | AGACAAACCTGGCCATTCAC |
| RM105 | 9 | 32.1 | 134 | GTCGTCGACCCATCGGAGCCAC | TGGTCGAGGTGGGGATCGGGTC |
| RM474 | 10 | 0 | 252 | AAGATGTACGGGTGGCATTC | TATGAGCTGGTGAGCAATG |
| RM484 | 10 | 97.3 | 299 | TCTCCCTCCTCACCATTGTC | TGCTGCCCTCTCTCTCTCTC |
| RM552 | 11 | 40.6 | 195 | CGCAGTTGTGGATTTCAGTG | TGCTCAACGTTTGACTGTCC |
