# Supplementary material for: Mapping QTLs underpin nutrition components in aromatic rice germplasm
Source: PLoS One. 2020 Jun 11;15(6):e0234395. doi: 10.1371/journal.pone.0234395 (PMC7289389; doi:10.1371/journal.pone.0234395)
Supplement: Attachment — Submitted filename: PONE-D-20-01600_R1.pdf [file pone.0234395.s005.pdf]

PLOS ONE

The assessment of epigenetic diversity, differentiation, and structure in the ‘Fuji’ mutation line implicates roles of epigenetic modification in the occurrence of different mutant groups as well as spontaneous mutants

--Manuscript Draft--

**Manuscript Number:**	PONE-D-20-01600R1
**Article Type:**	Research Article
**Full Title:**	The assessment of epigenetic diversity, differentiation, and structure in the ‘Fuji’ mutation line implicates roles of epigenetic modification in the occurrence of different mutant groups as well as spontaneous mutants
**Short Title:**	The epigenetic assessment of Fuji mutation line implicates roles of epigenetic modification
**Corresponding Author:**	Laiqing Song<br>Yantai Academy of Agricultural Sciences<br>Yantai, Shandong Province CHINA
**Keywords:**	Molecular marker; AFLP; MSAP; Apple; Bud sports; Genetic variation; Malus domestica Borkh.; genetic relationship
**Abstract:**	The Fuji line includes many varieties with a similar genetic background and consistent inducement factors with epigenetic occurrence, thus it may be considered an ideal candidate for epigenetic research. In this study, 91 bud mutations of ‘Fuji’ apple were used as the test materials. Using the genetic variation within ‘Fuji’ as the control, the characteristics of epigenetic variation at different levels in both varieties and mutant groups were examined. The results showed that: (1) the global genomic DNA methylation level of the 91 bud mutants of ‘Fuji’ ranged from 29.120%-45.084%, with an average of 35.910%. Internal cytosine methylation was the main DNA methylation pattern. Regarding the variation of methylation patterns of ‘Fuji’ mutants, the vast majority of loci maintained the original methylation pattern existed in ‘Fuji’. CHG methylation variation was the main type of variation. (2) The variation in methylation patterns between the mutant groups was greater than that of methylation levels. Among these patterns, the variation in CHG methylation patterns (including CHG hypermethylation and CHG demethylation) was expected to be dominant. The observed variation in methylation levels was more important in the Color mutant group; however, the variation in methylation patterns was more obvious in both the early maturation and Spur mutant groups. Moreover, the range of variation in the Early-maturation group was much wider than that in the Spur mutant group. (3) Epigenetic diversity and genetic diversity were both low between the mutant groups. In the ‘Fuji’ mutant groups, there was few correlation between genetic and epigenetic variation, and epigenetic differentiation resulted in more loci with moderate or greater differentiation. (4) Purifying selection seemed to play a major role in the differentiation of different groups of ‘Fuji’ mutants (65.618%), but epigenetic diversity selection still occurred at nearly 35% of loci. Sixteen epigenetic outlier loci were detected.
**Order of Authors:**	Xiaoyun Du
	Yanbo Wang
	Minxiao Liu
	Xueqing Liu
	Zhongwu Jiang
	Lingling Zhao
	Yan Tang
	Yanxia Sun
	Xueyong Zhang
	Daliang Liu

Powered by Editorial Manager® and ProduXion Manager® from Aries Systems Corporation

	Laiqing Song
**Opposed Reviewers:**	
**Response to Reviewers:**	To Reviewer1:<br>Thank you for your careful revision of our manuscript!<br>The followings are our Response for Comment, please check. Thanks a lot!<br>■ -Text is wordy and language not well structured. English should be improved throughout.<br>A:The section of the Introduction and Discussion have been rewritten. Also, we asked AJE: English Editing & Author Services for Research Publication as well as our friends oversea to help do correction in the revised manuscript. The certificate from AJE company will be attached in this manuscript. We are really sorry for the troubles caused to you because of our poor English.<br>■ line 91-94. Not clear. Score ‘1’ we also get by digestion by either HpaII, MspI enzymes due to no methylation (band present in both).<br>A:This is done in order to distinguish between MSLs (methylation-susceptible) and NMLs (nonmethylated) data in the MSAP amplification results since only the MSP data are meaningful. We use the R package msap for relevant statistics, not manual score. Therefore, in the revised manuscript, avoiding wordy, the explanation of this part was removed in the introduction section. Actually, the ‘1’ here refers to two situations that a certain sample is cut by HpaII and MspI. For situation of H0M1 or H0M1, when the band is scored to make MSLs matrix, both will be recorded ‘1’.<br>■Is not clear if Fuji mutants are considered varieties or genotypes.<br>A:Fuji mutants are considered varieties. We have unified all varieties in the new version manuscript.<br>■It is not clear how many individual plants were used from each of the 91 elite varieties<br>A:We used pooled individuals (ca. 5 plants being clonally propagated from a single mother variety) of each variety for analysis. (see line 141 in the new version manuscript)<br>■It is not clear how many technical repeats were used in the AFLP and MSAP experiments<br>A: The repeatability of banding patterns assessed by conducting two sets of independent MSAP and AFLP analyses and only the consistent bands were included. (see line 159-160 in the new version manuscript)<br>■line 157. How were leaves stored?<br>A: Young leaves immediately frozen in liquid nitrogen and then stored at -80℃ prior to DNA isolation. (see line 142 in the new version manuscript)<br>■lines 157-158. Which were these developmental and phenological stages? Perhaps authors could be more specific.<br>A: We sampled plant materials with the same age on the same dates (12 June 2016) when they were bearing fully expanded leaves. (see line 138 in the new version manuscript)<br><br>To Reviewer2:<br>Thank you for your careful revision of our manuscript!<br>The followings are our Response for Comment, please check. Thanks a lot!<br>■it seems that more than one author wrote the introduction. English must be improved in several parts (especially the first half).<br>A: The section of the Introduction and Discussion have been rewritten. Also, we asked AJE: English Editing & Author Services for Research Publication as well as our friends oversea to help do correction in the revised manuscript. The certificate from AJE company will be attached in this manuscript. We are really sorry for the troubles caused to you because of our poor English.<br>■line85. more details about these isozymes<br>A: Done. (See line 86 in the new version manuscript)<br>■Several small corrections are included in the attached file.<br>A: All done in the new version manuscript.<br>■line156. How many samples per variety?<br>A: We used pooled individuals (ca. 5 plants being clonally propagated from a single mother variety) of each variety for analysis (see line 141 in the new version manuscript). One sample was used per variety in this study.<br>■line171 and 177. Its better to be presented in a table as a supplementary data<br>A: Done.<br>■line201. this table goes to results section

Powered by Editorial Manager® and ProduXion Manager® from Aries Systems Corporation

	A: Done.<br>■ Statistics must be clearly improved. It should be clear to the readers which results are statistically significant and which are not.<br>A: In the new revised version, we have tried our best doing complementary in statistically significant analysis for items that could be conducted for significant analysis. But we are really sorry. Because we used one mixed sample per variety in our study, for some items, like CVs between different mutant groups, it was impossible to perform significance analysis.<br>■line278. what statistical analysis have you conducted to say that??<br>A: In the new revised draft, we performed a statistical analysis of the significant difference between CG and CHG methylation levels.<br>■line291. In how many cases do they occur these changes in the patterns of cytosine methylation?<br>A: We have tabulated the statistical results in the supplementary materials section. (See new added S5 Table)<br>■line303. 91 varieties???<br>A: Yes. It should be 91 varieties.<br>■line305. this paragraph better fits to the discussion section.line313. also this paragraph better fits to the discussion section.<br>A: The relevant content in these two paragraphs has been simplified and doing statement in the discussion section.<br>■Line447. not a clear seperation between the different groups<br>A: Yes. Because this is the clustering result based on AFLP data. The results showed that, unlike the epigenetic results, there was not a clear separation between the different groups genetically. They just presented local clustering and mixed phenomena.<br>■Line452. tested you have to be consistent using either genotypes, samples or varieties<br>A: Thank for your advice very much. We have changed all of them to variety as a unified statement.<br>■The discussion should be more focused on results. It's not clear If the epigenetic differences are significantly different between the three groups.<br>A: We adjusted the content in the discussion section to make our discussion more focus on results. Moreover, we did difference significance analysis of epigenetic differences between the three groups. The result was significantly different between the three groups.<br>■The discussion is very big and some points are very assumptive. it should be shortened and provide a clear conclusion which correlates directly with the results. A: According to your advice, discussion was shorted. Additionally, we also added conclusions in the discussion section.<br>■line667. Can you provide any literature?<br>A: Done. Please see literature 39-56.<br>■line716. is it statistically higher? please verify.<br>A: Yes. We performed a statistical analysis of the significant difference between CG and CHG methylation levels. The result exhibited significant difference ($P<0.01$).<br>■line720-729. Is there any literature supporting these results too?<br>A: Because of the adjustment in content, this part of the analysis was removed in the new version manuscript.<br>■line733. moderate? what does this mean?<br>A: Because of the adjustment in content, this part of the analysis was removed in the new version manuscript.<br>■line776. not very convincing explanation<br>A: Because of the adjustment in content, this part of the analysis was removed in the new version manuscript.<br>■line792. please rephrase<br>A: This part of the content seemed not directly related to our results. So the analysis of this part was removed in the new version manuscript.<br>■line806. More future perspectives? which are briefly, the financial advantages of a further research of epigenetic regulation mechanisms?<br>A: In view of our findings, we will focus on the study on the act of enzymes and detailed pathway related to the CHG methylation in order to explore the much deeper mechanism of epigenetic involvement in the occurrence of Fuji bud mutation.
**Additional Information:**	

Question	Response
**Financial Disclosure**<br><br>Enter a financial disclosure statement that describes the sources of funding for the work included in this submission. Review the submission guidelines for detailed requirements. View published research articles from *PLOS ONE* for specific examples.<br><br>This statement is required for submission and **will appear in the published article** if the submission is accepted. Please make sure it is accurate.<br><br>**Unfunded studies**<br>Enter: *The author(s) received no specific funding for this work.*<br><br>**Funded studies**<br>Enter a statement with the following details:<br>• Initials of the authors who received each award<br>• Grant numbers awarded to each author<br>• The full name of each funder<br>• URL of each funder website<br>• Did the sponsors or funders play any role in the study design, data collection and analysis, decision to publish, or preparation of the manuscript?<br>• **NO** - Include this sentence at the end of your statement: *The funders had no role in study design, data collection and analysis, decision to publish, or preparation of the manuscript.*<br>• **YES** - Specify the role(s) played.<br><br>* typeset	This research was supported by "Double-Hundred Talent Plan" on 100 Foreign Experts and 100 Foreign Expert Teams Introduction in Shandong Province (WST2018013), Key Research and Development project of Shandong Province (2018GHZ005), Shandong Provincial Agricultural Variety Improvement Project (2019LZGC007), and Taishan scholar seed industry talent team construction project in Shandong Province.
**Competing Interests**<br><br>Use the instructions below to enter a competing interest statement for this submission. On behalf of all authors, disclose any competing interests that could be perceived to bias this work—acknowledging all financial support and any other relevant financial or non-financial competing interests.<br><br>This statement **will appear in the published article** if the submission is	The authors have declared that no competing interests exist.

Powered by Editorial Manager® and ProduXion Manager® from Aries Systems Corporation

accepted. Please make sure it is accurate. View published research articles from *PLOS ONE* for specific examples.<br><br>**NO authors have competing interests**<br><br>Enter: *The authors have declared that no competing interests exist.*<br><br>**Authors with competing interests**<br><br>Enter competing interest details beginning with this statement:<br><br>*I have read the journal's policy and the authors of this manuscript have the following competing interests: [insert competing interests here]*<br><br>* typeset	
**Ethics Statement**<br><br>Enter an ethics statement for this submission. This statement is required if the study involved:<br><br>• Human participants<br>• Human specimens or tissue<br>• Vertebrate animals or cephalopods<br>• Vertebrate embryos or tissues<br>• Field research<br><br>Write "N/A" if the submission does not require an ethics statement.<br><br>General guidance is provided below. Consult the submission guidelines for detailed instructions. **Make sure that all information entered here is included in the Methods section of the manuscript.**	Yantai Academy of Agricultural Sciences

**Format for specific study types**<br><br>**Human Subject Research (involving human participants and/or tissue)**<br>• Give the name of the institutional review board or ethics committee that approved the study<br>• Include the approval number and/or a statement indicating approval of this research<br>• Indicate the form of consent obtained (written/oral) or the reason that consent was not obtained (e.g. the data were analyzed anonymously)<br><br>**Animal Research (involving vertebrate animals, embryos or tissues)**<br>• Provide the name of the Institutional Animal Care and Use Committee (IACUC) or other relevant ethics board that reviewed the study protocol, and indicate whether they approved this research or granted a formal waiver of ethical approval<br>• Include an approval number if one was obtained<br>• If the study involved *non-human primates*, add *additional details* about animal welfare and steps taken to ameliorate suffering<br>• If anesthesia, euthanasia, or any kind of animal sacrifice is part of the study, include briefly which substances and/or methods were applied<br><br>**Field Research**<br><br>Include the following details if this study involves the collection of plant, animal, or other materials from a natural setting:<br>• Field permit number<br>• Name of the institution or relevant body that granted permission	
**Data Availability**<br><br>Authors are required to make all data underlying the findings described fully available, without restriction, and from the time of publication. PLOS allows rare exceptions to address legal and ethical concerns. See the PLOS Data Policy and FAQ for detailed information.	Yes - all data are fully available without restriction

A Data Availability Statement describing where the data can be found is required at submission. Your answers to this question constitute the Data Availability Statement and **will be published in the article**, if accepted.<br><br>**Important:** Stating 'data available on request from the author' is not sufficient. If your data are only available upon request, select 'No' for the first question and explain your exceptional situation in the text box.<br><br>Do the authors confirm that all data underlying the findings described in their manuscript are fully available without restriction?	
**Describe where the data may be found in full sentences. If you are copying our sample text, replace any instances of XXX with the appropriate details.**<br><br>• If the data are **held or will be held in a public repository**, include URLs, accession numbers or DOIs. If this information will only be available after acceptance, indicate this by ticking the box below. For example: *All XXX files are available from the XXX database (accession number(s) XXX, XXX.).*<br>• If the data are all contained **within the manuscript and/or Supporting Information files**, enter the following: *All relevant data are within the manuscript and its Supporting Information files.*<br>• If neither of these applies but you are able to provide **details of access elsewhere**, with or without limitations, please do so. For example:<br><br>*Data cannot be shared publicly because of [XXX]. Data are available from the XXX Institutional Data Access / Ethics Committee (contact via XXX) for researchers who meet the criteria for access to confidential data.*<br><br>*The data underlying the results presented in the study are available from (include the name of the third party*	All relevant data are within the manuscript and its Supporting Information files.

Powered by Editorial Manager® and ProduXion Manager® from Aries Systems Corporation

and contact information or URL).<br>• This text is appropriate if the data are owned by a third party and authors do not have permission to share the data.<br><br>* typeset	
Additional data availability information:	Tick here if the URLs/accession numbers/DOIs will be available only after acceptance of the manuscript for publication so that we can ensure their inclusion before publication.

Powered by Editorial Manager® and ProduXion Manager® from Aries Systems Corporation

Dear Editors:

We would like to submit the enclosed manuscript entitled 'The assessment of epigenetic diversity, differentiation, and structure in the 'Fuji' mutation line implicates roles of epigenetic modification in the occurrence of different mutant groups as well as spontaneous mutants', which we wish to be considered for publication in PLOS ONE.

DNA methylation is connected with numerous biological processes. At present, the investigations on DNA methylation are generally divided into specific site methylation and global DNA methylation. In fruit crops, literatures on the former are much than that on the latter. But overall, the study of DNA methylation in fruit crops is just beginning. Bud mutation is a rapid and effective source of materials for the breeding of new varieties in perennial woody fruit crops. However, it usually presents low occurrence frequency and low optimal rate of change under natural conditions. Therefore, the excellent commercial variation surely reflects the excellent adaptability to climate and environmental factors. The study on the characteristics and mechanisms of bud mutation occurrence will be of great significance for clarifying the causes of the mutation and further breeding by artificial inducement. Studies on DNA methylation of *Myb* transcription factor have shown that methylation might be one of the causes of bud mutations in fruit crops, but knowledge of their global DNA methylation is poorly understood. Moreover, up to now, in available literatures, the test samples for studying bud mutation were quite limited, which generally included the mutant and control. Obviously, small sample size was insufficient to reflect the DNA methylation characteristic of bud mutations. In apple, 'Fuji' is one of the cultivars prone to mutation, which provides us with ideal and unique test material for study of bud mutation occurrence. In this paper, on the basis of our collection of 'Fuji' bud mutations and heterotopic transplanting in the same resource garden at the early stage, the bud mutations from the same parent 'Fuji' were applied on a large scale for the first time, and the epigenetic characteristics of bud mutations were investigated in detail at the individual and population levels. It was found that, despite genealogical origin from the same parent, there was considerable variation in terms of DNA methylation levels between individuals and methylation patterns. Moreover, the total variation of methylation levels and patterns among the three mutant groups classified according to different mutation traits, as well as the variation degree and emphasis within the groups also showed diversity characteristics. Bud mutations are thought to have very similar genetic backgrounds, but this was the first study to explain the rich and distinct epigenetic characteristics between them. Our study will provide help for future study on epigenetic mechanism of bud mutation occurrence in 'Fuji' apple.

Neither the entire paper nor any part of its content has been published or has been accepted elsewhere. It is not being submitted to any other journal. We hope the manuscript could be considered for publication in PLOS ONE. Thank you very much for your reading. We are looking forward to hearing from you.

Data Availability: All relevant data are within the paper and its Supporting Information files.

Sincerely,

Xiaoyun Du, Yanbo Wang, Xueqing Liu, Zhongwu Jiang, Lingling Zhao, Yan Tang, Yanxia Sun, Xueyong Zhang, Daliang Liu, Laiqing Song

Manuscript Click here to access/download;Manuscript;Manuscript.docx

The assessment of epigenetic diversity, differentiation, and structure in the 'Fuji' mutation line implicates roles of epigenetic modification in the occurrence of different mutant groups as well as spontaneous mutants

Xiaoyun Du[1¶], Yanbo Wang[1¶], Minxiao Liu[1], Xueqing Liu[1], Zhongwu Jiang[1,2], Lingling Zhao[1,2], Yan Tang[1], Yanxia Sun[1], Xueyong Zhang[1], Daliang Liu[1], Laiqing Song[1*]

1 Yantai Academy of Agricultural Sciences, Yantai, Shandong, China

2 Yantai University, Yantai, Shandong, China

* Corresponding author

E-mail: sdslq@126.com

[¶]These authors contributed equally to this work.

Abstract

The 'Fuji' line includes many varieties with a similar genetic background and consistent inducement factors with epigenetic occurrence, thus it may be considered an ideal candidate for epigenetic research. In this study, 91 bud mutations of 'Fuji' apple were used as the test materials. Using the genetic variation within 'Fuji' as the control, the

characteristics of epigenetic variation at different levels in both varieties and mutant groups were examined. The results showed that: (1) the global genomic DNA methylation level of the 91 bud mutants of ‘Fuji’ ranged from 29.120%-45.084%, with an average of 35.910%. Internal cytosine methylation was the main DNA methylation pattern. Regarding the variation of methylation patterns of ‘Fuji’ mutants, the vast majority of loci maintained the original methylation pattern existed in ‘Fuji’. CHG methylation variation was the main type of variation. (2) The variation in methylation patterns between the mutant groups was greater than that of methylation levels. Among these patterns, the variation in CHG methylation patterns (including CHG hypermethylation and CHG demethylation) was expected to be dominant. The observed variation in methylation levels was more important in the Color mutant group; however, the variation in methylation patterns was more obvious in both the early maturation and Spur mutant groups. Moreover, the range of variation in the Early-maturation group was much wider than that in the Spur mutant group. (3) Epigenetic diversity and genetic diversity were both low between the mutant groups. In the ‘Fuji’ mutant groups, there was few correlation between genetic and epigenetic variation, and epigenetic differentiation resulted in more loci with moderate or greater differentiation. (4) Purifying selection seemed to play a major role in the differentiation of different groups of ‘Fuji’ mutants (65.618%), but epigenetic diversity selection still occurred at nearly 35% of loci. Sixteen epigenetic outlier loci were detected.

Introduction

Malus domestica Borkh. cv. ‘Fuji’ is one of the most economically important apple

varieties in China and the world. ‘Fuji’ apple is prone to mutation, through which abundant bud mutants have been derived, the majority of which have been adapted as superior varieties in production [1]. Studies have shown that epigenetics is an important cause of bud mutations in fruit crops, but it has not yet to be systematically studied in a large sample size. The study of the characteristics of epigenetic effects in bud mutants is the premise for further studying the epigenetic mechanism of mutations and carrying out epigenetic breeding.

Epigenetics studies the heritable changes in gene function that eventually lead to phenotypic variation with no changes in the underlying DNA sequence [2]. Epigenetics is involved in the regulation of gene expression, and changes are dynamic with respect to the endogenous and/or external environmental stimuli, thus affecting the phenotypic plasticity and environmental adaptability of organisms [3]. Epigenetic variation enhances biodiversity and complexity, especially in asexual organisms without gene recombination, which is helpful to the promotion of functional phenotypic diversity and has a greater impact on variation and evolution and more survival significance [4].

DNA methylation, representing the most important form of epigenetic modification, is ubiquitous in higher plants [5]. Different level of DNA methylation exists in normal organisms (5%-40%, [6]), while changes in DNA methylation can be caused by external environmental factors or various biotic and abiotic stresses [7]. Changes in DNA methylation level and methylation pattern may cause significant phenotypic variation in plant genome [8]. It can be expressed by the dynamics of hypermethylation and demethylation [9]. DNA methylation occurs on cytosines in different sequence contexts,

and CG and CHG are the two main types of genome methylation. CG and CHG methylation are regulated by distinct enzymes and pathways [10]. Both CG and CHG methylation levels are different in a plant species, and CG methylation was reported predominately in many plant species [11].

The current research on DNA methylation is mainly focused on local and global aspects. The former mainly targets specific traits, starting with the changes directly related gene methylation in promoter or gene core sequence, and has more advantages in revealing the mechanism of occurrence of specific phenotypic variation; The latter studies the changes in the overall methylation of the genome, which is more conducive to the comprehensive analysis of the role of epigenetics in the variation and diversity. Many techniques have been developed to analyze global DNA methylation and its alterations [12]. The method of methylation-sensitive amplified polymorphism (MSAP) is one of the most efficient, economical, and widely one used for the detection of DNA methylation events [13-14]. This approach is a modified version of the amplified fragment length polymorphism (AFLP) method based on the differential sensitivity of isoschizomeric restriction endonucleases to site-specific cytosine methylation [15]. MSAP analysis employs two types of cleaved enzymes *Eco*RI (rare cutter) and *Hpa*II/*Msp*I (frequent cutters) which recognize similar tetranucleotide 5'-CCGC representing a different sensitivity to methylation at the inner or outer cytosine. If one or both cytosines are methylated at both DNA strands, *Hpa*II is inactive. If one or both cytosines are methylated in only one strand they are cleaved by *Hpa*II. In contrast, *Msp*I reacts when only the internal cytosine is hemi- or fully- (double strand) methylated [16].

So, on the basis of variant band patterns resulting from differential digestion of the genome by *Hpa*II/*Msp*I isozymes, variation in the DNA methylation level and pattern in the whole genome is detected [15, 17]. MSAP has been successfully applied for the analysis of the variation in methylation levels and patterns in a variety of plant species [13].

In general, bud mutations are an important source of new varieties of fruit crops. Characteristics such as a perennial nature, long juvenile phase, heterozygosity, and sexual incompatibilities in fruit crops hamper their improvement through conventional breeding [18]. Compared with conventional hybridization, the selection of bud mutants gives the advantages of shortening the breeding cycle and reducing the workload and costs. Such an approach can be used to obtain excellent varieties by modifying individual traits without changing the desirable qualities of the parent plant [19]. A variety of perennial fruit trees of economic importance originated from bud changes [20-21]. However, Spontaneous bud mutation occurs at a very low frequency, moreover, many of these mutations may be deleterious, making the organism less adapted to its environment, and in some cases may even be lethal [18]. Therefore, mutants that survive in adverse environments and even present excellent phenotypes are considered to have good characteristics for adaption. Epigenetic regulation mediated by DNA methylation is considered as one of the important mechanisms in plant adaptive procedure [14, 22]. Studying the molecular mechanism of bud mutation at the DNA level is thus of great significance.

Fuji is the most representative apple cultivar in which plenty of new bud mutations

arise [23]. Multiple bud mutants generated from the standard cultivar Fuji. Those represent highly similar genetic backgrounds and have abundant types of variation. Additionally, Fuji bud mutants were frequently reported to be available in orchards at unusual locations, such as under dense high-voltage lines, at high altitudes, subjected to an abnormal climate, severe drought, waterlogging, frost, or sudden diseases or insect pest infestations [24]. This means that the conditions for induction of bud mutation are consistent with the factors inducing epigenetic effects. Therefore, in a summary of the above mentioned, Fuji mutation line could be regarded as ideal sets for research on epigenetic regulation. However, to our knowledge, global DNA methylation in this line has not been reported yet.

A general understanding of the mechanisms of genome-wide DNA methylation in ‘Fuji’ mutants is a prerequisite for their utilization in epigenetic mechanism studies or breeding. Therefore, in the present study, nearly one hundred ‘Fuji’ bud mutants were used as study materials for the first time. Then, through genetic variation as control, we focused on the analysis of variations in DNA methylation levels and patterns within groups and between groups as well as epigenetic diversity.

We were interested in the following questions: (1) is DNA methylation involved in the occurrence of bud mutations in the ‘Fuji’ line? (2) what are the characteristics of the changes in both DNA methylation levels and patterns? (3) what are the effects of DNA methylation on not only the clustering status of the ‘Fuji’ line at the variety level but also genetic structure and differentiation at the group level? (4) how is natural selection affecting the occurrence of different mutant groups in the Fuji line?

Materials and Methods

Plant material and MSAP, AFLP analyses

This study was conducted on ‘Fuji’ and it’s 91 elite varieties arising from bud mutations. Three major types: Color mutant group, Early-maturation mutant group, and Spur mutant group could be classified (Table 1). We sampled plant materials with the same age on the same dates (12 June 2016) and at their identical phenological stage (bearing fully expanded leaves), trying to avoid that possible developmental variation in DNA methylation would confound variety or group differences in methylation patterns. Fully expanded fresh leaves from pooled individuals (ca. 5 plants being clonally propagated from a single mother variety) of each variety were collected. Young leaves immediately frozen in liquid nitrogen and then stored at -80℃ prior to DNA isolation.

Total genomic DNA from leaf tissue was extracted by using DNeasy Plant Mini Kit (Qiagen). MSAP molecular marker was used for the analysis of epigenetic variation in plants examined. For comparison, genetic variation was also conducted by AFLP molecular marker. Both methods performed on the same set of plants, and *Eco*RI primers were labeled with or green (JOE), blue (FAM), or yellow (NED) fluorescent dyes.

MSAP and AFLP were performed as described by Xiong et al. [17] and Vos etal. [25], respectively. MSAP was essentially the same as the AFLP protocol. The difference between MSAP and AFLP procedure was replacing the *Mse*I enzyme with the enzyme either *Hpa*II or *Msp*I in MSAP. Thus, differences in the PCR products detected with *Eco*RI/*Hpa*II and *Eco*RI/*Msp*I would reflect different methylation states.

The primer sequences of MSAP and AFLP were listed in the S1 Table. Based on previous pilot tests, we selected 23 optimal primer combinations for MSAP analysis (S2 Table) and 19 optimal primer combinations for the AFLP analysis (S3 Table). HM-, E-, and M- corresponded to the sequence of H/M00, E00, and M00, respectively (S1 Table). The repeatability of banding patterns assessed by conducting two sets of independent MSAP and AFLP analyses and only the consistent bands were included.

Using an automated sequencer (ABI PRISM®3730 Genetic Analyzer, Applied Biosystems) to separated and detected MSAP and AFLP PCR products. GeneScan Rox-500 labeled with a red (ROX) dye was an internal size marker. A peak size between 80 and 500 bp was selected to study the polymorphic DNA fragments. MSAP products were scored as present '1' or absent '0' on the chromatogram to create a binary matrix.

Statistical analysis

The resulting data of MSAP and AFLP were processed using Excel 2016. According to the scoring approach [26], MSAP raw data were transformed into a binary data matrix before running statistical analyses and computation. Because the enzymes used for MSAP analysis recognize the same restriction site (5'-CCGG) but have different sensitivities to methylation modifications, the final products of MSAP present four types of DNA methylation at the 5'-CCGG sites, namely, no methylation (H1M1, condition I), CHG methylation (H1M0, condition II), CG methylation (H0M1, condition III), and CG/CHG methylation (H0M0, condition IV). DNA methylation level%= (condition II+condition III)/(condition I+condition II+condition III+condition IV).

Methylation-Sensitive Polymorphism (MSP) matrix data, which were converted using R program *msap* [27], were used for all analyses of DNA methylation. The change of patterns of cytosine methylation at CCGG sites in the 'Fuji' mutation line was listed in Table 2. Accordingly, contrasting to the standard cultivar 'Fuji', the changes of DNA methylation pattern in its series mutations could been summarized into four categories: (a) CG hyper: H1M1 to H0M1, H1M0 to H0M0, H1M0 to H0M1, H1M1 to H0M0; (b) CHG hyper: H1M1 to H1M0, H0M1 to H0M0, H1M1 to H0M0, H0M1 to H1M0; (c) CG hypo: H0M1 to H1M1, H0M0 to H1M0, H0M1 to H1M0; and (d) CHG hypo: H1M0 to H1M1, H0M0 to H0M1, H1M0 to H0M1, H0M0 to H1M1.

DNA methylation analysis and variation coefficient calculation of different mutant groups in 'Fuji'

The epigenetic relationship of different mutant groups in 'Fuji' was analyzed with 12 indexes related to DNA methylation levels and patterns. Indicators include CHG hyper-methylation frequency (V5), CG hyper-methylation frequency (V6), CG hypo-methylation frequency (V7), CHG hypo-methylation frequency (V8), the total hyper-methylation frequency (V9), the total hypo-methylation frequency (V10), total genome methylation frequency (V11), the frequency of condition I (Non-methylation frequency, V12), the frequency of condition II methylation (CHG methylation frequency, V13), the frequency of condition III methylation (CG methylation ratio, V14), the frequency of condition IV methylation (V15), and the total amplified loci of varieties (V16).

The Coefficient of Variation (CV) of each of the above indexes was calculated in the three mutation groups in order to investigate whether varieties in different mutant

groups behave in different ways. CV=(standard deviation SD/Mean)×100%. Correlation analysis of 12 major epigenetic parameters (V5-V15) was performed by software IBM SPSS Statistics 22 [28].

Epigenetic and genetic similarity, clustering and principal component analysis

The similarity coefficient between varieties was calculated using the SM coefficient through the SimQual procedure of NTSYSpc2.11 software package [29] and unweighted pair group method average (UPGMA) method was used for cluster analysis. Circle diagrams were drawn using TBtools [30]. MSAP-PCA and AFLP-PCA analysis were carried out by R program package *msap* 27 and Adegenet 2.1.1 [31], respectively.

Epigenetic and genetic diversity and molecular variation analysis

Various diversity measurement parameters, including polymorphism site proportion (PPL), effective allele variance (Ne), Shannon diversity index (I), expected heterozygosity (He), and hierarchical analysis of molecular variance (AMOVA) analysis between and within mutant groups were estimated in GenAlex 6.51 [32], using 999 random permutations.

Epigenetic and genetic Structural analysis

STRUCTURE 2.3.4 [33] was utilized to analyze genetic and epigenetic structure. Admixture and correlated allele frequencies model were chosen. Ten independent runs were made with values of K set from 2 to 4, with three iterations for each value of K. The length of the burn-in period was set at 10,000, and the number of Markov chain

Monte Carlo (MCMC) repeats after burn-in was set at 100,000. The result from STRUCTURE output file was performed online by STRUCTRE Harvester 0.6.8 [34]. The results were averaged for a particular K using CLUMPP 1.1.2 [35] and visualized by DISTRUCT [36].

Potential outlier detection

BAYESCAN v2.1 [37] was used to test Fst outliers in global and pairwise comparisons. A reversible-jump Markov chain Monte Carlo algorithm based on a Bayesian likelihood approach is used in BAYESCAN to estimate the ratio of posterior probabilities of selection over neutrality [the posterior odds (PO)]. Based on Jeffreys' [38] scale of evidence, a $\log_{10}PO > 2.0$ is interpreted as 'strong evidence' of selection. For our analysis, the estimation of model parameters was set as 10 pilot runs of 5,000 interaction each, followed by 100,000 interactions [37]. Outliers were calculated using a burn-in of 50,000 interactions, a thinning interval of 20, and a sample size of 5000. FDR=0.05 was used.

Correlation analysis between epigenetic diversity and genetic diversity

Using NTSYS2.0 software, the correlations between epigenetic distance and genetic distance of three 'Fuji' mutant groups as well as varieties calculated by MSAP and AFLP were evaluated using the Mantel test implemented through NTSYSpc 2.11 software package [28].

All of the statistical significance in this study was determined by IBM SPSS Statistics 22 [32]. A value of $P < 0.05$ was considered significant.

Results

MSAP and AFLP amplification

A total of 2954 CCGG loci were detected via the genome-wide methylation analysis of 92 ‘Fuji’ varieties with 23 pairs of MSAP primer combinations, and 129 CCGG loci were amplified on average with each pair of primers. Among these loci, 2752 CCGG loci showed polymorphism, for a polymorphic ratio of 93.162%. Different MSAP amplification patterns were obtained from different varieties (Table 1). The number of amplified methylated loci ranged from 1793 to 2136, and that of polymorphic loci ranged from 1613 to 1956, with an average polymorphic ratio of 63.566% (58.612% to 71.076%). Among the 2954 CCGG loci, 1748 were methylation-sensitive loci (MSL), accounting for 59.174% of the total amplified loci. A total of 1627 polymorphic MSL loci were obtained, accounting for 93.078% of the total amplified loci (S2 Table).

Table 1. Plant materials and summary of MSAP amplification in this study.

Variety Code	Name	Mutation type	Total loci	Non-methylated CCGG loci (%)	Methylated CCGG loci (%)		
					CG-methylated	CHG- methylated	Total
M1	Fuji	Origin	1841	1212(65.834)	396(21.510)	233(12.656)	629(34.166)
M2	Shanfu No.6	Color	2112	1227(58.097)	402(19.034)	483(22.869)	885(41.903)
M3	Iwate line Ⅰ	Color	1880	1134(60.319)	475(25.266)	271(14.415)	746(39.681)
M4	Iwafu No.10	Color	1904	1163(61.082)	478(25.105)	263(13.813)	741(38.918)

M5	Line Ⅰ Fuji	Color	1996	1136(56.914)	552(27.655)	308(15.431)	860(43.086)
M6	Gunfu No.1	Color	1875	1268(67.627)	363(19.360)	244(13.013)	607(32.373)
M7	Aki Fuji	Color	1893	1231(65.029)	453(23.930)	209(11.041)	662(34.971)
M8	Lele Fuji	Color	1962	1222(62.283)	497(25.331)	243(12.385)	740(37.717)
M9	Aomori-fu No.13	Color	2025	1263(62.370)	363(17.926)	399(19.704)	762(37.630)
M10	2001	Color	1874	1231(65.688)	396(21.131)	247(13.180)	643(34.312)
M11	Aki-fu No.1	Color	1872	1238(66.132)	438(23.397)	196(10.470)	634(33.868)
M12	Aki-fu No.5	Color	1910	1270(66.492)	433(22.670)	207(10.838)	640(33.508)
M13	Morioka-fu No.1	Color	2018	1294(64.123)	363(17.988)	361(17.889)	724(35.877)
M14	Morioka-fu No.2	Color	1950	1289(66.103)	373(19.128)	288(14.769)	661(33.897)
M15	Tensei	Color	1989	1260(63.348)	471(23.680)	258(12.971)	729(36.652)
M16	Qingnonghe No.2	Color	1916	1288(67.223)	387(20.198)	241(12.578)	628(32.777)
M17	Qianxuan No.3	Color	1908	1263(66.195)	380(19.916)	265(13.889)	645(33.805)
M18	Fubrax	Color	1871	1231(65.794)	411(21.967)	229(12.239)	640(34.206)
M19	Nagafu No.12	Color	1939	1287(66.374)	353(18.205)	299(15.420)	652(33.626)
M20	Nagafu No.1	Color	1923	1266(65.835)	341(17.733)	316(16.433)	657(34.165)
M21	Nagafu No.2	Color	1998	1281(64.114)	406(20.320)	311(15.566)	717(35.886)
M22	Nagafu No.4	Color	1928	1272(65.975)	399(20.695)	257(13.330)	656(34.025)
M23	Nagafu No.6	Color	2026	1297(64.018)	372(18.361)	357(17.621)	729(35.982)
M24	Nagafu No.7	Color	1933	1322(68.391)	377(19.503)	234(12.106)	611(31.609)
M25	Nagafu No.36	Color	1904	1201(63.078)	464(24.370)	239(12.553)	703(36.922)

M26	Wengao No.1	Color	1871	1254(67.023)	379(20.257)	238(12.720)	617(32.977)
M27	Wengao-M No.1	Color	1968	1302(66.159)	450(22.866)	216(10.976)	666(33.841)
M28	Wengao No.2	Color	1884	1317(69.904)	332(17.622)	235(12.473)	567(30.096)
M29	Wengao-M No.2	Color	1917	1267(66.093)	358(18.675)	292(15.232)	650(33.907)
M30	Wengao-M No.3	Color	2066	1270(61.471)	438(21.200)	358(17.328)	796(38.529)
M31	Yanfu No.1	Color	1976	1233(62.399)	440(22.267)	303(15.334)	743(37.601)
M32	Yanfu No.2	Color	1940	1249(64.381)	457(23.557)	234(12.062)	691(35.619)
M33	Yanfu No.3	Color	1952	1278(65.471)	449(23.002)	225(11.527)	674(34.529)
M34	Yanfu No.4	Color	1962	1243(63.354)	429(21.865)	290(14.781)	719(36.646)
M35	Yanfu No.5	Color	2014	1300(64.548)	435(21.599)	279(13.853)	714(35.452)
M36	Yanfu No.8	Color	1915	1233(64.386)	445(23.238)	237(12.376)	682(35.614)
M37	Yanfu No.10	Color	1921	1267(65.955)	433(22.540)	221(11.504)	654(34.045)
M38	92-58	Color	2103	1383(65.763)	440(20.922)	280(13.314)	720(34.237)
M39	Meili	Color	1933	1298(67.150)	404(20.900)	231(11.950)	635(32.850)
M40	Meile	Color	2013	1272(63.189)	503(24.988)	238(11.823)	741(36.811)
M41	Tianfu No.1	Color	2004	1310(65.369)	385(19.212)	309(15.419)	694(34.631)
M42	Tianfu No.2	Color	1980	1270(64.141)	462(23.333)	248(12.525)	710(35.859)
M43	Yanchanghong	Color	1947	1259(64.664)	446(22.907)	242(12.429)	688(35.336)
M44	Zhaoyuanstripe red Fuji	Color	2021	1304(64.523)	458(22.662)	259(12.815)	717(35.477)
M45	Zhaoyuanflush red Fuji	Color	2136	1247(58.380)	610(28.558)	279(13.062)	889(41.620)
M46	Shoufu No.1	Color	2031	1281(63.072)	517(25.455)	233(11.472)	750(36.928)

M47	Shoufu No.2	Color	2039	1309(64.198)	518(25.405)	212(10.397)	730(35.802)
M48	Yulindian Fuji	Color	1978	1242(62.791)	493(24.924)	243(12.285)	736(37.209)
M49	Changfujia	Color	2035	1282(62.998)	426(20.934)	327(16.069)	753(37.002)
M50	Zhaofuwang	Color	1885	1290(68.435)	339(17.984)	256(13.581)	595(31.565)
M51	Yishuihong	Color	1893	1160(61.278)	439(23.191)	294(15.531)	733(38.722)
M52	Jihong	Color	1904	1259(66.124)	450(23.634)	195(10.242)	645(33.876)
M53	Yannongzaofu	Early-maturation	1793	1141(63.636)	423(23.592)	229(12.772)	652(36.364)
M54	Zaoshu Fuji	Early-maturation	1884	1116(59.236)	490(26.008)	278(14.756)	768(40.764)
M55	Early Fuji A	Early-maturation	1965	1203(61.221)	471(23.969)	291(14.809)	762(38.779)
M56	Hongjiangjun	Early-maturation	1862	1202(64.554)	411(22.073)	249(13.373)	660(35.446)
M57	Hongwangjiang	Early-maturation	1804	1222(67.738)	301(16.685)	281(15.576)	582(32.262)
M58	Jinfu No.1	Early-maturation	1927	1201(62.325)	491(25.480)	235(12.195)	726(37.675)
M59	Gai Fuji	Early-maturation	1851	1169(63.155)	405(21.880)	277(14.965)	682(36.845)
M60	Yuhuazaofu	Early-maturation	1816	1149(63.271)	391(21.531)	276(15.198)	667(36.729)
M61	Qianxuan No.1	Early-maturation	1847	1220(66.053)	408(22.090)	219(11.857)	627(33.947)
M62	Qianxuan No.2	Early-maturation	1889	1226(64.902)	450(23.822)	213(11.276)	663(35.098)
M63	Changhong	Early-maturation	1923	1231(64.015)	393(20.437)	299(15.549)	692(35.985)
M64	New ryoka	Early-maturation	1884	1219(64.703)	411(21.815)	254(13.482)	665(35.297)
M65	Ryoka	Early-maturation	1862	1165(62.567)	459(24.651)	238(12.782)	697(37.433)
M66	Jinfu No.2	Early-maturation	1855	1213(65.391)	405(21.833)	237(12.776)	642(34.609)
M67	Jinfu No.3	Early-maturation	1892	1217(64.323)	381(20.137)	294(15.539)	675(35.677)

M68	Yishuizhongqiu	Early-maturation	1892	1200(63.425)	448(23.679)	244(12.896)	692(36.575)
M69	Fengfuji No.1	Early-maturation	1888	1160(61.441)	452(23.941)	276(14.619)	728(38.559)
M70	Shoufu No.3	Early-maturation	1928	1232(63.900)	472(24.481)	224(11.618)	696(36.100)
M71	Changyanghong	Early-maturation	2057	1458(70.880)	340(16.529)	259(12.591)	599(29.120)
M72	Yiyuan Nagafu No.2	Early-maturation	1883	1161(61.657)	452(24.004)	270(14.339)	722(38.343)
M73	Hirosaki fuji	Early-maturation	1975	1181(59.797)	565(28.608)	229(11.595)	794(40.203)
M74	Karakida Fuji	Early-maturation	2120	1265(59.670)	497(23.443)	358(16.887)	855(40.330)
M75	Sufuji	Early-maturation	1867	1201(64.328)	381(20.407)	285(15.265)	666(35.672)
M76	Qiufuhong	Spur	2085	1145(54.916)	614(29.448)	326(15.635)	940(45.084)
M77	Yanfu No.6	Spur	1818	1235(67.932)	367(20.187)	216(11.881)	583(32.068)
M78	Yanfu No.7	Spur	1820	1194(65.604)	418(22.967)	208(11.429)	626(34.396)
M79	Huimin spur	Spur	1882	1219(64.772)	440(23.379)	223(11.849)	663(35.228)
M80	Duanzhi Fuji	Spur	1852	1222(65.983)	390(21.058)	240(12.959)	630(34.017)
M81	Miyazaki	Spur	1853	1200(64.760)	399(21.533)	254(13.708)	653(35.240)
M82	Tiaowen Gongqi	Spur	2000	1150(57.500)	583(29.150)	267(13.350)	850(42.500)
M83	Aki-fu No.39	Spur	1934	1182(61.117)	452(23.371)	300(15.512)	752(38.883)
M84	Longfu	Spur	1853	1198(64.652)	399(21.533)	256(13.815)	655(35.348)
M85	Fukushima spur	Spur	1883	1216(64.578)	408(21.668)	259(13.755)	667(35.422)
M86	Aomori spur	Spur	1884	1217(64.597)	461(24.469)	206(10.934)	667(35.403)
M87	Qinfu No.1	Spur	1941	1241(63.936)	445(22.926)	255(13.138)	700(36.064)
M88	Liquan spur	Spur	1840	1177(63.967)	411(22.337)	252(13.696)	663(36.033)

M89	Lingbaoduanfu	Spur	1842	1182(64.169)	440(23.887)	220(11.944)	660(35.831)
M90	Chengji No.1	Spur	1857	1189(64.028)	434(23.371)	234(12.601)	668(35.972)
M91	Yiyuanhong	Spur	1942	1252(64.470)	376(19.361)	314(16.169)	690(35.530)
M92	Shifu spur	Spur	1814	1187(65.436)	413(22.767)	214(11.797)	627(34.564)
**Total**	**Min**		1793	1116(54.916)	301(16.528)	195(10.242)	567(29.120)
	**Max**		2136	1458(70.880)	614(29.448)	483(22.870)	940(45.084)
	**Mean**		1930	1236(64.090)	431(22.311)	263(13.599)	694(35.910)

Nineteen pairs of AFLP primer combinations amplified 1745 total loci in the same test varieties used for MSAP, among which 1620 were polymorphic loci, accounting for 92.837% of the total amplified loci. Different primer combinations produced different amplification results. The total number of amplified loci obtained with a single pair of primers ranged from 36 to 166, the number of polymorphic loci ranged from 28 to 150, and the polymorphic ratio ranged from 57.143% to 100%. On average, the total numbers of loci and polymorphic loci generated by amplification with each pair of primers were 92 and 86, respectively. The four primer combinations (E-AGG+M-CAG, E-AGG+M-CTA, E-ACG+M-CAC, E-ACG+M-CTG) produced 100% polymorphic loci. Primer amplification details are shown in S3 Table.

Analysis of DNA methylation levels and variation patterns at the variety level

As shown in Table 1, the global genomic DNA methylation level of the 91 varieties was 29.120%-45.084%, with an average of 35.929%. Among these modifications, the

internal cytosine methylation level was 22.311% (16.528%-29.448%), and the external cytosine methylation level was 13.599% (10.242%-22.870%). The former was significantly higher than the latter (*P*<0.01), implying that internal cytosine methylation was the main DMA methylation way in the 'Fuji' line.

According to the cutting profiles of the *Hpa*II and *Msp*I methylation-sensitive endonucleases in the original 'Fuji' variety, the banding patterns could be divided into four types: A, B, C, and D (Table 2). In comparison with the original 'Fuji', there were many types of possible locus variation in the mutant varieties, so the variation pattern of methylation loci could be subdivided into several subcategories: A1, A2, A3, A4, B1, B2, B3, B4, C1, C2, C3, C4, D1, D2, and D3. As shown in S1 Fig., in DNA methylation variation patterns A and C, loci identical to the original methylation pattern presented the highest proportion, indicating that during the occurrence of bud mutations in 'Fuji' line, the majority of loci maintained the original methylation pattern, while only a few loci exhibited methylation variation (S5 Table). All types of methylation variation patterns (15 subclasses) were detected in the test varieties.

Table 2. Changes in the patterns of cytosine methylation at CCGG sites in the 'Fuji' mutation line.

**Pattern**		**Band type digested by E+H /E+M**		**Pattern**		**Band type digested by E+H /E+M**	
**Type**	**Sub-type**	**Fuji**	**mutation**	**Type**	**Sub-type**	**Fuji**	**Mutation**
A	A1	+/+	+/-	C	C1	-/+	+/-
	A2	+/+	+/+		C2	-/+	+/+

	A3	+/+	-/+		C3	-/+	-/+
	A4	+/+	-/-		C4	-/+	-/-
B	B1	+/-	+/-	D	D1	-/-	+/-
	B2	+/-	+/+		D2	-/-	+/+
	B3	+/-	-/+		D3	-/-	-/+
	B4	+/-	-/-				

Notes: (1) E: *Eco*R Ⅰ enzyme; H: *Hpa*II enzyme; M: *Msp*I enzyme; (2) +: band present; -: band absent; +/+: band present in both E+H and E+M; +/-: band present in E+H but absent in E+M; -/+: band absent in E+H but present in E+M; -/-: band absent in both E+H and E+M.

Fig 1. Correlation analysis of 12 epigenetic parameters. Three asterisks represent a very significant correlation (P < 0. 01). The data distribution is displayed on the diagonal of the matrix; the bivariate scatter with the fitting line is displayed in the lower left; the correlation coefficient and the significance level are displayed in the upper right.

The above 15 subclasses of methylation variation patterns were further categorized into four types: CG hypermethylation (CG-hyper), CHG hypermethylation (CHG-hyper), CG demethylation (CG-hypo) and CHG demethylation (CHG-hypo). As shown in S4 Table, CHG-hypo, CHG-hyper, CG-hyper, and CG-hypo displayed differences among different tested varieties, but the general trend basically showed the following correlations: CHG-hypo>CHG-hyper>CG-hyper>CG-hypo (S2 Fig.). (CHG-hypo+CHG-hyper) was significantly higher than (CG-hypo+CG-hyper) (P<0.01). The relative trend between the total demethylation frequency and the hypermethylation

frequency in different varieties also exhibited diversity, including the following findings: (1) the demethylation frequency was higher than the frequency of methylation, (2) the hypermethylation frequency was higher than that of demethylation, and (3) hypermethylation and demethylation frequencies were approximately the same. These results indicated that methylation pattern variations were not fixed during the occurrence of the ‘Fuji’ mutation.

Analysis of the DNA methylation level and variation pattern at the mutant group level

The epigenetic relationships of the genomes of different mutant groups in the ‘Fuji’ line were analyzed using 12 parameters related to DNA methylation levels and patterns (V5-V16). Table 3 showed that the variation coefficients (CVs) of V5, V6, V7, V8, V9, and V10 were generally greater than those of V11, V12, V13, V14, V15, and V16. Among these 12 parameters, V5-V10 and V11-V16 reflected variations in the DNA methylation pattern and DNA methylation level, respectively. Therefore, it could be deduced that the variation of methylation patterns among varieties was greater than that of methylation levels. V11-V16, V11, V12, V15, and V16 exhibited the same degree of variation among groups, while V13 and V14 showed relatively higher CV than these groups, indicating that variation in CHG and CG methylation levels is abundant among varieties of different mutant groups in the ‘Fuji’ line.

Table 3. Variations in the levels of 12 DNA methylation parameters and pattern-related parameters in three ‘Fuji’ mutant groups.

Parameter	Std.Deviation			Mean			CV (%)		
	Spur	Early-maturation	Color	Spur	Early-maturation	Color	Spur	Early-maturation	Color
**V5**	2.033	2.776	1.802	12.722	13.571	14.608	15.977	20.454	12.338
**V6**	1.412	2.436	1.896	9.833	10.796	13.463	14.363	22.563	14.081
**V7**	1.965	2.558	2.732	6.682	7.346	10.223	29.401	34.817	26.726
**V8**	3.239	2.466	2.846	14.265	14.977	18.743	22.703	16.464	15.182
**V9**	2.848	4.946	3.342	22.555	24.367	28.072	12.626	20.299	11.907
**V10**	3.691	4.267	3.462	20.947	22.324	28.966	17.622	19.115	11.953
**V11**	3.141	2.616	2.605	36.328	36.427	35.572	8.645	7.181	7.324
**V12**	3.141	2.616	2.605	63.672	63.573	64.428	4.932	4.115	4.044
**V13**	1.523	1.583	2.464	13.187	13.77	13.679	11.548	11.497	18.016
**V14**	2.668	2.734	2.675	23.142	22.656	21.894	11.531	12.068	12.218
**V15**	2.444	2.552	2.260	35.334	34.989	32.948	6.917	7.293	6.854
**V16**	72.196	75.503	66.710	1889	1898	1959	3.823	3.978	3.406

Notes: V5, CHG hyper-methylation frequency; V6, CG hyper-methylation frequency; V7, CG hypo-methylation frequency; V8, CHG hyper-methylation frequency; V9, the total hyper-methylation frequency; V10, the total hypo-methylation frequency; V11, total genome methylation frequency; V12, the frequency of condition I (Non-methylation frequency); V13, the frequency of condition II methylation (CHG methylation frequency); V14, the frequency of condition III methylation (CG methylation ratio); V15, the frequency of condition IV methylation; V16, the total amplified loci of varieties.

The CVs of each parameter were compared between the three mutant groups and

analyzed (Table 3). It was shown that the emphases of epigenetic variation were different in different groups; for example, in the Early-maturation group, several parameters, including V5, V6, V7, V9, and V10, presented higher values than in the other groups; in the Spur group, V8 was much more prominent; in the Color group, V13 showed a remarkably high value. Based on the above results, it seemed that the variation in the methylation level was more important in the Color group. The variation in the methylation pattern was obvious in the Early-maturation and Spur groups; moreover, the extent of the variation in the Early-maturation group was much wider.

The correlations of 12 DNA methylation parameters were calculated and plotted using the R packages *psych* and *corrplot*. As presented in Fig 1, a total of 43 pairs (P<0.01) were significantly correlated with each other, among which V6 and V9 exhibited the highest significant positive correlation (0.93), followed by V5 and V9 (0.91). The lowest correlation was found for V10 and V13 (0.25), followed by V9 and V12 (0.31). The significant negative correlation was highest between V5 and V11 (-0.24), followed by V10 and V14 (-0.27). The lowest negative correlation was found for V15 and V16 (-1.00), followed by V10 and V15 (-0.74). The results showed that there was a direct positive correlation between the total hypermethylation frequency and the frequency of CG hypermethylation (that is, the higher the frequency of CG hypermethylation, the higher the frequency of total hypermethylation). The total demethylation frequency presented a small correlation with the proportion of CHG methylation but a significant negative correlation with the proportion of CG methylation (that is, the higher the proportion of CG methylation, the lower the total

demethylation frequency).

Furthermore, the above 12 DNA methylation parameters of the three mutant groups were compared and analyzed. The data distribution of all 12 variables conformed to a normal distribution and the assumptions of the statistical analysis. The variance homogeneity test showed that, with the exception of V9 (the total hypermethylation frequency), the other 11 parameters all met the requirements of homogeneity of variance. Therefore, the Tamhane's T2 and Games-Howell tests were chosen for V9, whereas the Duncan and LSD tests were chosen for the other parameters for multiple comparison analysis with SPSS software. Multiple comparative analysis among groups showed that among the three mutant groups, V5, V6, V7, V8, V9, V10, V15, and V16 presented significant differences (P<0.01), but no significant difference was found between V11, V12, V13, and V14. The average multiple comparison results revealed that the differences between the Spur and Early-maturation groups were not significant, showing similar epigenetic characteristics, but different degrees of significant differences existed between the Spur and Color mutant groups as well as between the Early-maturation and Color groups. For example, V5, V6, V7, V8, V9, V10, V15, and V16 were significantly different between the Spur and Color mutant groups; the difference in V6, V7, V8, V9, V10, V15, and V16 between the Early-maturation and Color mutant groups was very significant (Fig 2). Taken together, the above results showed that among the three ‘Fuji’ mutant groups, the Spur and Early-maturation groups showed similar epigenetic patterns; however, between the Color groups and either the Spur group or the Early-maturation group, epigenetic differences were

apparent.

Fig 2. AMOVA of 12 epigenetic parameters between different mutant groups in the 'Fuji' mutation line. Blue, green, and red indicate the materials from the Spur group, the Early-maturation group, and the Color mutant group, respectively.

Epigenetic similarity calculation, clustering and principal component analysis

The results of the genetic similarity analysis based on the MSAP-MSL data sets (S6 Table) showed that the epigenetic similarity coefficient between varieties ranged from 0.515 to 0.807; the lowest similarity was found between 'Yishuizhongqiu' and 'Wengao No.3', and the highest similarity existed between 'Longfu' and 'Liquan spur'. The average epigenetic similarity between varieties in the 'Fuji' mutation line was 0.639, ranging from 0.577-0.678. The mean similarity coefficient between the standard 'Fuji' variety and its descendant varieties was 0.648, ranging from 0.561 (between 'Fuji' and 'Zhaoyuanflush red Fuji') to 0.758 (between 'Fuji' and 'Yanfu No.7'). The results showed that there was apparently epigenetic variation between mutant varieties and their 'Fuji' mother, and different degrees of epigenetic differentiation also occurred between different mutant varieties.

The UPGMA results showed (Fig 3(A)) that nearly 84% (43/51) of the varieties in the Color mutant group clustered closely together to form an independent cluster (Cluster 1). The Early-maturation mutant group, the Spur-maturation mutant group,

‘Fuji’, and the remaining eight varieties of the Color mutant group formed another group, designated Cluster 2. In comparison with the position of ‘Fuji’, other varieties showed obvious epigenetic variation, and the epigenetic variation of the Color mutant group was most prominent. We also found that within Cluster 2, the Early-maturation mutant group and Spur mutant group were mixed and distributed without distinct grouping, reflecting the close epigenetic relationship between them. In addition, although ‘Chang Fujia’, ‘Zhaofuwang’, ‘Yishuihong’, and ‘Jihong’ in the Color mutant group were concentrated in Cluster 2, they were relatively closer to Cluster 1, but ‘Aki-fu No.5’, ‘Line Ⅰ Fuji’, ‘Iwate line Ⅰ’, and ‘Iwafu No.10’ were located far from Cluster 1. These four varieties were all selected in Japan and belonged to the Color mutant group. Moreover, they were the earliest mutant cultivars obtained from ‘Fuji’, and few clonal descendants are selected from these cultivars at present. Therefore, their clustering results might be related to their original region of selection or breeding generation. In conclusion, the UPGMA results implied that the greatest epigenetic variation existed in the Color mutant group, which might present unique epigenetic and evolutionary mechanisms compared with the other two variant groups.

Fig 3. Dendrogram of 92 varieties obtained from UPGMA cluster analysis based on MSAP-MSL (A) and AFLP (B) data and the distribution of 16 methylation parameters of V5-V15 in the heat map between samples. Blue, green, and red in the circle of interest indicate the materials from the Spur group, the Early-maturation group, and the Color group, respectively.

The results of principal component analysis further supported the UPGMA clustering results. Principal components PC1 and PC2 accounted for 13.6% and 4.5% of the total variation, respectively. As shown in Fig 4 (A), the Spur mutant and Early-maturation groups were mixed, showing similar epigenetic consistency. ‘Fuji’ was included in this group, displaying a close epigenetic relationship with its members. The Color mutant group was basically independent and was located far from ‘Fuji’ and the other two mutant groups, indicating its unique epigenetic variation.

Fig 4. Principal coordinate analysis (PCA) of 92 varieties based on MSAP-MSL (A) and AFLP (B). Blue, green, and red indicate the materials from the Spur group, the Early-maturation group, and the Color group, respectively.

Genetic similarity calculation, clustering and principal component analysis

Genetic similarities of 0.653 (between ‘Miyazaki’ and ‘Su Fuji’) to 0.899 (between ‘Nagafu No.12’ and ‘Nagafu No.2’) were detected by NTSYS in the ‘Fuji’ mutation line, with an average similarity of 0.805 (S7 Table). The mean similarity coefficient between the standard ‘Fuji’ variety and its mutants was 0.80, ranging from 0.684 (between ‘Fuji’ and ‘Su Fuji’) to 0.877 (between ‘Fuji’ and ‘Yanfu No.7’), indicating that the genetic variation of the mutants differed from that of ‘Fuji’’.

The UPGMA results showed (Fig 3(B)) that 92 samples could be clustered into four groups (Clusters 1-4), and ‘Aki-fu No.5’ was clustered in the outermost area and separated into different groups, indicating distant genetic similarity. In addition,

‘Hongwangjiang’, ‘Gai Fuji’, and ‘Su Fuji’ were grouped together to form Cluster 3. Cluster 1 and Cluster 2 were closely related to each other. ‘Fuji’ was clustered in Cluster 1, which was closely related to ‘Shanfu No.6’, ‘Qiufuhong’, ‘Lele Fuji’, and ‘Qingnonghe No.1’. In Cluster 1 and Cluster 2, local clustering and accumulation phenomena of the same ‘Fuji’ mutation type were found, as observed in the regions between ‘Yanfu No.7’ and ‘Duanzhi Fuji’ (Spur mutant group), ‘Aki Fuji’ and ‘Nagafu No.36’ (Color mutant group), and ‘Miyazaki’ and ‘Chengji No.1’ (Spur mutant group) in the former group and the region between ‘Qianxuan No.1’ and ‘Shoufu No.3’ and that between ‘Yanfu No.3’ and ‘Nagafu No.4’ in the latter group. However, from the overall perspective of the cluster diagram, the three major ‘Fuji’ mutant groups were mixed and arranged, and there was no obvious boundary between different groups. Fig 4 (B) showed the PCA results of the 92 test varieties. The three dimensions of PC1 and PC2, PC1 and PC3, and PC2 and PC3 all showed results that were consistent with UPGMA clustering; that is, the genetic similarity among the three major mutation types of ‘Fuji’ was very high, without obvious boundaries.

Epigenetic and genetic diversity and molecular variation analysis

As shown in Table 4, the mean Shannon index, the number of effective alleles, and the mean expected heterozygosity based on MSL data among the three mutant groups were 0.415, 1.424, and 0.266, respectively. In general, the three genetic diversity indexes showed a consistent variation trend. For mutants with a high Shannon index (I), the effective allelic variance (Ne) and mean expected heterozygosity (He) were also higher.

The I and He indices presented significant differences among the three mutant groups (P<0.01). The analysis of molecular variance showed that most of the variation occurred within the mutant groups (85%), and only a very small proportion (15%) occurred among the mutant groups (P< 0.01).

Table 4. Epigenetic and genetic similarity and diversity in three different mutant groups of the 'Fuji' line.

Molecular marker type	Group	Percentage of Polymorphic Loci (%)	Epigenetic/Genetic diversity parameter		
			I	Ne	He
**MSAP**	Spur	82.895	0.388A	1.404A	0.250A
	Early-maturation	92.677	0.413B	1.428AB	0.266B
	Color	99.542	0.443C	1.440B	0.282C
	Mean	91.705	0.415	1.424	0.266
**AFLP**	Spur	60.516	0.284a	1.300a	0.183a
	Early-maturation	70.029	0.289a	1.307a	0.186a
	Color	80.573	0.296a	1.303a	0.188a
	Mean	70.372	0.290	1.303	0.186

Abbreviations: Ne, No. of effective alleles; I, Shannon index; He, Expected heterozygosity. Lower case letters indicate a significant difference at the 0.05 level. Lowercase letters indicate a significant difference at the 0.01 level.

The average Shannon index, number of effective alleles and average expected degree of heterozygosity in the three 'Fuji' mutant groups reflected by AFLP analysis were

0.290, 1.303 and 0.186, respectively. In general, there was no significant difference in genetic diversity indexes between the three groups basing on AFLP data. AMOVA showed that most of the genetic variation existed within the mutant groups (95%); a very small portion (5%) existed between the populations (*P*< 0.01).

Epigenetic and genetic structure analysis

Structure analysis was carried out based on MSL epigenetic data and AFLP-based genetic data, and 10,000 simulations were run with population numbers K=2 to 4. The results showed that LnP (D) values were generated for K=3 for both types of data, and no values were generated for K= 2 or K= 4. Therefore, K=3 was selected as the optimal number of clusters. Fig 5(A) showed that in the 'Fuji' mutation line, the Spur mutant group and the Early-maturation mutant group were highly consistent, while the Color mutant group showed a large proportion of heteromorphic lineages, indicating the unique epigenetic structure of the Color mutant group. As shown in Fig 5(B), there was no clear division between any of the mutant groups, which reflected the similarity of their genetic compositions.

Fig 5. Population structure based on MSAP-MSL (A) and AFLP (B) analysis for 'Fuji' and its mutants.

Outlier analysis

Bayesian population genomic analysis showed that out of 1748 methylation-sensitive MSAP loci, 601 exhibited positive alpha values, and 1147 exhibited negative alpha values. A positive alpha value indicates positive or diversified selection, while negative

values indicate the negative or purifying selection, also known as neutral selection. Thus, purifying selection seemed to play a major role in the differentiation of the different groups of ‘Fuji’ mutants (65.618%). However, epigenetic diversity selection still occurred at nearly 35% of loci. Sixteen epigenetic outlier loci were detected, accounting for 0.915% of the total DNA methylation-sensitive loci (Fig 6(A)). The epigenetic genetic frequencies of each locus in each mutant group were calculated individually. If the frequency of a locus in a group was greater than 50%, it was set as a specific outlier locus of that population. Therefore, Fig 7 showed that there were 13, 13, and 3 methylated outlier loci in the Spur, Early-maturation, and Color mutant groups, respectively. According to the profile of the frequency of loci, the Spur and Early-maturation mutant groups presented the same pattern, which basically maintained the state of the existence of the majority of loci in ‘Fuji’. However, the pattern of the Color mutant group was completely different from that of the other two mutant groups as well as ‘Fuji’. In the Color mutant group, 13 locus deletions and 3 new locus mutations occurred, indicating different epigenetic patterns of the outlier loci among the different mutant groups and suggesting unique epigenetic characteristics of the Color mutant group.

Fig 6. Bayesian genomic scans of the three mutant groups in the ‘Fuji’ line to identify outlier loci based on MSAP-MSL (A) and AFLP data (B).

Fig 7. The frequency of 16 outlier loci in 3 different groups of the ‘Fuji’ mutation line based on the MSAP-

MSL assay. Blue, green, and red indicate the materials from the Spur group, the Early-maturation group, and the Color group, respectively.

According to AFLP analysis, among the 1745 amplified loci, Bayesian population genomic analysis generated 568 loci with positive alpha values and 1177 loci with negative values. Purifying selection played a major role (67.450%) in the differentiation of the different 'Fuji' mutant groups. Genetic diversity selection occurred at only a few loci (32.550%). At the level of FDR=0.05, the number of outlier loci detected was 0, indicating that there was no significant difference in genetic differentiation between the three 'Fuji' mutant groups (Fig 6(B)).

Mantel correlation test of epigenetic and genetic similarity between different mutant groups

The Mantel test was conducted for the genetic and epigenetic similarity coefficient matrix obtained based on MSAP and AFLP analysis. The results showed that the r values of the genetic and epigenetic correlation coefficients of the Spur mutant group, Early-maturation mutant group, and Color mutant groups were -0.044 (P=0.368), 0.327 (P=0.978), and 0.107 (P=0.924), respectively. The overall correlation coefficient was 0.108 (P=0.987). The above results showed that there was a slight correlation between genetic and epigenetic variation in the 'Fuji' mutation lines, suggesting that the epigenetic variation was independent of genetic variation.

Comparison of epigenetic and genetic diversity between different mutant groups

The average polymorphic loci ratio, the polymorphic loci ratio, as well as epigenetic/genetic diversity parameters of each ‘Fuji’ mutant group based on MSAP were significantly higher than those obtained based on AFLP analysis (P<0.01), indicating that the epigenetic diversity of each mutant group in the ‘Fuji’ mutation line was significantly higher than the genetic diversity. For AMOVA between as well as within mutant group, a certain degree of differentiation under both markers was revealed, and among groups, the epigenetic differentiation ratio (15%) was greater than genetic differentiation ratio (5%).

Comparison of epigenetic and genetic structure between different mutant groups

The results of the UPGMA clustering analysis, PCA, and structure analysis based on AFLP and MSAP data showed that the three ‘Fuji’ mutant groups were genetically similar and that, regardless of what algorithm the analysis was based on, they could not be clearly separated. In terms of epigenetics, there was a close relationship between the Spur and the Early-maturation mutant groups, which were always mixed, similar to their genetic relationship. However, for the varieties of the Color mutant group tended to cluster together, and this group was independent of the other two mutant groups, showing its unique epigenetic structure.

Comparison of outlier loci between different mutant groups

The numbers of loci with positive and negative alpha values were approximately the same when generated on the basis of AFLP and MSAP. For each marker type, the proportion of loci with a negative alpha value was greater than that with a positive alpha

value. Regarding outlier loci, MSAP analysis detected 16 loci, whereas AFLP analysis detected 0, indicating that epigenetics played a large role in the differentiation of the 'Fuji' mutant groups; and from the viewpoint of the frequency of outlier loci between different groups, epigenetics in the Color mutant group contributed greatly to the differentiation of the 'Fuji' mutant groups.

Discussion

Extensive epigenetic research has been carried out in many plant species, whereas study on the fruit crops is still in progress. So far, the available reports mainly consist of several popular fruit crops such as apple [39-48], orange [49-50], banana [51], pear [52-53], strawberry [54], and grape [55-56]. Epigenetic studies in bud mutations are just in the initial stages since limited literature is available [40-41, 45-47, 52-53]. In 'Fuji' apple bud mutation occurrence, epigenetic mechanisms mediated by DNA methylation begun only last year [46-48], and all of them were done from the perspective of local DNA methylation. Few studies were performed on global DNA methylation in bud variation with a large sample size even on fruit crops. In the present study, we have incorporated a large-scale collection of 'Fuji' mutants from around the world as plant materials. Moreover, investigations of the genetic and epigenetic perspectives, at both the individual variety and mutant groups were conducted for the first time. Our studies revealed wide DNA methylation alterations between bud mutations in the 'Fuji' line. The results would be helpful for comprehensively understanding the epigenetic characteristics and differentiation of the 'Fuji' line. It also benefits for future further research on the epigenetic mechanism of bud mutations.

From an adaptive perspective, the modification of methylation status may allow trees to rapidly respond to abrupt changes in environmental conditions and contribute to their long-term responses to more general environmental scenarios [57]. Bud mutations often occur under stresses such as drought, pathogen infection, extreme weather and climate conditions, limited nutrient availability, human activities, and natural or artificial selection and are a result of adaptation to abnormal external environmental conditions [58, 59]. The contribution of epigenetic modification to the ability of plants to adapt to various stresses has been well demonstrated [26, 22]. Therefore, it can be theoretically deduced that a certain mutation is associated with epigenetic variation to some extent. This study detected nearly 32% epigenetic differentiation between the mutants and their original mother ‘Fuji’, showing many types of methylation pattern variants, and indicating that the variation in DNA methylation might be involved in the occurrence of ‘Fuji’ mutations. Our results explain the differentiation of epi-phenotypes, related to the similar genome structure, highlighted by Guarino et al. [60]. This may also verify the hypothesis of significant plant promotion to environmental adaptation, by phenotype diversification, increasing the probability of the plant to thrive when faced with a changing environment [3].

Different levels and degrees of methylation occur in higher plants to maintain normal plant development [59]. In general, the total genome DNA methylation level in different species detected by MSAP analysis is between 4.7% and 60.0% [61]. In this research, the DNA methylation level detected by MSAP was approximately 36%, similar to the findings of Li [62] in apple, in accordance with the relatively stable characteristics of

DNA methylation levels in this species.

CG and CHG are two main forms of DNA methylation at CCGG sites. Their relative proportions in the organism vary among different species, and the majority of available reports present results show that the proportion of the former is greater than of the latter [11]. In this study, the levels of CG and CHG methylation were found to be 22.311% and 13.599%, respectively. The level of the former was significantly higher than that of the latter, indicating full methylation derived was the main form of DNA methylation in the ‘Fuji’ line, which conformed to the results in the majority of plant species.

Natural mutations in plants are usually accompanied by changes in the levels of genetic and epigenetic. AFLPs and MSAPs are the two marker techniques commonly used for the detection of plant genetic and epigenetic variation [13-14]. Expected heterozygosity (He), also known as gene diversity, is an important parameter for the measurement of the general genetic diversity among plant groups and shows wide applicability to any system of polyploidy, self-crossing or asexual reproduction in populations [64]. The lower the genetic diversity, the higher the degree of the genetic homogeneity in a population. It is generally known that a heterozygosity value higher than 0.5 indicates genetic diversity between population individuals [65]. In this study, the average genetic diversity values of the two types of molecular markers were 0.186 (AFLP) and 0.266 (MSAP), suggesting that the genetic and epigenetic diversity levels of the mutant groups in the ‘Fuji’ line were relatively low. Comparatively, in the three examined mutant groups, the epigenetic expected heterozygosity of the Color mutant group was higher than those of the other two groups (P<0.01), implying its relatively

high epigenetic diversity. Additionally, in combination with the results from UPGMA cluster, STRUCTURE analysis, and outlier loci detection, the Color mutant group displayed unique epigenetic characteristics, which contributed largely to the epigenetic differentiation between the three mutant groups in the ‘Fuji’ line. This phenomenon might be related to the degree of difficulty in bud mutation of certain traits, the breeding and selection objectives in a given period, or the degree of artificial intervention.

Epigenetic variations may exhibit the phenotypic differences in different environments or growth periods and even in different tissues and plant organs [61]. However, in some species such as walnut, the global genomic DNA methylation level in different tissues and organs does not differ significantly [65]. The leaf can provide information about epigenetic modification and adaptation in response to different environmental conditions [60]. In our study, the leaves were considered as the plant material, as such, the results and conclusions of the epigenetic investigation were limited to the analysis of DNA matrix purified from particular tissue collection. The differences between the Early-maturation mutant group and the Color mutant group were mainly reflected in the performance of fruits. Therefore, if fruit samples of these two mutant types were used as the examined material, the results would not be similar to those obtained for leaves and remain inconclusive since no similar research has previously been conducted in apple. The tissue specificity of epigenetic modifications is also unknown.

Additionally, we found that the mean hypermethylation and demethylation frequencies of CHG type were both significantly higher than those of CG type,

indicating that variation in CHG methylation pattern played a key role in DNA methylation pattern variation in the ‘Fuji’ lines. In plants, cytosine methylation is a context-dependent process [9]. Our findings supported it. DNA methylation status reflects the outcome of the dynamic regulation of establishment, maintenance, and active-removal activities. CG and CHG methylation are catalyzed by various different enzymes and are under control by different pathways [9]. Hence, in view of our findings, the mechanism of epigenetic involvement in the occurrence of Fuji bud mutation could be further explored, focusing on the act of enzymes and detailed pathways related to CHG methylation.

In conclusion, the present study uncovered abundant changes in methylation levels and patterns between not only bud mutants and their mother ‘Fuji’ but also bud mutants, indicating that it may be possible that epigenetics mediated by DNA methylation was involved in the occurrence of the ‘Fuji’ bud mutation line. The epigenetic mechanism of the Color bud mutant group was unique, which could be the focus of further research. The correlation between epigenetic variation and genetic variation was weak, showing their independence, which provided further support for using ‘Fuji’ line as ideal sets for epigenetic studies in the future.

Acknowledgments

We are particularly grateful to Dr. Changfei Guan, Prof. Dorota Ewa Kruczyńska, and Dr. Sylwia Keller-Przybyłkowicz for English revision of the text.

Author Contributions

Conceived and designed the experiments: Xiaoyun Du**,** Laiqing Song. **Performed the experiments:** Xiaoyun Du**. Data analysis:** Xiaoyun Du, Yanbo Wang, Yan Tang. **Supervision**: Minxiao Liu, Xueqing Liu. **Funding acquisition:** Zhongwu Jiang. **Writing-original draft:** Xiaoyun Du. **Writing-review:** Lingling Zhao, Yanxia Sun, Xueyong Zhang, Daliang Liu.

References

1. Shu HR. Apple. Beijing: China Agriculture Press; 1999. Chinese.

2. Eccleston A, DeWitt N, Gunter C, Marte B, Nath D. Epigenetics. Nature. 2007; 7143: 395.

3. Balao F, Paun O, Alonso C. Uncovering the contribution of epigenetics to plant phenotypic variation in Mediterranean ecosystems. Plant biol. 2018; 20 (Suppl.1): 38-49.

4. Vogt G. Facilitation of environmental adaptation and evolution by epigenetic phenotype variation: insights from clonal, invasive, polyploid, and domesticated animals. Environ Epigenet, 2017; 3.

5. Law JA, Jacobsen SE. Establishing, maintaining and modifying DNA methylation patterns in plants and animals. Nat Rev Genet. 2010; 11, 204-220.

6. Finnegan EJ, Peacock WJ, Dennis ES. DNA methylation, a key regulator of plant development and other processes. Curr Opin Genet Dev. 2000; 10: 217-223.

7. Sahu PP, Pandey G, Sharma N, Puranik S, Muthamilarasan M, Prasad M. Epigenetic mechanisms of plant stress responses and adaptation. Plant Cell Rep. 2013; 32: 1151-1159.

8. Kapoor A, Agius F, Zhu JK. Preventing transcriptional gene silencing by active DNA demethylation. FEBS Lett. 2005; 579: 5889-5898.

9. Zhang H, Lang Z, Zhu JK. Dynamics and function of DNA methylation in plants. Nat Rev Mol

Cell Biol, 2018, 19: 489-506.

10. Niederhuth CE, Bewick AJ, Ji L, Alabady MS, Kim KD, Li Q, et al. Widespread natural variation of DNA methylation within angiosperms. Genome Biol. 2016; 17: 194.

11. Niederhuth CE, Bewick AJ, Ji L, Alabady MS, Kim KD, Li Q, et al. Epigenetic mechanisms of plant stress responses and adaptation. Plant Cell Rep. 2013; 32: 1151-1159.

12. Baránek M, Čechová J, Kovacs T, Eichmeier A, Wang S, Raddová J, et al. Use of Combined MSAP and NGS Techniques to Identify Differentially Methylated Regions in Somaclones: A Case Study of Two Stable Somatic Wheat Mutants. PLoS ONE. 2016; 11(10): e0165749.

13. Chwialkowska K, Korotko U, Kosinska J, Szarejko I, Kwasniewski M. Methylation sensitive amplification polymorphism sequencing (MSAP-Seq)-a method for high-throughput analysis of differentially methylated CCGG Sites in plants with large genomes. Front Plant Sci. 2017; 8: 2056.

14. Guarino F, Cicatelli A, Brundu G, Improta G, Triassi M, Castiglione S. The use of MSAP reveals epigenetic diversity of the invasive clonal populations of *Arundo donax* L.. PLoS ONE. 2019; 14(4): e0215096.

15. Reyna-lópes GE, Simpson J, Ruiz-herrera J. Differences in DNA methylation patterns are detectable during the dimorphic transition of fungi by amplification of restriction polymorphisms. Mol Gen Genet. 1997; 253: 703-710.

16. McClelland M, Nelson M, Raschke E. Effect of site-specific modification on restriction endonucleases and DNA modification methyltransferases. Nucleic Acids Research. 1994; 22: 3640-3659.

17. Xiong LZ, Xu CG, Maroof MAS, Zhang Q. Patterns of cytosine methylation in an elite hybrid

and its parental lines, detected by a methylation-sensitive amplification polymorphism technique. Mol Genet Genomics. 1999; 261: 439-446.

18. Lamo K, Bhat DJ, Kour K, Solanki SPS. Mutation studies in fruit crops: a review. Int J Curr Microbiol Appl Sci. 2017; 6 (12): 3620-3633.

19. Foster TM, Aranzana MJ. Attention sports fans! The far-reaching contributions of bud sport mutants to horticulture and plant biology. Hortic Res. 2018; 5: 44.

20. Shamel AD, Pomeroy CS. Bud mutations in horticultural crops. J Hered. 1936; 27: 487-494.

21. Okie WR. Handbook of Peach and Nectarine Varieties: Performance in the Southeastern United States and Index of Names. Springfield VA, 1998, Washington, DC.

22. Mozgova I, Mikulski P, Pecinka A, Farrona S. Epigenetic mechanisms of abiotic stress response and memory in plants. Epigenetics in Plants of Agronomic Importance: Fundamentals and Applications. 2019; 1-64.

23. Zhang CF. The investigation of Fuji apple bud mutation varieties (lines) and research of S-SAP molecular marker identified technique. Northwest A & F University; 2013. Chinese.

24. Song LQ, Zhao LL, Liu MY, Tang Y, Sun YX, Li YJ. Analysis of main features and influencing factors of ‘Fuji’ apple sport selection. China Fruit Vegetable. 2016; 36 (2): 54-56. Chinese.

25. Vos P, Hogers R, Bleeker M, Reijans M, Van de Lee T, Hornes M, et al. AFLP: a new technique for DNA fingerprinting. Nucleic Acids Res. 1995; 23: 4407-4414.

26. Schulz B, Eckstein RL, Durka W. Scoring and analysis of methylation-sensitive amplification polymorphisms for epigenetic population studies. Mol Ecol Resour. 2013; 13(4): 642-653.

27. Pérez-Figueroa A. msap: a tool for the statistical analysis of methylation-sensitive amplified polymorphism data. Mol Ecol Resour. 2013; 13 (3): 522-527.

28. IBM Corp. Released 2013. IBM SPSS Statistics for Windows, Version 22.0. Armonk, NY: IBM Corp.29. Rohlf FJ. NTSYS-pc: Numerical taxonomy and multivariate analysis system. 2000; Version 2.1 Exeter Publications, New York, USA.

30. Chen CJ, Xia R, Chen H, He YH. TBtools, a Toolkit for Biologists integrating various HTS-data handling tools with a user-friendly interface. Doi: http://doi.org/10.1101/289660.

31. Jombart T, Devillard S, Dufour AB, Pontier D. Revealing cryptic spatial patterns in genetic variability by a new multivariate method. Heredity. 2008; 101: 92-103.

32. Peakall R, Smouse PE. GenAlEx 6.5: genetic analysis in Excel. Population genetic software for teaching and research-an update. Bioinformatics. 2012; 28: 2537-2539.

33. Pritchard JK, Wen W, Falush D. 2003. Documentation for STRUCTURE software: version 2.

34. Earl DA, VonHoldt BM. Structure Harvester: a website and program for visualizing STRUCTURE output and implementing the Evanno method. Conserv Genet Resour. 2012; 4 (2): 359-361.

35. Jakobsson M, Rosenberg NA. CLUMPP: a cluster matching and permutation program for dealing with label switching and multimodality in analysis of population structure. Bioinformatics. 2007; 23: 1801-1806.

36. Rosenberg NA. DISTRUCT: a program for the graphical display of population structure. Mol Ecol Notes. 2004; 4 (1): 137-138.

37. Foll M, Gaggiotti O. A genome-scan method to identify selected loci appropriate for both dominant and codominant markers: a Bayesian perspective. Genetics. 2008; 180 (2): 977-993.

38. Jeffreys H. Theory of probability. 1961. Oxford University Press, Oxford.

39. Telias A, Wang KL, Stevenson DE, Cooney JM, Hellens RP, Allan AC. Apple skin patterning is

associated with differential expression of *MYB10*. BMC Plant Biol. 2011; 11: 93.

40. Xu YT, Feng SQ, Jiao QQ, Liu CC, Zhang WW, Chen WY, et al. Comparison of MdMYB1 sequences and expression of anthocyanin biosynthetic and regulatory genes between *Malus domestica* Borkh. cultivar ‘Ralls’ and its blushed sport. Euphytica. 2012; 185: 157-170.

41. El-Sharkawy I, Dong L, Xu K. Transcriptome analysis of an apple (*Malus*×*domesitica*) yellow fruit somatic mutation identifies a gene network module highly associated with anthocyanin and epigenetic regulation. J Exp Biol. 2015; 66 (22): 7359-7376.

42. Bai S, Tuan PA, Saito T, Honda C, Hatsuyama Y, Ito A, et al. Epigenetic regulation of *MdMYB1* is associated with paper bagging-induced red pigmentation of apples. Planta. 2016; 244 (3): 573-586.

43. Daccord N, Celton JM, Linsmith G, Becker C, Choisne N, Schijlen E, et al. High-quality de novo assembly of the apple genome and methylome dynamics of early fruit development. Nat Gent. 2017; 49: 1099-1108.

44. Xu J, Zhou S, Gong X, Song Y, Van Nocker S, Ma F, et al. Single-base methylome analysis reveals dynamic epigenomic differences associated with water deficit in apple. Plant Biotechnol J. 2018; 16: 672-687.

45. Jiang SH, Sun QG, Chen M, Wang N, Xu HF, Fang HC, et al. Methylome and transcriptome analyses of apple fruit somatic mutations reveal the difference of red phenotype. BMC Genomics. 2019; 20:117.

46. Zhang L, Hu J, Han XL, Li JJ, Gao Y, Richards CM, et al. A high-quality apple genome assembly reveals the association of a retrotransposon and red fruit colour. Nat Commun. 2019; 10: 1494.

47. Jiang SH, Wang N, Chen M, Zhang R, Sun QG, Xu HF, et al. Methylation of *MdMYB1* locus

mediated by RdDM pathway regulates anthocyanin biosynthesis in apple. Plant Biotechnol J. 2020; 1-13.

48. Cho HJ, Kim GH, Choi C. Differential gene expression and epigenetic analyses between striped and blushed skinned sports of ‘Fuji’ apple. Sci Hortic-amsterdam, 2020; 261: 108944.

49. Xu J, Xu H, Liu Y, Wang X, Xu Q, Deng X. Genome-wide identification of sweet orange (Citrus sinensis) histone modification gene families and their expression analysis during the fruit development and fruit-blue mold infection process. Front Plant Sci. 2015; 6: 607.

50. Huang H, Liu R, Niu Q, Tang K, Zhang B, Zhang H, et al. Global increase in DNA methylation during orange fruit development and ripening. P Natl Acad Sci USA. 2019; 116: 1430-1436.

51. Noyer J L, Causse S, Tomekpe K, Bouet A, Baurens F C. A new image of plantain diversity assessed by SSR, AFLP and MSAP markers. Genetica. 2005; 124: 61-69.

52. Wang ZG, Meng D, Wang AD, Li TL, Jiang SL, Cong PH, et al. The methylation of *PcMYB10* promoter is associated with Green skinned sport in ‘Max Red Bartlett’ pear. Plant Physiol, 2013; 162: 885-896.

53. Qian M, Sun Y, Allan AC, Teng Y, Zhang D. The red sport of ‘Zaosu’ pear and its red-striped pigmentation pattern are associated with demethylation of the *PyMYB10* promoter. Phytochemistry. 2014; 107: 16-23.

54. Cheng JF, Niu QF, Zhang BF, Chen KS, Yang RH, Zhu JK, et al. Downregulation of RdDM during strawberry fruit ripening. Genome Biol. 2018; 19:212.

55. Guo DL, YuYH, Xi FF, Shi YY, Zhang GH. Histological and molecular characterization of grape early ripening bud mutant. Int J Genomics. 2016; 4: 1-7.

56. Schellenbaum P, Mohler V, Wenzel G, Walter B. Variation in DNA methylation patterns of

grapevine somaclones (*Vitis vinifera* L.). BMC Plant Biol. 2008; 8(1): 78.

57. Sáez-Laguna E, Guevara MÁ, Díaz LM, Sánchez-Gómez D, Collada C, Aranda I, et al. Epigenetic variability in the genetically uniform forest tree species *Pinus pinea* L. PLoS One. 2014; 9(8): e103145.

58. Jing Shixi works collection Editorial Board. Jing Shixi works collection. 2009; Liaoning University Press: p24-40.

59. Whalley K. Dynamic DNA methylation. Nat Rev Neurosci. 2007; 8: 323.

60. Guarino F, Cicatelli A, Brundu G, Heinze B, Castiglione S. Epigenetic diversity of clonal white Poplar (*Populus alba* L.) populations: could methylation support the success of vegetative reproduction strategy? PLoS ONE. 2015; 10:e0131480.

61. Bartels A, Han Q, Nair P, Stacey L, Gaynier H, Mosley M, et al. Dynamic DNA methylation in plant growth and development. Int J Mol Sci. 2018; 19: 2144.

62. Li LG. Enhancement and identification of ploidy germplasms in apple. Shenyang Agricultural University; 2008. Chinese.

63. Nei M. Analysis of gene diversity in subdivided populations. Proc Natl Acad Sci USA. 1973; 70: 3321-3323.

64. Sheriff O, Alemayehu K. Genetic diversity studies using microsatellite markers and their contribution in supporting sustainable sheep breeding programs: A review. Cogent Food and Agriculture. 2018; 4: 1459062.

65. Zhou BB. Modification of DNA methylation by grafting to impact on phenotype of walnut. Chinese Academy of Forestry; 2014. Chinese.

Supporting information:

S1 Fig. Variation patterns of 15 subclasses of DNA methylation patterns.

(TIF)

S2 Fig. Summary of the changes in the cytosine methylation pattern in the 'Fuji' mutation line compared with the standard 'Fuji' cultivar.

(TIF)

S1 Table. Adaptors and primer sequences used for preamplification in AFLP and MSAP analyses.

(DOCX)

S2 Table. Summary of MSAP amplification from 23 primer combinations.

(DOCX)

S3 Table. Summary of AFLP amplification in 92 materials.

(DOCX)

S4 Table. Variation of the four major types of DNA methylation patterns (CG hypermethylation, CHG hypermethylation, CG hypomethylation, and CHG hypomethylation) in the 'Fuji' mutants.

(DOCX)

S5 Table. Number and frequency of change in cytosine methylation pattern in the bud sports compared with the standard 'Fuji'.

(XLSX)

S6 Table. Genetic similarity based on MSAP-MSL profiles.

(XLSX)

S7 Table. Genetic similarity based on AFLP data profiles.

(XLSX)

Fig1

Click here to access/download;Figure;Fig1.tif

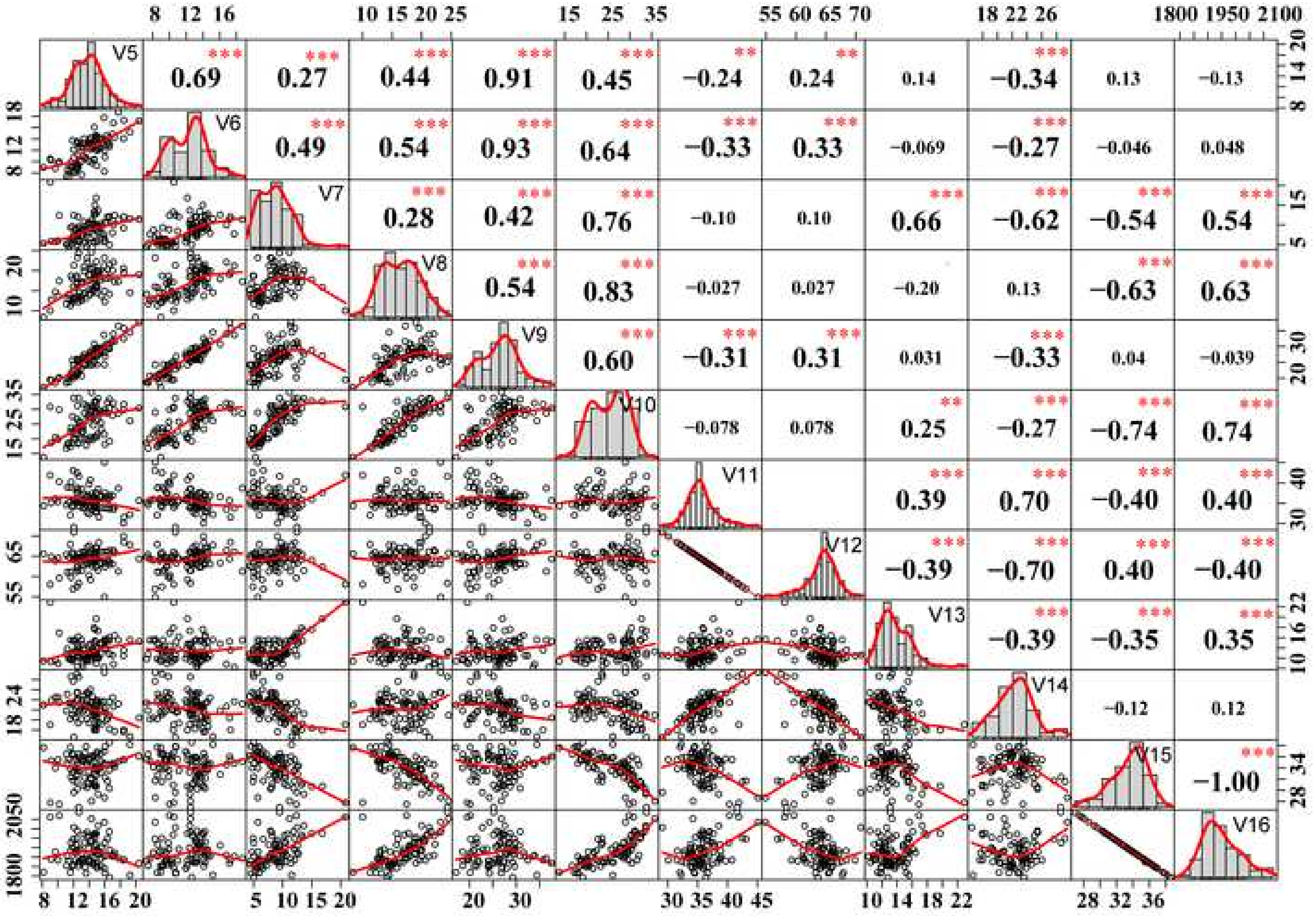

Figure

Click here to access/download;Figure;Fig2.tif

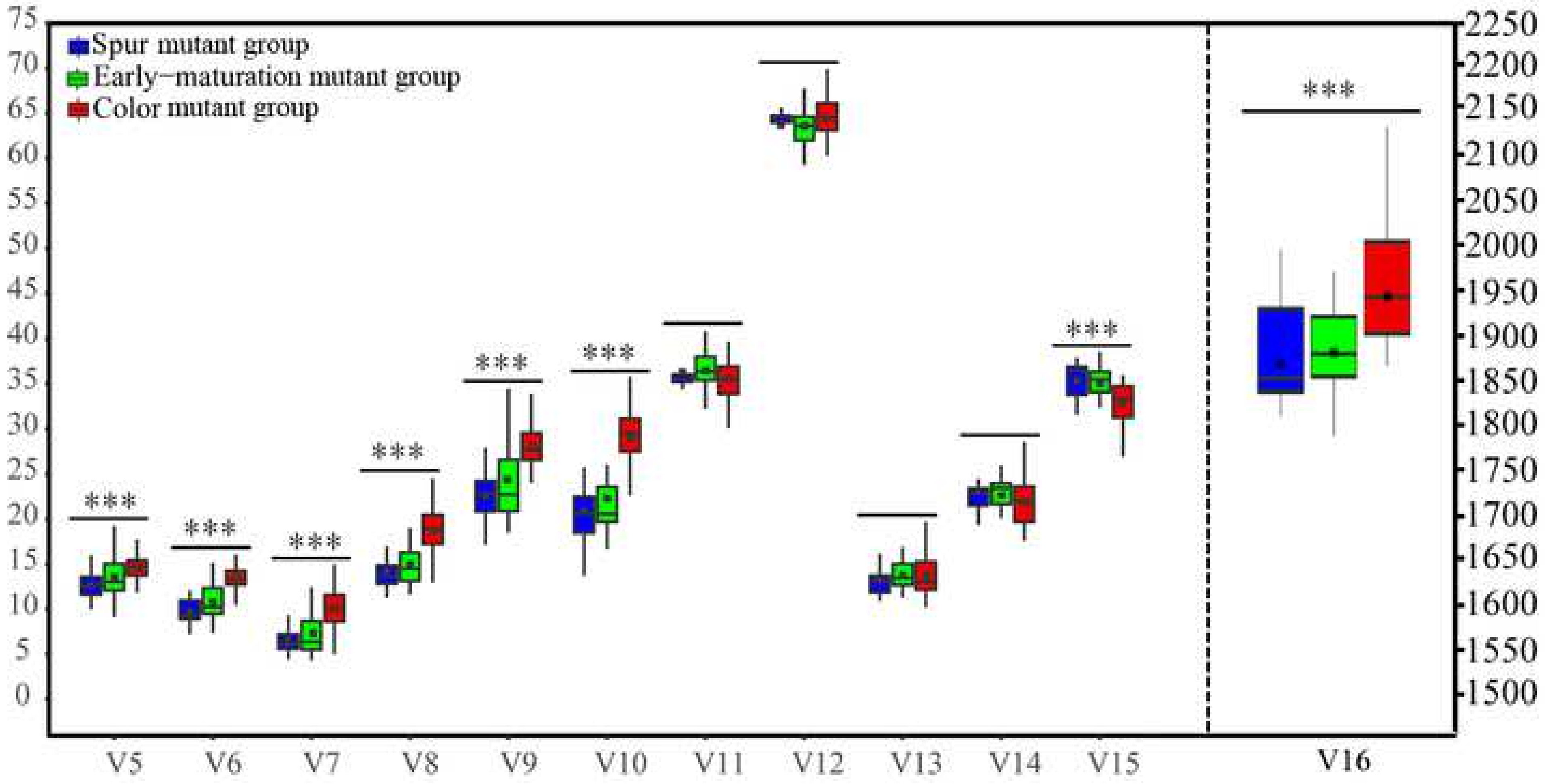

 Click here to access/download;Figure;Fig3.tif

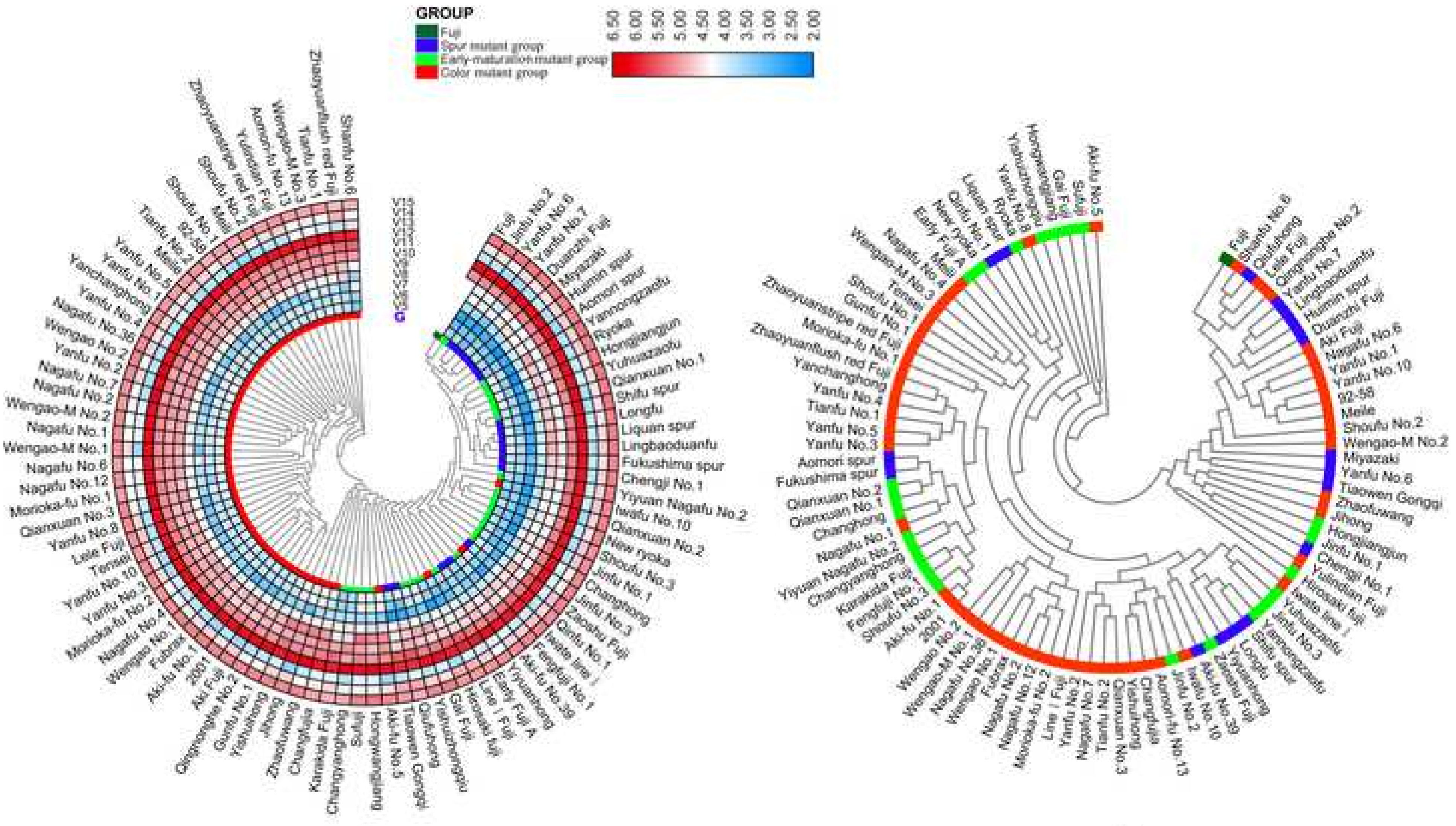

(A) (B)

Fig4 Click here to access/download;Figure;Fig4.tif

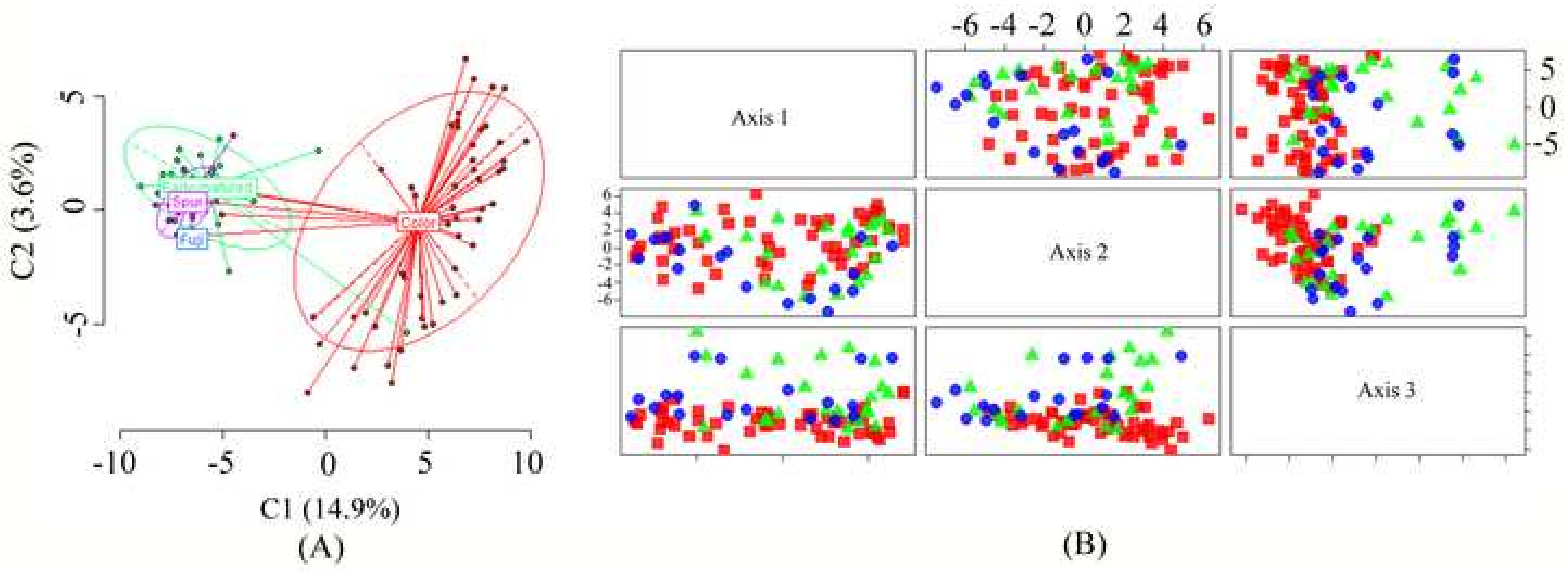

Figure

Click here to access/download;Figure;Fig5.tif

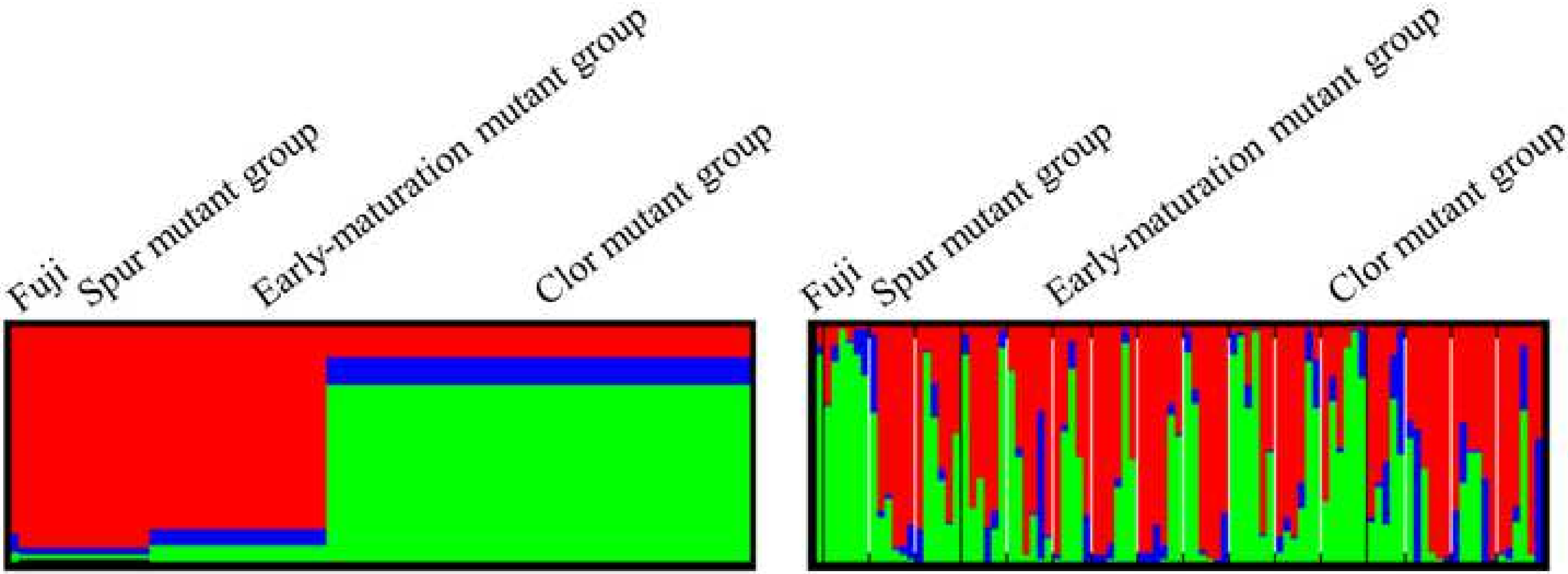

Fig6

Click here to access/download;Figure;Fig6.tif

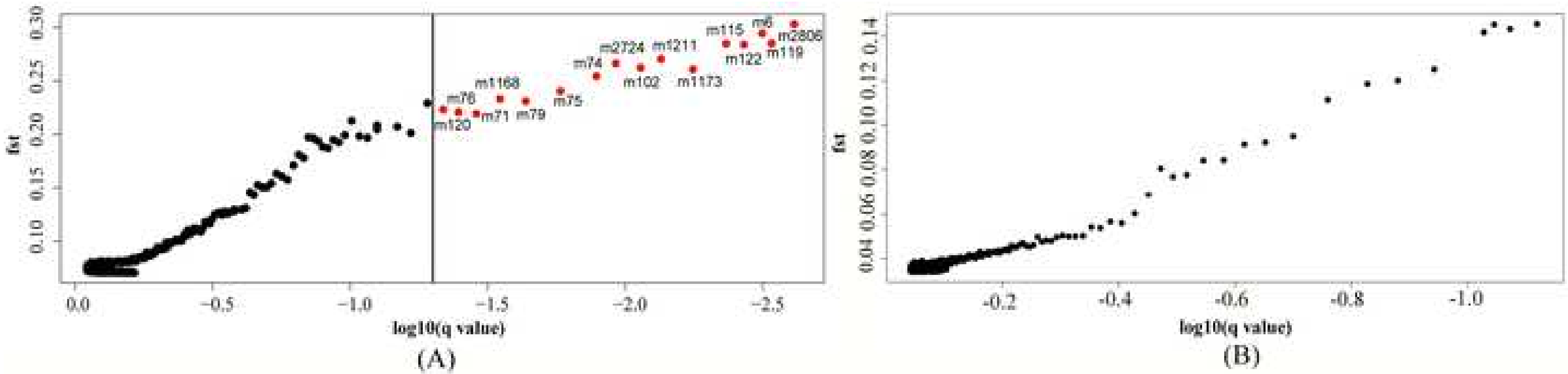

Figure Click here to access/download;Figure;Fig7.tif

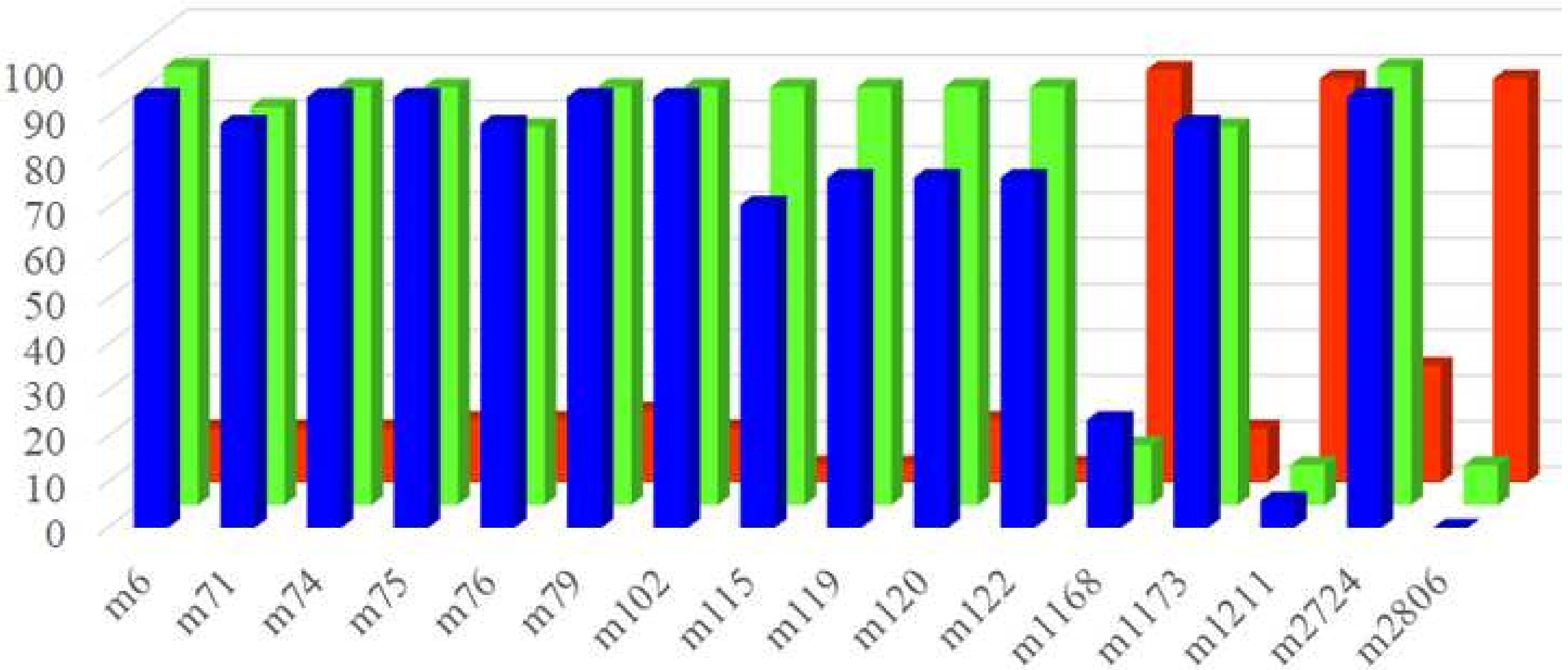

Click here to access/download
Supporting Information
S1_Table.docx

Click here to access/download
Supporting Information
S2_Table.docx

Click here to access/download
Supporting Information
S3_Table.docx

Click here to access/download
Supporting Information
S4_Table.docx

Click here to access/download
Supporting Information
S5_Table.xlsx

Click here to access/download
Supporting Information
S6_Table.xlsx

Click here to access/download
Supporting Information
S7_Table.xlsx

Click here to access/download
Supporting Information
S1Fig.tif

Click here to access/download
Supporting Information
S2Fig.tif

Click here to access/download
Other
AJE-Certification for edit.pdf

The assessment of epigenetic diversity, differentiation, and structure in the 'Fuji' mutation line implicates roles of epigenetic modification in the occurrence of different mutant groups as well as spontaneous mutants

Xiaoyun Du[1]¶, Yanbo Wang[1]¶, Minxiao Liu[1], Xueqing Liu[1], Zhongwu Jiang[1,2], Lingling Zhao[1,2], Yan Tang[1], Yanxia Sun[1], Xueyong Zhang[1], Daliang Liu[1], Laiqing Song[1]*

1 ~~Institution of Apple,~~ Yantai Academy of Agricultural Sciences, Yantai, Shandong, China
2 Yantai University, Yantai, Shandong, China

***** Corresponding author
E-mail: sdslq@126.com~~duxiaoyunduzi@163.com~~

¶These authors contributed equally to this work.

Abstract

~~*Malus domestica* Borkh. cv. 'Fuji' is one of the most economically important apple varieties in China and the world. 'Fuji' apple is prone to mutation, through which abundant bud mutants have been derived, the majority of which have been developed as superior varieties in production. Studies have shown that epigenetic are one of the important causes of bud mutations in fruit crops, but they have yet not to be systematically studied in a large sample size. The study of the characteristics of epigenetic effects in bud mutants is the premise for further studying the epigenetic mechanism of mutations and carrying out epigenetic breeding.~~ The ~~'~~Fuji~~'~~ line ~~exhibit~~includes many varieties~~, ~~ with a similar genetic background, and consistent inducement factors with epigenetic occurrence, thus it may be considered an ideal candidate for epigenetic research. ~~rich variation, a similar genetic background among different varieties, and the consistent inducement factors with epigenetic occurrence, so it can be considered an ideal candidate for epigenetic research.~~ In this study, 91 ~~excellent varieties~~bud mutations of 'Fuji' apple were used as the test materials~~, and u~~. Using the genetic variation within 'Fuji' as the control, the characteristics of epigenetic variation at different levels in both ~~individuals~~ varieties and ~~populations~~ mutant groups were examined. The results showed that: (1) the global genomic DNA methylation level of the 91 bud mutants of 'Fuji' ranged from 29.120%-45.084%, with an average of 35.910%~~, and i~~. Internal cytosine methylation was the main DNA methylation ~~way~~pattern. Regarding the variation of methylation patterns~~, during the occurrence~~ of 'Fuji' mutants, the vast majority of loci maintained the original methylation pattern ~~found~~ existed in 'Fuji'~~, and~~. CHG methylation variation was the main type of variation

Formatted: Font: 18 pt, Not Bold
Formatted: Font: Bold
Formatted: Font: Bold
Formatted: Font: Bold
Formatted: Not Superscript/ Subscript
Formatted: Not Superscript/ Subscript
Formatted: Font: 12 pt
Formatted: Font: 12 pt, Not Highlight
Formatted: Font: 12 pt
Formatted: Font: 12 pt, Not Highlight
Formatted: Font: 12 pt
Formatted: Font: 12 pt, Not Highlight
Formatted: Font: 12 pt

~~in the ‘Fuji’ mutants~~. (2) The variation in methylation patterns between the mutant groups was greater than that of methylation levels. Among these patterns, the variation in CHG methylation patterns (including CHG hypermethylation and CHG demethylation) was expected to be dominant~~ted~~. ~~The dominant type of epigenetic variation was different in different groups.~~ The observed variation in methylation levels was more important in the ~~color~~ Color mutant group; however, the variation in methylation patterns was more obvious in both the early maturation and ~~spur~~ Spur mutant groups. Moreover, the range of variation in the ~~early~~Early-maturation group was much wider than that in the ~~spur~~ Spur mutant group. (3) Epigenetic diversity and genetic diversity were both low between the mutant groups. In the ‘Fuji’ mutant groups, there was ~~a slight~~few correlation between genetic and epigenetic variation~~, and epigenetic differentiation was significantly higher than genetic differentiation; in addition,~~ , and epigenetic differentiation resulted in more loci with moderate or greater differentiation. (4) Purifying selection seemed to play a major role in the differentiation of different groups of ‘Fuji’ mutants (65.~~62~~618%), but epigenetic diversity selection still occurred at nearly 35% of loci. Sixteen epigenetic outlier loci were detected. ~~In conclusion, our data revealed that epigenetic mechanisms, and in particular DNA methylation, might be involved in the occurrence of bud mutations in the ‘Fuji’ line; the color mutant group presented a unique epigenetic mechanism compared with the spur and early-maturation mutant groups. Our findings are fundamental for understanding the significance of epigenetic variability arising from bud mutations and have significant implications for future studies on the epigenetic mechanism giving rise to color mutants.~~

Introduction

Malus domestica Borkh. cv. ‘Fuji’ is one of the most economically important apple varieties in China and the world. ‘Fuji’ apple is prone to mutation, through which abundant bud mutants have been derived, the majority of which have been adapted as superior varieties in production [1]. Studies have shown that epigenetics is an important cause of bud mutations in fruit crops, but it has not yet to be systematically studied in a large sample size. The study of the characteristics of epigenetic effects in bud mutants is the premise for further studying the epigenetic mechanism of mutations and carrying out epigenetic breeding.

Epigenetics studies the heritable changes in gene function that eventually lead to phenotypic variation with no changes in the underlying DNA sequence [2]. Epigenetics is involved in the regulation of gene expression, and changes are dynamic with respect to the endogenous and/or external environmental stimuli, thus affecting the phenotypic plasticity and environmental adaptability of organisms [3]. Epigenetic variation enhances biodiversity and complexity, especially in asexual organisms without gene recombination, which is helpful to the promotion of functional phenotypic diversity and has a greater impact on variation and evolution and more survival significance [4].

DNA methylation, representing the most important form of epigenetic modification, is ubiquitous in higher plants [5]. Different level of DNA methylation exists in normal organisms (5%-40%, [6]), while changes in DNA methylation can be caused by external environmental factors or various biotic and abiotic stresses [7]. Changes in DNA

Formatted: Font: 12 pt, Not Highlight

Formatted: Font: 12 pt

Formatted: Font: 12 pt

Formatted: Indent: First line: 0 ch

Formatted: Indent: First line: 1 ch

methylation level and methylation pattern may cause significant phenotypic variation in plant genome [8]. It can be expressed by the dynamics of hypermethylation and demethylation [9]. DNA methylation occurs on cytosines in different sequence contexts, and CG and CHG are the two main types of genome methylation. CG and CHG methylation are regulated by distinct enzymes and pathways [10]. Both CG and CHG methylation levels are different in a plant species, and CG methylation was reported predominately in many plant species [11].

The current research on DNA methylation is mainly focused on local and global aspects. The former mainly targets specific traits, starting with the changes directly related gene methylation in promoter or gene core sequence, and has more advantages in revealing the mechanism of occurrence of specific phenotypic variation; The latter studies the changes in the overall methylation of the genome, which is more conducive to the comprehensive analysis of the role of epigenetics in the variation and diversity. Many techniques have been developed to analyze global DNA methylation and its alterations [12]. The method of methylation-sensitive amplified polymorphism (MSAP) is one of the most efficient, economical and widely one used for the detection of DNA methylation events [13-14]. This approach is a modified version of the amplified fragment length polymorphism (AFLP) method based on the differential sensitivity of isoschizomeric restriction endonucleases to site-specific cytosine methylation [15]. MSAP analysis employs two types of cleaved enzymes *Eco*RI (rare cutter) and *Hpa*II/*Msp*I (frequent cutters) which recognize similar tetranucleotide 5'-CCGC representing a different sensitivity to methylation at the inner or outer cytosine. If one or both cytosines are methylated at both DNA strands, *Hpa*II is inactive. If one or both cytosines are methylated in only one strand they are cleaved by *Hpa*II. In contrast, *Msp*I reacts when only the internal cytosine is hemi- or fully- (double strand) methylated [16]. So, on the basis of variant band patterns resulting from differential digestion of the genome by *Hpa*II/*Msp* I isozymes, variation in the DNA methylation level and pattern in the whole genome is detected [15, 17]. MSAP has been successfully applied for the analysis of the variation in methylation levels and patterns in a variety of plant species [13].

In general, bud mutations are an important source of new varieties of fruit crops. Characteristics such as a perennial nature, long juvenile phase, heterozygosity, and sexual incompatibilities in fruit crops hamper their improvement through conventional breeding [18]. Compared with conventional hybridization, the selection of bud mutants gives the advantages of shortening the breeding cycle and reducing the workload and costs. Such an approach can be used to obtain excellent varieties by modifying varieties traits without changing the desirable qualities of the parent plant [19]. A variety of perennial fruit trees of economic importance originated from bud changes [20-21]. However, Spontaneous bud mutation occurs at a very low frequency, moreover, many of these mutations may be deleterious, making the organism less adapted to its environment, and in some cases may even be lethal [18]. Therefore, mutants that survive in adverse environments and even present excellent phenotypes are considered to have good characteristics for adaption. Epigenetic regulation mediated by DNA methylation is considered as one of the important mechanisms in plant adaptive

Formatted: Indent: First line: 1 ch

Formatted: Font: 12 pt, Italic

Formatted: Font: 12 pt

Formatted: Font: 12 pt, Italic

Formatted: Font: 12 pt

Formatted: Font: 12 pt, Italic

Formatted: Font: 12 pt

Formatted: Font: 12 pt, Italic

Formatted: Font: 12 pt

Formatted: Font: 12 pt, Italic

Formatted: Font: 12 pt

Formatted: Font: 12 pt, Italic

Formatted: Font: 12 pt

Formatted: Font: 12 pt, Italic

Formatted: Font: 12 pt

Formatted: Font: 12 pt, Italic

Formatted: Font: 12 pt

procedure [14, 22]. Studying the molecular mechanism of bud mutation at the DNA level is thus of great significance.

Fuji is the most representative apple cultivar in which plenty of new bud mutations arise [23]. Multiple bud mutants generated from the standard cultivar Fuji. Those represent highly similar genetic backgrounds and have abundant types of variation. Additionally, Fuji bud mutants were frequently reported to be available in orchards at unusual locations, such as under dense high-voltage lines, at high altitudes, subjected to an abnormal climate, severe drought, waterlogging, frost, or sudden diseases or insect pest infestations [24]. This means that the conditions for induction of bud mutation are consistent with the factors inducing epigenetic effects. Therefore, in a summary of the above mentioned, Fuji mutantion line could be regarded as ideal sets for research on epigenetic regulation. However, to our knowledge, global DNA methylation in this line has not been reported yet.

Formatted: Font: 12 pt

A general understanding of the mechanisms of genome-wide DNA methylation in 'Fuji' mutants is a prerequisite for their utilization in epigenetic mechanism studies or breeding. Therefore, in the present study, nearly one hundred 'Fuji' bud mutants were used as study materials for the first time. Then, through genetic variation as control, we focused on the analysis of variations in DNA methylation levels and patterns within groups and between groups as well as epigenetic diversity.

Formatted: Indent: First line: 1 ch

We were interested in the following questions: (1) is DNA methylation involved in the occurrence of bud mutations in the 'Fuji' line? (2) what are the characteristics of the changes in both DNA methylation levels and patterns? (3) what are the effects of DNA methylation on not only the clustering status of the 'Fuji' line at the variety level but also genetic structure and differentiation at the group level? (4) how is natural selection affecting the occurrence of different mutant groups in the Fuji line?

Formatted: Indent: First line: 1 ch

Formatted: Font: 12 pt

~~Epigenetics can explain the variation caused by changes in gene expression patterns; these modifications are unrelated to changes in genome sequences and can be stably inherited between generations [1]. DNA methylation is one of the most important forms of epigenetic modification and is ubiquitous in higher plants; this modification presents important biological functions in maintaining the structural stability of the organism's genome and regulating gene expression [2]. Different amounts and degrees of DNA methylation are observed in normal organisms (5%-40%) [3]. Extensive methylation status changes can be triggered in the genome by external factors, among which biological and abiotic stresses are important inducing factors [4]. When abnormal environmental stimuli or other factors are encountered, the DNA methylation mechanism can react rapidly as a potential initial defense system for the plant genome, and the transcriptional activity of protein-encoding genes will be changed by variations in DNA methylation levels and patterns [5]. Additionally, transposon activity is regulated even if the genomic structure is mutated to respond to environmental factors [6].~~

~~In the face of environmental stress, the epigenetic variations that arise in plants are not always unique and consistent. The final trend of the variation is influenced by differences in varieties, the environment and other factors, resulting in the epigenetic~~

diversity of species, which is thought to be the result of the joint effects of genetic diversity and environmental diversity [7, 8]. Epigenetic variation in turn promotes the occurrence of genetic variation, which is beneficial for plant adaptability to the environment and phenotypic diversity [9]. In recent years, analyses of epigenetic variation have been applied to population genetic studies to understand epigenetic variation at the population level, and a series of studies have suggested that epigenetic variance may be one of the core factors responding to ecology and evolution in natural populations [10]. DNA methylation status includes the methylation level and methylation pattern, and any change in status may cause significant phenotypic variation in plants [11]. Changes in DNA methylation patterns can be divided into hypermethylation and demethylation. Currently, it is widely accepted that DNA hypermethylation suppresses gene expression and that demethylation activates gene expression [12].

Many techniques have been developed to analyze global DNA methylation and its alterations, among which the method of methylation-sensitive amplified polymorphism (MSAP) is one of the most efficient, economical and widely used methods for detecting DNA methylation [13-14]. This approach is a modified version of the amplified fragment length polymorphism (AFLP) method based on the differential sensitivity of isoschizomeric restriction endonucleases to site-specific cytosine methylation [15]. On the basis of variant band patterns resulting from differential digestion of the genome by *Hpa* II/*Msp* I isozymes, which recognize the same restriction site (CCGG/GGCC) but present a difference in sensitivity to methylation at the inner or outer cytosine, variations in the DNA methylation level and pattern in the whole genome are detected [15-16]. According to the working principle of MSAP, for a particular 5’-CCGG-3’ site, four methylation states exist [17]: nonmethylation (condition I), external cytosine methylation (CHG methylation, condition II), internal cytosine methylation (CG methylation, condition III), and full methylation or gene mutation (condition IV). According to the method reported by Cervera et al. [18], the bands whose absence/presence differs between a pair of parallel *Eco*RI/*Msp*I and *Eco*RI/*Hpa*II digests for a given genotype are defined as methylation-sensitive polymorphisms (MSPs) and scored as ‘1’, and the bands are otherwise scored as ‘0’; for methylation-insensitive polymorphisms (MISPs), bands that are both absent or present in a pair of parallel *Eco*RI/*Msp*I and *Eco*RI/*Hpa*II profiles are scored as ‘0’ and ‘1’, respectively. MSAP has been successfully applied for the analysis of the variation in methylation levels and patterns in a variety of plants [13].

Bud mutations are an important source of new varieties of fruit crops. Characteristics such as a perennial nature, long juvenile phase, heterozygosity, and sexual incompatibilities in fruit crops limit their improvement through conventional breeding [19]. Compared with conventional hybridization breeding, the selection of bud mutants presents the advantages of a short cycle, small workload and low cost; this approach can be used to obtain excellent varieties by repairing individualvarieties traits without changing the desirable qualities of the parent plant [20]. Therefore, it is a simple and effective method for breeding. According to statistics, 10% of the existing elite apple varieties were selected from bud mutants, and 1/2 of the total production of apple

Formatted: Font color: Red

Formatted: Default, Justified, Indent: First line: 1.5 ch

Formatted: Font color: Red

in the world comes from the yield of bud mutants. ‘Fuji’ is the most representative apple variety in which new cultivars arise via bud mutation [21]. ‘Fuji’ is one of the most important cultivated apple varieties in the world. It is also the most commonly cultivated apple variety in China, accounting for 60% of the total production of apples. During the early stages of the release of this cultivar, bud mutant selection was carried out due to defects resulting in uneven fruit types and poor coloration, and color variation mutants were the dominant focus; however, this work revealed other bud mutant types, such as spur type and early maturation type mutants. More than 150 excellent ‘Fuji’ bud mutants have been obtained worldwide thus far [22]; moreover, there are significantly more color type variants than variants of the other two types. Extreme environments are usually responsible for spontaneous mutations in plants. In ‘Fuji’, bud mutants have been reported to be frequently available in orchards at special locations, such as under dense high voltage lines and at high altitudes, and those subjected to an abnormal climate, severe drought, waterlogging, low temperature frost, or sudden diseases or insect pest infestations [23].

Formatted: Font: 12 pt

The investigation of the mechanism of bud mutation is the premise of elucidating the mechanisms of important characteristics. Changes in gene sequences or transposon behavior are thought to be the main reasons that bud mutations occur [20]. Accumulated evidence implies that epigenetics also plays a role in the occurrence of bud mutations [24-25]. At present, the investigations on DNA methylation are generally divided into site methylation and global DNA methylation. In fruit crops, the methylation of a specific locus in the genome, such as the *Myb* promoter, have been performed [24-29]; however, the involvement and characteristic of global DNA methylation in bud mutant occurrence is poorly understood [30-31].

Spontaneous bud mutation occurs at a very low frequency of 1 in 10 lacs or 10^{-6}; moreover, many of these mutations may be deleterious, making the organism less adapted to its environment, and some may even be lethal [19]. Therefore, mutants that survive in adverse environments and even present excellent phenotypes are considered to present good characteristics for adaption. Epigenetic regulation mediated by DNA methylation is considered one of the important mechanisms of adaptive learning [14, 32]. Therefore, it is of great significance to study the molecular mechanism of bud mutation via the method of DNA methylation. The ‘Fuji’ line consists of a series of bud mutant varieties generated from the standard cultivar ‘Fuji’, in which different mutant individualvarietiess present highly similar genetic backgrounds and can be regarded as near isogenic lines (NILs) of field crops. Therefore, samples of these mutants were examined to explore the molecular mechanism of bud mutation with decreased interference from different genetic backgrounds. Additionally, since the occurrence of these natural variations is closely correlated with environmental factors, the ‘Fuji’ line is also an ideal material for research on epigenetic regulation and further characterization of the molecular mechanism of the excellent mutations found in this line. Epigenetic research on ‘Fuji’ apple is rarely reported at present. Moreover, in the available literatures, ‘Fuji’ was generally used as one or a small fraction of the test materials and analyzed together with other test materials. It is worth to be noted that there has been no specific research on the variation of the ‘Fuji’ line with a large sample

size. Therefore, in the present study, nearly one hundred elite ‘Fuji’ varieties were used as study materials for the first time. Then, through the comparison of genetic variation, we focused on the analysis of variations in DNA methylation levels and patterns within groups and between groups as well as epigenetic diversity. We asked the following questions: (1) is DNA methylation involved in the occurrence of bud mutations in the ‘Fuji’ line? (2) what are the characteristics of the changes in both DNA methylation levels and patterns not only between individualvarietiess but also between different mutant groups? (3) what are the effects of DNA methylation on the clustering status of the ‘Fuji’ line at the individualvarieties level and on genetic structure and differentiation at the group level? (4) how has natural selection affected the occurrence of different mutant groups in the ‘Fuji’ line?

Materials and Methods

Plant material and MSAP, AFLP analyses

This study was conducted on ‘Fuji’ and 92 it’s 91 elite varieties arising from bud mutations in ‘Fuji’ line (including ‘Fuji’) mainly bred in Japan or China, and among of which, 91 were bud mutations from the same mother cultivar ‘Fuji’. Majority of them have been developed into popular cultivars. . Three major types: cColoring mutation groupmutant group, earlyEarly-maturation mutation groupmutant group, and spur Spur mutation groupmutant group could be classified (Table 1). Fully expanded fresh leaves were collected from each examined individualvarieties. Plants We were sampled plant materials with the same age on the same dates (12 June 2016) and at the their identical developmental phenological stage (bearing fully expanded leaves)as well as the same phenological, trying to avoid that possible developmental variation in DNA methylation would confound individualvarieties variety or group differences in methylation patterns. Fully expanded fresh leaves from pooled varietiess (ca. 5 plants being clonally propagated from a single mother variety) of each variety were collected. Young leaves immediately frozen in liquid nitrogen and then stored at -80℃ prior to DNA isolation.

Total genomic DNA from leaf tissue wereas extracted by using DNeasy Plant Mini Kit (Qiagen) and the manufacturer’s protocol. MSAP molecular marker was used for the analysis of epigenetic variation analysesin plants examined. For comparison, genetic variation was also conducted by AFLP molecular marker was used for surveying genetic variation existed in plants examined. Both methods performed on the same set of plants., and Label *Eco*R I primers were labeled with or green (JOE), blue (FAM), or yellow (NED) fluorescent dyes.

MSAP and AFLP were performed as described by Xiong et al. [16][17] and Vos et al. [33][25], respectively. MSAP was essentially the same as the AFLP protocol. The difference between MSAP and AFLP procedure was replacing the *Mse*I enzyme with the enzyme either *Hpa*II or *Msp*I in MSAP. Thus, differences in the PCR products detected with *Eco*RI/*Hpa*II and *Eco*RI/*Msp*I would reflect different methylation states. The primer sequences of MSAP and AFLP were listed in the S1 Table. Based on previous pilot tests, we selected 23 optimal primer combinations for MSAP analysis (H/M-TCT+E-CT, H/M-TCT+E-CC, H/M-TCT+E-GC, H/M-TCC+E-AG, H/M-

Formatted: Font: 12 pt

Formatted: Font: (Default) URWPalladioL-Roma, 12 pt

Formatted: Font: 12 pt

Formatted: Font: (Default) URWPalladioL-Roma, 12 pt

Formatted: Font: 12 pt

Formatted: Font: (Default) URWPalladioL-Roma, 12 pt

Formatted: Font: 12 pt

Formatted: Font: (Default) URWPalladioL-Roma, 12 pt

Formatted: Font: (Default) URWPalladioL-Roma, 12 pt

Formatted: Font: (Default) URWPalladioL-Roma, 12 pt

Formatted: Font: 12 pt

Formatted: Font: (Default) URWPalladioL-Roma, 12 pt

Formatted: Font: 12 pt

Formatted: Font: (Default) URWPalladioL-Roma, 12 pt

Formatted: Font: 12 pt

Formatted: Font: (Default) URWPalladioL-Roma, 12 pt

Formatted: Font: 12 pt

Formatted: Font: (Default) URWPalladioL-Roma, 12 pt

Formatted: Font: 12 pt

Formatted: Pa9, Justified, Indent: First line: 0 ch, Adjust space between Latin and Asian text, Adjust space between Asian text and numbers

~~TTC+E-AG, H/M-TTC+E-CC, H/M-TTG+E-CA, H/M-TTG+E-CC, H/M-TTA+E-AG, H/M-TTA+E-CA, H/M-TTA+E-CT, H/M-TTA+E-GG, H/M-TGA+E-CT, H/M-TGA+E-CG, H/M-TGA+E-GG, H/M-TGT+E-GG, H/M-TAC+E-CA, H/M-TAC+E-CC, H/M-TAC+E-GC, H/M-TAC+E-GG, H/M-CTC+E-AC , H/M-CTC+E-CG, H/M-CCA+E-CG~~ S2 Table) and 19 optimal primer combinations for the AFLP analysis (~~E-AGG+M-CAA, E-AGG+M-CAC, E-AGG+M-CAG, E-AGG+M-CTG, E-AGG+M-CTA, E-AGG+M-CTT, E-AAG+M-CAC, E-AAG+M-CAG, E-ACC+M-CAA, E-ACC+M-CAC, E-ACC+M-CAG, E-ACC+M-CTG, E-ACG+M-CAA, E-ACG+M-CAC, E-ACG+M-CAG, E-ACG+M-CAT, E-ACG+M-CTG, E-ACG+M-CTA, E-ACG+M-CTT~~S3 Table). HM-, E-, and M- corresponded to the sequence of H/M00, E00, and M00, respectively (S1 Table). The repeatability of banding patterns assessed by conducting two sets of independent MSAP and AFLP analyses and only the consistent bands were included.

Using an automated sequencer (ABI PRISM®3730 Genetic Analyzer, Applied Biosystems) to separated and detected MSAP and AFLP PCR products. GeneScan Rox-500 labeled with a red (ROX) dye was an internal size marker. A peak size between 80 and 500 bp was selected to study the polymorphic DNA fragments. MSAP products were scored as present '1' or absent '0' on the chromatogram to create a binary matrix.

Statistical analysis

The resulting data of MSAP and AFLP were processed using Excel 2016. According to the scoring approach [26], MSAP raw data were transformed into a binary data matrix before running statistical analyses and computation. Because the enzymes used for MSAP analysis recognize the same restriction site (5'-CCGG) but have different sensitiv~~ity~~itiese to methylation modifications, the final products of MSAP present four types of DNA methylation at the 5'-CCGG sites, namely, no methylation (H1M1, condition–I), CHG methylation (H1M0, condition–II), CG methylation (H0M1, condition–III), and CG/CHG methylation (H0M0, conditionIV). DNA methylation level%=(condition-II+condition-III)/(condition-I+condition-II+condition-III+condition IV).

Methylation-Sensitive Polymorphism (MSP) matrix data, which were converted using R program *msap* [27], were used for all ~~analysis~~ analyses of DNA methylation. The change of patterns of cytosine methylation at CCGG sites in the 'Fuji' mutation line was listed in Table 2. Accordingly, contrasting to the standard cultivar 'Fuji', the changes of DNA methylation pattern in its series mutations could been summarized into four categories: (a) CG hyper: H1M1 to H0M1, H1M0 to H0M0, H1M0 to H0M1, H1M1 to H0M0; (b) CHG hyper: H1M1 to H1M0, H0M1 to H0M0, H1M1 to H0M0, H0M1 to H1M0; (c) CG hypo: H0M1 to H1M1, H0M0 to H1M0, H0M1 to H1M0; and (d) CHG hypo: H1M0 to H1M1, H0M0 to H0M1, H1M0 to H0M1, H0M0 to H1M1 ~~as described by Li et al. [34]~~.

~~Table 1. Plant materials and summary of MSAP amplification in the examined genotypes~~varieties~~.~~

DNA methylation analysis and variation coefficient

Formatted: Font: 12 pt

Formatted: Font: 12 pt

Formatted: Font: 12 pt

Formatted: Default, Justified, Indent: First line: 1 ch, Adjust space between Latin and Asian text, Adjust space between Asian text and numbers

Formatted: Font: 12 pt

Formatted: Font: 12 pt

Formatted: Font: 10.5 pt

calculation of different mutant groups in ‘Fuji’

The epigenetic relationship of different ~~mutation group~~mutant groups in ‘Fuji’ was analyzed ~~with~~ to 12 indexes related with DNA methylation levels and patterns. Indicators include~~:~~ CHG hyper-methylation frequency (V5), CG hyper-methylation frequency (V6), CG hypo-methylation frequency (V7), CHG hypo-methylation frequency (V8), the total hyper-methylation frequency (V9), the total hypo-methylation frequency (V10), total genome methylation frequency (V11), the frequency of condition I (Non-methylation frequency, V12), the frequency of condition II methylation (CHG methylation frequency, V13), the frequency of condition III methylation (CG methylation ratio, V14), the frequency of condition IV methylation (V15), and the total amplified loci of ~~genotypes~~varieties (V16).

Formatted: Font: 12 pt

The Coefficient of Variation (CV) of each of the above indexes was calculated in the three mutation groups in order to investigate whether ~~individual~~varieties in different ~~mutation group~~mutant groups behave in different ways. CV= (standard deviation SD/Mean)× 100%. Correlation analysis of 12 major epigenetic parameters (V5-V15) was performed by software IBM SPSS Statistics 22 [28].

Epigenetic and genetic similarity, clustering and principal component analysis

The similarity coefficient between varieties was calculated using the SM coefficient through the SimQual procedure of NTSYSpc 2.11 software package [~~35~~29] ~~was used to calculate the similarity coefficient between individual~~varietiess, and unweighted pair group method average (UPGMA) method was used for cluster analysis. Circle diagrams were drawn using TBtools [30]. MSAP-PCA and AFLP-PCA analysis were carried out by R program package *msap* ~~[36~~27~~]~~ and Adegenet 2.1.1 ~~[37]~~ [31], respectively.

Formatted: Font: 12 pt

Epigenetic and genetic diversity and molecular variation analysis

~~The software GenAlex 6.51 [38] was used to calculate v~~Various diversity measurement parameters, including polymorphism site proportion (PPL), effective allele variance (Ne), Shannon diversity index (I), ~~and~~ expected heterozygosity (He), hierarchical analysis of molecular variance (AMOVA) analysis between and within mutant groups were estimated in GenAlex 6.51 [32], using 999 random permutations.

Formatted: Font: 12 pt

Formatted: Font: 12 pt

Formatted: Font: 12 pt

Epigenetic and genetic ~~differentiation and structural~~ Structural analysis

~~Software GenAlex 6.51 was used for AMOVA analysis, locus-by-locus AMOVA analysis and Pairwise AMOVA analysis of all materials, then the genetic differentiation degree (PhiPT) was obtained. In epigenetic analysis, both AMOVA and correlation analysis of 12 major epigenetic parameters (V5-V15) were added and~~

Formatted: Pa9, Justified, Indent: First line: 0.47"

~~performed by software IBM SPSS Statistics 22 [39].~~

~~Using~~ STRUCTURE 2.3.4 ~~[40]~~ [33] ~~–~~ was utilized to ~~analysis~~ analyze genetic and epigenetic structure. Admixture and correlated allele frequencies model were chosen. Ten independent runs were made with values of K set from 2 to 4, with three iterations for each value of K. The length of the burn-in period was set at 10,000, and the number of Markov chain Monte Carlo (MCMC) repeats after burn-in was set at 100,000. The result from STRUCTURE output file ~~were~~ was performed on line by STRUCTRE Harvester 0.6.8 ~~[41]~~[34]. The results were averaged for a particular K using CLUMPP 1.1.2 ~~[42]~~ [35] and visualized by DISTRUCT ~~[43]~~ [36].

Potential outlier detection

BAYESCAN v2.1 ~~[44]~~ [37] was used to test Fst outliers in global and pairwise comparisons. A reversible-jump Markov chain Monte Carlo algorithm based on a Bayesian likelihood approach is used in BAYESCAN to estimate the ratio~~n~~ of posterior probabilities of selection over neutrality [the posterior odds (PO)]. Based on Jeffreys' ~~[45]~~[38] scale of evidence, a $\log_{10}PO > 2.0$ is interpreted as 'strong evidence' of selection. For our analysis, the estimation of model parameters was set as 10 pilot runs of 5,000 interaction each, followed by 100,000 interactions ~~[44]~~[37]. Outliers were calculated using a burn-in of 50,000 interactions, a thinning interval of 20, and a sample size of 5000. FDR=0.05 was used.

Correlation analysis between epigenetic diversity and genetic diversity

Using NTSYS2.0 software, the correlations between ~~Mantel tested~~ epigenetic distance and genetic distance of three 'Fuji' mutant groups as well as varieties calculated by MSAP and AFLP ~~was~~ were ~~performed~~ evaluated using the Mantel test implemented through NTSYSpc 2.11 software package [28]. ~~on the genetic distance and epigenetic distance calculated by MSAP and AFLP of three 'Fuji' mutant groups as well as individual~~varieties~~s, and the similarity coefficient value r was calculated~~.

All of the statistical significance in this study was determined by IBM SPSS Statistics 22 [32]. A value of P < 0.05 was considered significant.

Results

MSAP and AFLP amplification

A total of 2954 CCGG loci were detected via the genome-wide methylation analysis of 92 'Fuji' ~~genotypes~~varieties with 23 pairs of MSAP primer combinations, and 129 CCGG loci were amplified on average with each pair of primers. Among these loci, 2752 CCGG loci showed polymorphism, for a polymorphic ratio of 93.162%. Different MSAP amplification patterns were obtained from different ~~genotypes~~varieties (Table 1). The number of amplified methylated loci ranged from 1793 to 2136, and that of

Formatted: Font: 12 pt

Formatted: Font: 12 pt

Formatted: Font: 12 pt

Formatted: Justified

Formatted: Font: (Default) Times New Roman, 12 pt

Formatted: Font: 12 pt

Formatted: Font: (Default) Times New Roman, 12 pt

Formatted: Font: 12 pt

Formatted: Justified, Indent: First line: 1 ch

Formatted: Font: (Default) Times New Roman, 12 pt

Formatted: Font: 12 pt

Formatted: Font: (Default) Times New Roman, 12 pt

Formatted: Indent: First line: 2 ch

Formatted: Left, Don't adjust space between Latin and Asian text, Don't adjust space between Asian text and numbers

Formatted: Font: 12 pt

polymorphic loci ranged from 1613 to 1956, with an average polymorphic ratio of ~~64~~63.~~09~~566% (~~54~~58.~~92~~ 612 to ~~70~~71.~~88~~076%). Among the 2954 CCGG loci, 1748~~4~~ were methylation-sensitive loci (MSL), accounting for ~~60.39~~59.174% of the total amplified loci. A total of 1627 polymorphic MSL loci were obtained, accounting for 93.078% of the total amplified loci. ~~The number of non-methylation-sensitive (NML) loci was 1206, corresponding to a ratio of 67.60% in relation to the total amplified MSL loci~~ (S2 Table).

Table 1. Plant materials and summary of MSAP amplification in this study.

Variety Code	Name	Mutation type	Total loci	Non-methylated CCGG loci (%)	Methylated CCGG loci (%)		
					CG-methylated	CHG-methylated	Total
M1	Fuji	Origin	1841	1212(65.834)	396(21.510)	233(12.656)	629(34.166)
M2	Shanfu No.6	Color	2112	1227(58.097)	402(19.034)	483(22.869)	885(41.903)
M3	Iwate line Ⅰ	Color	1880	1134(60.319)	475(25.266)	271(14.415)	746(39.681)
M4	Iwafu No.10	Color	1904	1163(61.082)	478(25.105)	263(13.813)	741(38.918)
M5	Line Ⅰ Fuji	Color	1996	1136(56.914)	552(27.655)	308(15.431)	860(43.086)
M6	Gunfu No.1	Color	1875	1268(67.627)	363(19.360)	244(13.013)	607(32.373)
M7	Aki Fuji	Color	1893	1231(65.029)	453(23.930)	209(11.041)	662(34.971)
M8	Lele Fuji	Color	1962	1222(62.283)	497(25.331)	243(12.385)	740(37.717)
M9	Aomori-fu No.13	Color	2025	1263(62.370)	363(17.926)	399(19.704)	762(37.630)
M10	2001	Color	1874	1231(65.688)	396(21.131)	247(13.180)	643(34.312)
M11	Aki-fu No.1	Color	1872	1238(66.132)	438(23.397)	196(10.470)	634(33.868)
M12	Aki-fu No.5	Color	1910	1270(66.492)	433(22.670)	207(10.838)	640(33.508)
M13	Morioka-fu No.1	Color	2018	1294(64.123)	363(17.988)	361(17.889)	724(35.877)
M14	Morioka-fu No.2	Color	1950	1289(66.103)	373(19.128)	288(14.769)	661(33.897)
M15	Tensei	Color	1989	1260(63.348)	471(23.680)	258(12.971)	729(36.652)
M16	Qingnonghe No.2	Color	1916	1288(67.223)	387(20.198)	241(12.578)	628(32.777)
M17	Qianxuan No.3	Color	1908	1263(66.195)	380(19.916)	265(13.889)	645(33.805)
M18	Fubrax	Color	1871	1231(65.794)	411(21.967)	229(12.239)	640(34.206)
M19	Nagafu No.12	Color	1939	1287(66.374)	353(18.205)	299(15.420)	652(33.626)
M20	Nagafu No.1	Color	1923	1266(65.835)	341(17.733)	316(16.433)	657(34.165)
M21	Nagafu No.2	Color	1998	1281(64.114)	406(20.320)	311(15.566)	717(35.886)
M22	Nagafu No.4	Color	1928	1272(65.975)	399(20.695)	257(13.330)	656(34.025)
M23	Nagafu No.6	Color	2026	1297(64.018)	372(18.361)	357(17.621)	729(35.982)
M24	Nagafu No.7	Color	1933	1322(68.391)	377(19.503)	234(12.106)	611(31.609)
M25	Nagafu No.36	Color	1904	1201(63.078)	464(24.370)	239(12.553)	703(36.922)
M26	Wengao No.1	Color	1871	1254(67.023)	379(20.257)	238(12.720)	617(32.977)
M27	Wengao-M No.1	Color	1968	1302(66.159)	450(22.866)	216(10.976)	666(33.841)
M28	Wengao No.2	Color	1884	1317(69.904)	332(17.622)	235(12.473)	567(30.096)
M29	Wengao-M No.2	Color	1917	1267(66.093)	358(18.675)	292(15.232)	650(33.907)
M30	Wengao-M No.3	Color	2066	1270(61.471)	438(21.200)	358(17.328)	796(38.529)
M31	Yanfu No.1	Color	1976	1233(62.399)	440(22.267)	303(15.334)	743(37.601)
M32	Yanfu No.2	Color	1940	1249(64.381)	457(23.557)	234(12.062)	691(35.619)
M33	Yanfu No.3	Color	1952	1278(65.471)	449(23.002)	225(11.527)	674(34.529)
M34	Yanfu No.4	Color	1962	1243(63.354)	429(21.865)	290(14.781)	719(36.646)
M35	Yanfu No.5	Color	2014	1300(64.548)	435(21.599)	279(13.853)	714(35.452)
M36	Yanfu No.8	Color	1915	1233(64.386)	445(23.238)	237(12.376)	682(35.614)
M37	Yanfu No.10	Color	1921	1267(65.955)	433(22.540)	221(11.504)	654(34.045)
M38	92-58	Color	2103	1383(65.763)	440(20.922)	280(13.314)	720(34.237)
M39	Meili	Color	1933	1298(67.150)	404(20.900)	231(11.950)	635(32.850)
M40	Meile	Color	2013	1272(63.189)	503(24.988)	238(11.823)	741(36.811)
M41	Tianfu No.1	Color	2004	1310(65.369)	385(19.212)	309(15.419)	694(34.631)
M42	Tianfu No.2	Color	1980	1270(64.141)	462(23.333)	248(12.525)	710(35.859)
M43	Yanchanghong	Color	1947	1259(64.664)	446(22.907)	242(12.429)	688(35.336)
M44	Zhaoyuanstripe red Fuji	Color	2021	1304(64.523)	458(22.662)	259(12.815)	717(35.477)
M45	Zhaoyuanflush red Fuji	Color	2136	1247(58.380)	610(28.558)	279(13.062)	889(41.620)
M46	Shoufu No.1	Color	2031	1281(63.072)	517(25.455)	233(11.472)	750(36.928)
M47	Shoufu No.2	Color	2039	1309(64.198)	518(25.405)	212(10.397)	730(35.802)
M48	Yulindian Fuji	Color	1978	1242(62.791)	493(24.924)	243(12.285)	736(37.209)
M49	Changfujia	Color	2035	1282(62.998)	426(20.934)	327(16.069)	753(37.002)
M50	Zhaofuwang	Color	1885	1290(68.435)	339(17.984)	256(13.581)	595(31.565)
M51	Yishuihong	Color	1893	1160(61.278)	439(23.191)	294(15.531)	733(38.722)

M52	Jihong	Color	1904	1259(66.124)	450(23.634)	195(10.242)	645(33.876)
M53	Yannongzaofu	Early-maturation	1793	1141(63.636)	423(23.592)	229(12.772)	652(36.364)
M54	Zaoshu Fuji	Early-maturation	1884	1116(59.236)	490(26.008)	278(14.756)	768(40.764)
M55	Early Fuji A	Early-maturation	1965	1203(61.221)	471(23.969)	291(14.809)	762(38.779)
M56	Hongjiangjun	Early-maturation	1862	1202(64.554)	411(22.073)	249(13.373)	660(35.446)
M57	Hongwangjiang	Early-maturation	1804	1222(67.738)	301(16.685)	281(15.576)	582(32.262)
M58	Jinfu No.1	Early-maturation	1927	1201(62.325)	491(25.480)	235(12.195)	726(37.675)
M59	Gai Fuji	Early-maturation	1851	1169(63.155)	405(21.880)	277(14.965)	682(36.845)
M60	Yuhuazaofu	Early-maturation	1816	1149(63.271)	391(21.531)	276(15.198)	667(36.729)
M61	Qianxuan No.1	Early-maturation	1847	1220(66.053)	408(22.090)	219(11.857)	627(33.947)
M62	Qianxuan No.2	Early-maturation	1889	1226(64.902)	450(23.822)	213(11.276)	663(35.098)
M63	Changhong	Early-maturation	1923	1231(64.015)	393(20.437)	299(15.549)	692(35.985)
M64	New ryoka	Early-maturation	1884	1219(64.703)	411(21.815)	254(13.482)	665(35.297)
M65	Ryoka	Early-maturation	1862	1165(62.567)	459(24.651)	238(12.782)	697(37.433)
M66	Jinfu No.2	Early-maturation	1855	1213(65.391)	405(21.833)	237(12.776)	642(34.609)
M67	Jinfu No.3	Early-maturation	1892	1217(64.323)	381(20.137)	294(15.539)	675(35.677)
M68	Yishuizhongqiu	Early-maturation	1892	1200(63.425)	448(23.679)	244(12.896)	692(36.575)
M69	Fengfuji No.1	Early-maturation	1888	1160(61.441)	452(23.941)	276(14.619)	728(38.559)
M70	Shoufu No.3	Early-maturation	1928	1232(63.900)	472(24.481)	224(11.618)	696(36.100)
M71	Changyanghong	Early-maturation	2057	1458(70.880)	340(16.529)	259(12.591)	599(29.120)
M72	Yiyuan Nagafu No.2	Early-maturation	1883	1161(61.657)	452(24.004)	270(14.339)	722(38.343)
M73	Hirosaki fuji	Early-maturation	1975	1181(59.797)	565(28.608)	229(11.595)	794(40.203)
M74	Karakida Fuji	Early-maturation	2120	1265(59.670)	497(23.443)	358(16.887)	855(40.330)
M75	Sufuji	Early-maturation	1867	1201(64.328)	381(20.407)	285(15.265)	666(35.672)
M76	Qiufuhong	Spur	2085	1145(54.916)	614(29.448)	326(15.635)	940(45.084)
M77	Yanfu No.6	Spur	1818	1235(67.932)	367(20.187)	216(11.881)	583(32.068)
M78	Yanfu No.7	Spur	1820	1194(65.604)	418(22.967)	208(11.429)	626(34.396)
M79	Huimin spur	Spur	1882	1219(64.772)	440(23.379)	223(11.849)	663(35.228)
M80	Duanzhi Fuji	Spur	1852	1222(65.983)	390(21.058)	240(12.959)	630(34.017)
M81	Miyazaki	Spur	1853	1200(64.760)	399(21.533)	254(13.708)	653(35.240)
M82	Tiaowen Gongqi	Spur	2000	1150(57.500)	583(29.150)	267(13.350)	850(42.500)
M83	Aki-fu No.39	Spur	1934	1182(61.117)	452(23.371)	300(15.512)	752(38.883)
M84	Longfu	Spur	1853	1198(64.652)	399(21.533)	256(13.815)	655(35.348)
M85	Fukushima spur	Spur	1883	1216(64.578)	408(21.668)	259(13.755)	667(35.422)
M86	Aomori spur	Spur	1884	1217(64.597)	461(24.469)	206(10.934)	667(35.403)
M87	Qinfu No.1	Spur	1941	1241(63.936)	445(22.926)	255(13.138)	700(36.064)
M88	Liquan spur	Spur	1840	1177(63.967)	411(22.337)	252(13.696)	663(36.033)
M89	Lingbaoduanfu	Spur	1842	1182(64.169)	440(23.887)	220(11.944)	660(35.831)
M90	Chengji No.1	Spur	1857	1189(64.028)	434(23.371)	234(12.601)	668(35.972)
M91	Yiyuanhong	Spur	1942	1252(64.470)	376(19.361)	314(16.169)	690(35.530)
M92	Shifu spur	Spur	1814	1187(65.436)	413(22.767)	214(11.797)	627(34.564)
**Total**	**Min**		1793	1116(54.916)	301(16.528)	195(10.242)	567(29.120)
	**Max**		2136	1458(70.880)	614(29.448)	483(22.870)	940(45.084)
	**Mean**		1930	1236(64.090)	431(22.311)	263(13.599)	694(35.910)

Nineteen pairs of AFLP primer combinations amplified 1745 total loci in the same test ~~material~~ varieties used for MSAP, among which 1620 were polymorphic loci, accounting for ~~91~~92.837% of the total amplified loci. Different primer combinations produced different amplification results. The total number of amplified loci obtained with a single pair of primers ranged from 36 to 166, the number of polymorphic loci ranged from 28 to 150, and the polymorphic ratio ranged from 57.143% to 100%. On average, the total numbers of loci and polymorphic loci generated by amplification with each pair of primers were 92 and 86, respectively. The four primer combinations (E-AGG+M-CAG, E-AGG+M-CTA, E-ACG+M-CAC, E-ACG+M-CTG) produced 100% polymorphic loci. Primer amplification details are shown in S3 Table.

Analysis of DNA methylation levels and variation patterns at the ~~individual~~varieties level

As shown in Table 1, the global genomic DNA methylation level of the 91

~~genotypes~~varieties~~-~~varieties was 29.120%-45.084%, with an average of 35.~~91~~929%. Among these modifications, the internal cytosine methylation level was 22.311% (16.~~53~~528%-29.~~45~~448%), and the external cytosine methylation level was 13.~~60~~599% (10.242% -22.870%). The former was clearly significantly higher than the latter (*P*<0.01), implying that internal cytosine methylation was the main DMA methylation way in the 'Fuji' line.

According to the cutting profiles of the *Hpa* II and *Msp* I methylation-sensitive endonucleases in the original 'Fuji' variety, the banding patterns could be divided into four types: A, B, C, and D (Table 2). In comparison with the original 'Fuji' ~~variety~~, there were many types of possible locus variation in the mutant varieties, so the variation pattern of methylation ~~sites~~ loci could be subdivided into several subcategories: A1, A2, A3, A4, B1, B2, B3, B4, C1, C2, C3, C4, D1, D2, and D3. As shown in S1 Fig, in DNA methylation variation patterns A and C, ~~sites~~ loci identical to the original methylation pattern presented the highest proportion, indicating that during the occurrence of bud mutations in 'Fuji' line, the majority of ~~sites~~ loci maintained the original methylation pattern, while only a few ~~sites~~ loci exhibited methylation variation (S5 Table). All types of methylation variation patterns (15 subclasses) were detected in the test ~~genotypes~~varieties.

Table 2. Changes in the patterns of cytosine methylation at CCGG sites in the 'Fuji' mutation line.

**Pattern**		**Band type digested by E+H /E+M**		**Pattern**		**Band type digested by E+H /E+M**	
**Type**	**Sub-type**	**Fuji**	**mutation**	**Type**	**Sub-type**	**Fuji**	**Mutation**
A	A1	+/+	+/-	C	C1	-/+	+/-
	A2	+/+	+/+		C2	-/+	+/+
	A3	+/+	-/+		C3	-/+	-/+
	A4	+/+	-/-		C4	-/+	-/-
B	B1	+/-	+/-	D	D1	-/-	+/-
	B2	+/-	+/+		D2	-/-	+/+
	B3	+/-	-/+		D3	-/-	-/+
	B4	+/-	-/-				

Notes: (1) E: *Eco*R Ⅰ enzyme; H: *Hpa* II enzyme; M: *Msp* I enzyme; (2) +: band present; -: band absent; +/+: band present in both E+H and E+M; +/-: band present in E+H but absent in E+M; -/+: band absent in E+H but present in E+M; -/-: band absent in both E+H and E+M.

Fig 1. Correlation analysis of 12 epigenetic parameters. Three asterisks represent a very significant correlation (P < 0. 01). The data distribution is displayed on the diagonal of the matrix; the bivariate scatter with the fitting line is displayed in the lower left; the correlation coefficient and the significance level are displayed in the upper right.

The above 15 subclasses of methylation variation patterns were further categorized into four types: CG hypermethylation (CG-hyper), CHG hypermethylation (CHG-hyper), CG demethylation (CG-hypo) and CHG demethylation (CHG-hypo). As shown in S4 Table, ~~different extents of hypermethylation and demethylation occurred at CCGG sites in 91 samples of 'Fuji' mutants. In general, the frequency of the former~~

Formatted: Font: 12 pt, Italic
Formatted: Font: 12 pt
Formatted: Indent: First line: 1 ch
Formatted: Font: 12 pt, Not Highlight
Formatted: Font: 12 pt
Formatted: Font: 12 pt, Not Highlight
Formatted: Font: 12 pt
Formatted: Font: 12 pt
Formatted: Indent: First line: 1 ch
Formatted: Font: 12 pt, Not Highlight
Formatted: Font: 12 pt

was slightly higher than that of the latter, but the difference was not significant (P=0.656), indicating that hypermethylation and demethylation occurred together during the when mutations arose in ‘Fuji’, but the trend of the genomic impact remained unchanged, which was consistent with the close genealogical relationship between the test genotypesvarieties. Additionally, the mean hypermethylation and demethylation frequencies of CHG were significantly higher than those of CG, indicating that the CHG methylation variation pattern played a major role in methylation pattern variation in the ‘Fuji’ lines.CHG-hypo, CHG-hyper, CG-hyper, and CG-hypo showdisplayed differences among different tested genotypesvarietiesvarieties, but the general trend basically showed the following correlations: CHG-hypo> CHG-hyper > CG-hyper >CG-hypo (S2 Fig). (CHG-hypo+CHG-hyper) was significantly higher than (CG-hypo+CG-hyper) ($P<0.01$). The relative trend between the total demethylation frequency and the hypermethylation frequency in different individualvarietiess also showedexhibited diversity, including the following findings: (1) the demethylation frequency was higher than the frequency of methylation, (2) the hypermethylation frequency was higher than that of demethylation, and (3) hypermethylation and demethylation frequencies were approximately the same;. tThese results indicated that methylation pattern variations were not fixed during the occurrence of the ‘Fuji’ mutation.

CHG-hypo, CHG-hyper, CG-hyper, and CG-hypo showed differences among different tested genotypesvarieties, but the general trend basically showed the following correlations: CHG-hypo> CHG-hyper > CG-hyper >CG-hypo (S2 Fig). The relative trend between the total demethylation frequency and the hypermethylation frequency in different individualvarietiess also showed diversity, including the following findings: (1) the demethylation frequency was higher than the frequency of methylation, (2) the hypermethylation frequency was higher than that of demethylation, and (3) hypermethylation and demethylation frequencies were approximately the same; these results indicated that methylation pattern variations were not fixed during the occurrence of the ‘Fuji’ mutation. This phenomenon might be associated with the factors that lead to mutations occurring in real environmental conditions.

Analysis of the DNA methylation level and variation pattern at the mutant group level

The epigenetic relationships of the genomes of different mutant groups in the ‘Fuji’ line were analyzed using 12 parameters related to DNA methylation levels and patterns (V5-V16). Table 3 shows showed that the variation coefficients (CVs) of V5, V6, V7, V8, V9, and V10 were generally greater than those of V11, V12, V13, V14, V15, and V16. Among these 12 parameters, V5-V10 and V11-V16 reflected variations in the DNA methylation pattern and DNA methylation level, respectively. Therefore, it could be deduced from the CVs between these two groups of parameters that the variation of methylation patterns among individualvarieties was greater than that of methylation levels. V11-V16, V11, V12, V15, and V16 exhibited the same degree of variation among groups, while V13 and V14 showed relatively higher CV than these groups,

Formatted: Font: 12 pt, Italic

Formatted: Font: 12 pt

Formatted: Font: 12 pt, Font color: Red

Formatted: Font: 12 pt

Formatted: Font: 12 pt, Font color: Red

Formatted: Font: 12 pt

Formatted: Font: 12 pt

indicating that variation in CHG and CG methylation levels is abundant among individualvarieties of different mutant groups in the 'Fuji' line.

Table 3. Variations in the levels of 12 DNA methylation parameters and pattern-related parameters in three 'Fuji' mutant groups.

Parameter	Std.Deviation			Mean			CV (%)		
	Spur	Early-maturation	Color	Spur	Early-maturation	Color	Spur	Early-maturation	Color
V5	2.033	2.776	1.802	12.722	13.571	14.608	15.977	20.454	12.338
V6	1.412	2.436	1.896	9.833	10.796	13.463	14.363	22.563	14.081
V7	1.965	2.558	2.732	6.682	7.346	10.223	29.401	34.817	26.726
V8	3.239	2.466	2.846	14.265	14.977	18.743	22.703	16.464	15.182
V9	2.848	4.946	3.342	22.555	24.367	28.072	12.626	20.299	11.907
V10	3.691	4.267	3.462	20.947	22.324	28.966	17.622	19.115	11.953
V11	3.141	2.616	2.605	36.328	36.427	35.572	8.645	7.181	7.324
V12	3.141	2.616	2.605	63.672	63.573	64.428	4.932	4.115	4.044
V13	1.523	1.583	2.464	13.187	13.77	13.679	11.548	11.497	18.016
V14	2.668	2.734	2.675	23.142	22.656	21.894	11.531	12.068	12.218
V15	2.444	2.552	2.260	35.334	34.989	32.948	6.917	7.293	6.854
V16	72.196	75.503	66.710	1889	1898	1959	3.823	3.978	3.406

Notes: V5, CHG hyper-methylation frequency; V6, CG hyper-methylation frequency; V7, CG hypo-methylation frequency; V8, CHG hyper-methylation frequency; V9, the total hyper-methylation frequency; V10, the total hypo-methylation frequency; V11, total genome methylation frequency; V12, the frequency of condition I (Non-methylation frequency); V13, the frequency of condition II methylation (CHG methylation frequency); V14, the frequency of condition III methylation (CG methylation ratio); V15, the frequency of condition IV methylation; V16, the total amplified loci of varieties.

Parameter	Std.Deviation			Mean			CV (%)		
	Spur	Early-maturation	Color	Spur	Early-maturation	Color	Spur	Early-maturation	Color
V5	2.03	2.78	1.80	12.72	13.57	14.61	15.98	20.45	12.34
V6	1.41	2.44	1.90	9.83	10.80	13.46	14.36	22.56	14.08
V7	1.96	2.56	2.73	6.68	7.35	10.22	29.40	34.82	26.73
V8	3.24	2.47	2.85	14.27	14.98	18.74	22.70	16.46	15.18
V9	2.85	4.95	3.34	22.56	24.37	28.07	12.63	20.30	11.91
V10	3.69	4.27	3.46	20.95	22.32	28.97	17.62	19.12	11.95
V11	3.14	2.62	2.61	36.33	36.43	35.57	8.64	7.18	7.32
V12	3.14	2.62	2.61	63.67	63.57	64.43	4.93	4.11	4.04
V13	1.52	1.58	2.46	13.19	13.77	13.68	11.55	11.50	18.02
V14	2.67	2.73	2.67	23.14	22.66	21.89	11.53	12.07	12.22
V15	2.44	2.55	2.26	35.33	34.99	32.95	6.92	7.29	6.85
V16	72.20	75.50	66.71	1888	1898	1959	3.82	3.98	3.41

Notes: V5, CHG hyper-methylation frequency; V6, CG hyper-methylation frequency; V7, CG hypo-methylation frequency; V8, CHG hyper-methylation frequency; V9, the total hyper-methylation frequency; V10, the total hypo-methylation frequency; V11, total genome methylation frequency; V12, the frequency of condition I (Non-methylation frequency); V13, the frequency of condition II methylation (CHG methylation frequency); V14, the frequency of condition III methylation (CG methylation ratio); V15, the frequency of condition IV methylation; V16, the total amplified loci of genotypesvarieties.

The CVs of each parameter were compared between the three mutant groups and analyzed (Table 3). It was shown that the emphases of epigenetic variation were

Formatted Table

Formatted: Font: 12 pt

different in different groups; for example, ~~for~~ in the~~-~~ ~~early~~Early-maturation group, several parameters, including V5, V6, V7, V9, and V10, presented higher values than in the other groups; in the ~~spur~~ Spur group, V8 was much more prominent; in the ~~color~~ Color group, V13 showed a remarkably high value. Based on the above results, it seemed that the variation in the methylation level was more important in the ~~color~~ Color group. The variation in the methylation pattern was obvious in the~~-~~ ~~early~~Early-maturation and ~~spur~~ Spur groups; moreover, the extent of the variation in the~~-e~~ Early-maturation group was much wider.

The correlations of 12 DNA methylation parameters were calculated and plotted using the R packages *psych* and *corrplot*. As presented in Fig 1, a total of 43 pairs (*P*<0.01) were significantly correlated with each other, among which V6 and V9 exhibited the highest significant positive correlation (0.93), followed by V5 and V9 (0.91). The lowest correlation was found for V10 and V13 (0.25), followed by V9 and V12 (0.31). The significant negative correlation was highest between V5 and V11 (-0.24), followed by V10 and V14 (-0.27). The lowest negative correlation was found for V15 and V16 (-1.00), followed by V10 and V15 (-0.74). The results showed that there was a direct positive correlation between the total hypermethylation frequency and the frequency of CG hypermethylation (that is, the higher the frequency of CG hypermethylation, the higher the frequency of total hypermethylation). The total demethylation frequency presented a small correlation with the proportion of CHG methylation but a significant negative correlation with the proportion of CG methylation (that is, the higher the proportion of CG methylation, the lower the total demethylation frequency).

Furthermore, the above 12 DNA methylation parameters of the three mutant groups were compared and analyzed. The data distribution of all 12 variables conformed to a normal distribution and the assumptions of the statistical analysis. The variance homogeneity test showed that, with the exception of V9 (the total hypermethylation frequency), the other 11 parameters all met the requirements of homogeneity of variance. Therefore, the Tamhane's T2 and Games-Howell tests were chosen for V9, whereas the Duncan and LSD tests were chosen for the other parameters for multiple comparison analysis with SPSS software. Multiple comparative analysis among groups~~AMOVA~~ showed that among the three mutant groups, V5, V6, V7, V8, V9, V10, V15, and V16 presented significant differences (*P*<0.01), but no significant difference was found between V11, V12, V13, and V14. The average multiple comparison results revealed that the differences between the ~~spur~~ Spur and~~-e~~ Early-maturation groups were not significant, showing similar epigenetic characteristics, but different degrees of significant differences existed between the ~~spur~~ Spur and ~~color~~ Color mutant groups as well as between the~~-e~~ Early-maturation and ~~color~~ Color groups. For example, V5, V6, V7, V8, V9, V10, V15, and V16 were significantly different between the ~~spur~~ Spur and ~~color~~ Color mutant groups; the difference in V6, V7, V8, V9, V10, V15, and V16 between the ~~early~~Early-maturation and ~~color~~ Color mutant groups was very significant (Fig 2). Taken together, the above results showed that among the three 'Fuji' mutant groups, the ~~spur~~ Spur and~~-e~~ Early-maturation groups showed similar epigenetic patterns; however, between the ~~color~~ Color groups and either the ~~spur~~ Spur group or

Formatted: Font: 12 pt, Italic

Formatted: Font: 12 pt

the~~ e~~ Early-maturation group, epigenetic differences were apparent.

Fig 2. AMOVA of 12 epigenetic parameters between different mutant groups in the 'Fuji' mutation line. Blue, green, and red indicate the materials from the ~~spur~~ Spur group, the ~~early~~Early-maturation group, and the ~~color~~ Color mutant group, respectively.

Epigenetic similarity calculation, clustering and principal component analysis

The results of the genetic similarity analysis based on the ~~methylation-sensitive polymorphic matrix (MSL)~~MSAP-MSL data sets (S6 Table) showed that the epigenetic similarity coefficient between ~~individual~~varietiess~~ ~~varieties ranged from 0.~~52~~ 515 to 0.~~81~~807; the lowest similarity was found between 'Yishuizhongqiu' and 'Wengao No.3', and the highest similarity existed between 'Longfu' and 'Liquan spur'. The average epigenetic similarity between ~~individual~~varietiess in the 'Fuji' mutation line was 0.~~64~~639, ranging from 0.~~58~~577~~~-~~0.678. The mean similarity coefficient between the standard 'Fuji' variety and its descendant varieties was 0.648, ranging from 0.561 (between 'Fuji' and 'Zhaoyuanflush red Fuji') to 0.~~76~~ 758 (between 'Fuji' and 'Yanfu No.7'). The results showed that there was ~~significant~~ apparently epigenetic variation between mutant ~~individual~~varieties and their 'Fuji' mother, and different degrees of epigenetic differentiation also occurred between different mutant ~~individual~~varieties.

The UPGMA results showed (Fig 3(A)) that nearly 84% (43/51) of the ~~genotypes~~varieties~~-~~varieties in the ~~color~~ Color mutant group clustered closely together to form an independent cluster (Cluster 1). The~~ e~~ Early-maturation mutant group, the ~~spur~~Spur-maturation mutant group, 'Fuji', and the remaining eight ~~genotypes~~varieties of the ~~color~~ Color mutant group formed another group, designated Cluster 2. In comparison with the position of 'Fuji', other ~~genotypes~~varieties showed obvious epigenetic variation, and the epigenetic variation of the ~~color~~ Color mutant group was most prominent. We also found that within Cluster 2, the ~~early~~Early-maturation group and ~~spur~~ Spur mutant group were mixed and distributed without distinct grouping, reflecting the close epigenetic relationship between them. In addition, although 'Chang ~~'~~Fujia', 'Zhaofuwang', 'Yishuihong' and 'Jihong' in the ~~color~~ Color mutant group were concentrated in Cluster 2, they were relatively closer to Cluster 1, but 'Aki-fu No.5', 'Line Ⅰ ~~'~~Fuji'~~'~~, 'Iwate line Ⅰ', and 'Iwafu No.10' were located far from Cluster 1. These four ~~genotypes~~varieties were all selected in Japan and belonged to the ~~color~~ Color mutant group. Moreover, they were the earliest mutant cultivars obtained from 'Fuji', and few clonal descendants are selected from these cultivars at present. Therefore, their clustering results might be related to their original region of selection or breeding generation. In conclusion, the UPGMA results implied that the greatest epigenetic variation existed in the ~~color~~ Color mutant group, which might present unique epigenetic and evolutionary mechanisms compared with the other two variant groups.

Formatted: Font: 12 pt

Formatted: Font: 12 pt

Fig 3. Dendrogram of 92 ~~genotypes~~varieties obtained from UPGMA cluster analysis based on MSAP-MSL (A) and AFLP (B) data and the distribution of 16 methylation parameters of V5-V15 in the heat map between samples. Blue, green, and red in the circle of interest indicate the materials from the ~~spur~~ Spur group, the ~~early~~Early-maturation group, and the ~~color~~ Color group, respectively.

The results of principal component analysis further supported the UPGMA clustering results. Principal components PC1 and PC2 accounted for 13.6% and 4.5% of the total variation, respectively. As shown in Fig 4 (A), the ~~spur~~ Spur mutant and~~ e~~ Early-maturation groups were mixed~~ together~~, showing similar epigenetic consistency. ‘Fuji’ was included in this group, displaying a close epigenetic relationship with its members. The ~~color~~ Color mutant group was basically independent and was located far from ‘Fuji’ and the other two mutant groups, indicating its unique epigenetic variation.

Formatted: Font: 12 pt

Fig 4. Principal coordinate analysis (PCA) of 92 ~~genotypes~~varieties based on MSAP-MSL (A) and AFLP (B). Blue, green, and red indicate the materials from the ~~spur~~ Spur group, the ~~early~~Early-maturation group, and the ~~color~~ Color group, respectively.

Genetic similarity calculation, clustering and principal component analysis

~~GUsing NTSYS, g~~genetic similarities of 0.653 (between ‘Miyazaki’ and ‘Su Fuji’) to 0.~~84~~ 899 (between ‘Nagafu No.12’ and ‘Nagafu No.2’) were detected by NTSYS in the ‘Fuji’ mutation line, with an average similarity of 0.~~81~~805 (S7 Table). The mean similarity coefficient between the standard ‘Fuji’ variety and its mutants was 0.80, ranging from 0.684 (between ‘Fuji’ and ‘Su Fuji’) to 0.~~88~~ 877 (between ‘Fuji’ and ‘Yanfu No.7’), indicating that the genetic variation of the mutants differed from that of ‘Fuji’’.

Formatted: Font: 12 pt

The UPGMA results showed (Fig 3(B)) that 92 samples could be clustered into four groups (Clusters 1-4), and ‘Aki-fu No.5’ was clustered in the outermost area and separated into different groups, indicating distant genetic similarity. In addition, ‘Hongwangjiang’, ‘Gai Fuji’, and ‘Su Fuji’ were grouped together to form Cluster 3. Cluster 1 and Cluster 2 were closely related to each other. ‘Fuji’ was clustered in Cluster 1, which was closely related to ‘Shanfu No.6’, ‘Qiufuhong’, ‘Lele Fuji’, and ‘Qingnonghe No.1’. In Cluster 1 and Cluster 2, local clustering and accumulation phenomena of the same ‘Fuji’ mutation type were found, as observed in the regions between ‘Yanfu No.7’ and ‘Duanzhi Fuji’ (~~spur~~ Spur mutant group), ‘Aki Fuji’ and ‘Nagafu No.36’ (~~color~~ Color mutant group), and ‘Miyazaki’ and ‘Chengji No.1’ (~~spur~~ Spur mutant group) in the former group and the region between ‘Qianxuan No.1’ and ‘Shoufu No.3’ and that between ‘Yanfu No.3’ and ‘Nagafu No.4’ in the latter group. However, from the overall perspective of the cluster diagram, the three major ‘Fuji’ mutant groups were mixed and arranged, and there was no obvious boundary between different groups. Fig 4 (B) showed the PCA results of the 92 test ~~genotypes~~varieties. The three dimensions of PC1 and PC2, PC1 and PC3, and PC2 and PC3 all showed

results that were consistent with UPGMA clustering; that is, the genetic similarity among the three major mutation types of 'Fuji' was very high, without obvious boundaries.

Epigenetic and genetic diversity and molecular variation analysis

As shown in Table 4, the mean Shannon index, the number of effective alleles and the mean expected heterozygosity based on MSL data among the three mutant groups were 0.415, 1.424, and 0.266, respectively. In general, the three genetic diversity indexes showed a consistent variation trend. For mutants with a high Shannon index (I), the effective allelic variance (Ne) and mean expected heterozygosity (He) were also higher. ~~The~~The I and He indices presented significant differences among the three mutant groups (*P*<0.01). The analysis of molecular variance (Table 5) showed that most of the variation occurred within the mutant groups (85%), and only a very small proportion (15%) occurred among the mutant groups (*P*< 0.01). ~~epigenetic diversitHe and I forthe color mutant group was significantly higher than those of both the spur and early-maturation mutant groups. The early-maturation and spur groups presented similar epigenetic diversity values, although that of the latter was higher than that of the former.~~

Table 4. Epigenetic and genetic similarity and diversity in three different mutant groups of the 'Fuji' line.

Molecular marker type	Group	Percentage of Polymorphic Loci (%)	Epigenetic/Genetic diversity parameter		
			I	Ne	He
MSAP	Spur	82.895	0.388A	1.404A	0.250A
	Early-maturation	92.677	0.413B	1.428AB	0.266B
	Color	99.542	0.443C	1.440B	0.282C
	Mean	91.705	0.415	1.424	0.266
AFLP	Spur	60.516	0.284a	1.300a	0.183a
	Early-maturation	70.029	0.289a	1.307a	0.186a
	Color	80.573	0.296a	1.303a	0.188a
	Mean	70.372	0.290	1.303	0.186

Abbreviations: Ne, No. of effective alleles; I, Shannon index; He, Expected heterozygosity. Lower case letters indicate a significant difference at the 0.05 level. Lowercase letters indicate a significant difference at the 0.01 level.

~~Molecular marker type~~	~~Group~~	~~Percentage of Polymorphic Loci (%)~~	~~Genetic diversity parameter~~		
			~~I (SD)~~	~~Ne (SD)~~	~~He (SD)~~
~~MSAP~~	~~Spur~~	~~87.36~~	~~0.39(0.005)~~	~~1.40(0.008)~~	~~0.25(0.004)~~
	~~Early-maturation~~	~~90.10~~	~~0.40(0.005)~~	~~1.42(0.008)~~	~~0.27(0.004)~~
	~~Color~~	~~99.66~~	~~0.45(0.004)~~	~~1.44(0.006)~~	~~0.28(0.003)~~
	~~Mean~~	~~92.37~~	~~0.41(0.005)~~	~~1.42(0.007)~~	~~0.26(0.004)~~
~~AFLP~~	~~Spur~~	~~61.83~~	~~0.27(0.006)~~	~~1.28(0.008)~~	~~0.17(0.004)~~
	~~Early-maturation~~	~~64.81~~	~~0.267(0.006)~~	~~1.27(0.008)~~	~~0.17(0.004)~~
	~~Color~~	~~83.50~~	~~0.31(0.006)~~	~~1.32(0.008)~~	~~0.20(0.004)~~
	~~Mean~~	~~52.54~~	~~0.28(0.006)~~	~~1.29(0.008)~~	~~0.18(0.004)~~

~~Abbreviations: Ne, No. of effective alleles; I, Shannon index; He, Expected heterozygosity; SD, standard deviation.~~

Formatted: Font: 12 pt

Formatted: Font: (Default) Times New Roman, 12 pt

Formatted: Font: 12 pt

Formatted: Font: (Default) Times New Roman, 12 pt

Formatted: Font: 12 pt

The analysis of molecular variance (Table 5) showed that most of the variation occurred within the mutant groups (88%), and only a very small portion (12%) occurred among the mutant groups. The input binary distance matrix was used to calculate the value of PhiPT, and locus-by-locus AMOVA showed (Table 6) that the number of loci in the interval of PhiPT=0-0.05 was the greatest (467), accounting for 26.72% of the total (1748), but there were no loci showing significant differentiation. There were slightly fewer loci in the interval of PhiPT=0.05-0.1 (460) than in PhiPT=0-0.05 interval. In this section, 120 significantly differentiated loci were detected (P<0.01), and 368 differentiated loci were detected (P<0.05), accounting for 21.05% and 6.87% of the total loci, respectively. The number of loci in PhiPT=0.15-0.25 and PhiPT>2.5 was significantly lower than that in the other two intervals, but these loci were highly differentiated, accounting for 10.53% and 8.92% of the total number of loci, respectively.

Table 5. Comparisons of the results of analysis of molecular variance (AMOVA), pairwise AMOVA (PhiPT), and the mean similarity between different mutant groups of the 'Fuji' line based on MSAP-MSL and AFLP assays.

Molecular marker type	Compared mutation groups	AMOVA	Pariwise AMOVA (PhiPT)	Mean of Nei's unbiased similarity
AFLP	Among Groups	5%	—	—
—	Within Groups	95%	—	—
MSAP	Among Groups	12%	—	—
—	Within Groups	88%	—	—
AFLP	Spur & Early-maturation	—	0.053	0.98
—	Spur & Color	—	0.055	0.98
—	Early-maturation & Color	—	0.052	0.99
—	Mean	—	0.053	0.98
MSAP	Spur & Early-maturation	—	0.11	0.98
—	Spur & Color	—	0.13	0.95
—	Early-maturation & Color	—	0.13	0.95
—	Mean	—	0.12	0.96

Furthermore, pairwise AMOVA of the genetic differentiation coefficients between different 'Fuji' mutant groups was conducted. Nei's unbiased genetic similarity index was calculated on the basis of comparisons between the groups. The average epigenetic differentiation coefficient was 0.12. The epigenetic differentiation coefficients between the color mutant and the spur mutant groups, between the color mutant and the early-maturation mutant groups, and between the spur mutant and the early-maturation mutant groups were 0.13, 0.13, and 0.03, respectively, suggesting that extremely weak epigenetic differentiation existed between the spur mutant and the early-maturation mutant groups; however, compared to the other two groups, the color mutant group exhibited obvious differentiation.

Nei's unbiased genetic similarity index was calculated on the basis of comparisons between the groups. The results showed that the epigenetic similarity between the color mutant and the spur mutant groups, between the color mutant and the early-maturation mutant groups, and between the spur mutant and the early-maturation mutant groups

Formatted: Font: 12 pt

~~was 0.95, 0.95, and 0.98, respectively, with an average of 0.97, reflecting the extremely high epigenetic similarity between groups. The color mutant groups showed relatively low epigenetic similarity with the other two groups, while the similarity between the spur and the early-maturation mutant groups was relatively high, which was consistent with the above epigenetic differentiation results.~~

T~~able 4 showed that t~~he average Shannon index, number of effective alleles and average expected degree of heterozygosity in the three 'Fuji' mutant groups reflected by AFLP analysis were 0.~~28~~290, 1.~~29~~ 303 and 0.186, respectively. In general, there was no significant difference in genetic diversity indexes between the three groups based on AFLP data. AMOVA showed that most of the genetic variation existed within the mutant groups (95%); a very small portion (5%) existed between the populations (*P*< 0.01). ~~the three genetic diversity indexes showed a consistent variation trend. For the mutants with a high Shannon index, the effective allelic variance (Ne) and mean expected heterozygosity (He) were also higher. The genetic diversity of the color mutant group was significantly higher than that of the spur and early-maturation mutant groups. The genetic diversity of the spur and early-maturation mutant groups was similar, but that of the former was slightly higher than that of the latter.~~

~~AMOVA (Table 5) showed that most of the genetic variation existed within the mutant groups (95%), and a very small portion (5%) existed between the populations; (significance test, P< 0.01). According to the locus-by-locus AMOVA (Table 6), the number of loci located in the interval of PhiPT=0.05-0.15 was the greatest (786), accounting for 45.043% of the total loci (1745). Among these loci, 50 showed significant differentiation between groups, corresponding to 2.87% of the total loci. The number of loci in the PhiPT=0-0.05 interval was similar to that in PhiPT=0.15-0.25, but no loci showing significant differentiation were detected in the former, while the loci in the latter were all significantly differentiated. The number of loci in PhiPT > 2.5 was the lowest, but all of these loci exhibited significant genetic differentiation, corresponding to 1.43% of the total loci. The results showed that there was little genetic differentiation between different 'Fuji' mutant groups.~~

~~Further pairwise AMOVA (Table 5) between different groups of 'Fuji' mutants showed that the genetic differentiation indexes between the color mutant group and spur mutant group, the color mutant group and the early-maturation mutant group, and the spur mutant group and the early mutant group were 0.06, 0.05, and 0.05, respectively (average 0.05), which confirmed the extremely weak genetic differentiation between different groups of 'Fuji' mutants. Nei's unbiased genetic similarity coefficient was calculated on the basis of pairwise comparisons between mutant groups (Table 4). The results showed that the genetic similarities between the color mutant group and the spur mutant group, the color mutant group and the early-maturation mutant group, and the spur mutant group and early-maturation mutant group were 0.98, 0.99, and 0.98, with an average of 0.98, which reflected the extremely high genetic similarity between the groups.~~

Epigenetic and genetic structure analysis

Structure analysis was carried out based on MSL epigenetic data and AFLP-based

Formatted: Font: (Default) Times New Roman, 12 pt

Formatted: Font: 12 pt

Formatted: Font: (Default) Times New Roman, 12 pt

Formatted: Font: 12 pt

Formatted: Font: (Default) Times New Roman, 12 pt

Formatted: Font: 12 pt

Formatted: Indent: First line: 1.5 ch

Formatted: Font: 12 pt

genetic data, and 10,000 simulations were run with population numbers K=2 to 4. The results showed that LnP (D) values were generated for K=3 for both types of data, and no values were generated for K= 2 or K= 4. Therefore, K=3 was selected as the optimal number of clusters. Fig 5(A) ~~shows~~ showed that ~~the lineages of~~in the ‘Fuji’ mutation line, the ~~spur~~ Spur ~~varieties~~ mutant group and the ~~early~~Early-maturation ~~varieties~~ mutant group were highly consistent, while the ~~color~~ Color mutant group showed a large proportion of heteromorphic lineages, indicating the unique epigenetic structure of the ~~color~~ Color mutant group. As shown in Fig 5(B), there was no clear division between any of the mutant groups, which reflected the similarity of their genetic compositions.

Fig 5. Population structure based on MSAP-MSL (A) and AFLP (B) analysis for ‘Fuji’ and its mutants.

Outlier analysis

Bayesian population genomic analysis showed that out of 1748 methylation-sensitive MSAP ~~sites~~loci, 601 exhibited positive alpha values, and 1147 exhibited negative alpha values. A positive alpha value indicates positive or diversified selection, while negative values indicate the negative or purifying selection, also known as neutral selection. Thus, purifying selection seemed to play a major role in the differentiation of the different groups of ‘Fuji’ mutants (65.~~62~~618%). However, epigenetic diversity selection still occurred at nearly 35% of loci. Sixteen epigenetic outlier loci were detected, accounting for 0.~~92~~915% of the total DNA methylation-sensitive loci (Fig 6(A)). The epigenetic genetic frequencies of each locus in each mutant group were calculated ~~individual~~varietyly. If the frequency of a locus in a group was greater than 50%, it was set as a specific outlier locus of that population. Therefore, Fig 7 showed that there were 13~~, ,~~ 13 and 3 methylated outlier loci in the ~~spur~~Spur, ~~early~~Early-maturation and ~~color~~ Color mutant groups, respectively. According to the profile of the frequency of loci, the ~~spur~~ Spur and~~ e~~ Early-maturation mutant groups presented the same pattern, which basically maintained the state of the existence of the majority of loci in ‘Fuji’. However, the pattern of the ~~color~~ Color mutant group was completely different from that of the other two mutant groups as well as ‘Fuji’. In the ~~color~~ Color mutant group, 13 locus deletions and 3 new locus mutations occurred, indicating different epigenetic patterns of the outlier loci among the different mutant groups and suggesting unique epigenetic characteristics of the ~~color~~ Color mutant groups.

Fig 6. Bayesian genomic scans of the three mutant groups in the ‘Fuji’ line to identify outlier loci based on MSAP-MSL (A) and AFLP data (B).

Fig 7. The frequency of 16 outlier loci in 3 different groups of the ‘Fuji’ mutation line based on the MSAP-MSL assay. Blue, green, and red indicate the materials from the ~~spur~~ Spur group, the ~~early~~Early-maturation group, and the ~~color~~ Color group, respectively.

According to AFLP analysis, among the 1745 amplified loci, Bayesian population

Formatted: Font: 12 pt

Formatted: Font: 12 pt

genomic analysis generated 568 ~~sites~~ loci with positive alpha values and 1177 ~~sites~~ loci with negative values. Purifying selection played a major role (67.450%) in the differentiation of the different ‘Fuji’ mutant groups. Genetic diversity selection occurred at only a few loci (32.550%). At the level of FDR=0.05, the number of outlier loci detected was 0, indicating that there was no significant difference in genetic differentiation between the three ‘Fuji’ mutant groups (Fig 6(B)).

Mantel correlation test of epigenetic and genetic similarity between different mutant groups

The Mantel test was conducted for the genetic and epigenetic similarity coefficient matrix obtained based on MSAP and AFLP analysis. The results showed that the r values of the genetic and epigenetic correlation coefficients of the ~~spur~~ Spur mutant group, ~~early~~Early-maturation mutant group and ~~color~~ Color mutant groups were -0.044 (P=0.368), 0.~~33~~327 (P=0.978), and 0.~~11~~107 (P=0.924), respectively. The overall correlation coefficient was 0.~~11~~108 (P=0.987). The above results showed that there was a slight correlation between genetic and epigenetic variation in the ‘Fuji’ mutation lines, suggesting that the epigenetic variation was ~~partially dependent on genetic variation but was also~~ independent of genetic variation.

Formatted: Font: 12 pt

Comparison of epigenetic and genetic diversity between different mutant groups

As shown in Table 4, the average polymorphic loci ratio~~ and~~ , the polymorphic loci ratio, as well as epigenetic/genetic diversity parameters of each ‘Fuji’ mutant group based on MSAP were significantly higher than those obtained based on AFLP analysis ($P<0.01$)~~, and the epigenetic similarity based on the former was significantly lower than that based on the latter. Regarding the pairwise similarity between the three mutant groups, the difference between the results based on MSAP and AFLP was not as large as the differences for the proportion of polymorphic loci and the genetic diversity parameters, but the average similarity coefficient between groups was significantly different (p <0.05). The above results~~ ~~indicated~~ indicating that the epigenetic diversity of each mutant group in the ‘Fuji’ mutation line was significantly higher than the genetic diversity. For AMOVA between as well as within mutant group, a certain degree of differentiation under both markers was revealed, and among groups, the epigenetic differentiation ratio (15%) was greater than genetic differentiation ratio (5%)~~, the existence of more epigenetic variation than genetic variation. The analysis results for the three genetic diversity parameters were completely consistent with the results of the polymorphic proportion analysis, which further indicated that the epigenetic diversity of each mutant group in the ‘Fuji’ mutation line was significantly higher than the genetic diversity~~.

Formatted: Font: 12 pt

Formatted: Font: 12 pt

~~Comparison of epigenetic and genetic differentiation between different mutant groups~~

By comparing the results of AMOVA with the AFLP and MSAP results (Table 5), it could be seen that the mutant groups showed a certain degree of differentiation according to both markers, and the epigenetic differentiation ratios within and between the groups were greater than the genetic differentiation ratios. The group mean PhiPT results (genetic: 0.05; epigenetic: 0.12) showed that the degree of epigenetic differentiation was also higher than that of genetic differentiation.

The results of pairwise PhiPT (Table 5) between groups showed that the degree of differentiation between the three mutant groups was similar, but the epigenetic variation was significant. The epigenetic differentiation value (0.03) between the spur and early-maturation groups was smaller than the genetic differentiation value (0.05), indicating that there was slight epigenetic differentiation between the two groups. The epigenetic differentiation values between the color mutant group and the spur mutant group and between the color mutant group and the early maturation mutant group were higher than the genetic differentiation values, indicating that significant epigenetic differentiation existed.

The results of locus-by-locus AMOVA of the MSAP and AFLP results (Table 6) showed that, except for PhiPT=0.05-0.15, the total number of sites in the other intervals of the genetic differentiation indexes was greater for the former than for the latter. It is generally believed that a PhiPT differentiation index between 0 and 0.05 indicates weak intergroup differentiation; a PhiPT differentiation index between 0.05 and 0.15 indicates moderate intergroup differentiation; a PhiPT differentiation index between 0.15-0.25 indicates great intergroup differentiation; and a PhiPT differentiation index greater than 0.25 indicates great intergroup differentiation [46]. Our results showed that in the PhiPT=0-0.05 interval, the total number of MSAP loci was significantly higher than the total number of AFLP loci, but the PhiPT results for these loci showed no significant difference, indicating that there was no genetic or epigenetic difference between the weakly differentiated loci of the three 'Fuji' mutant groups. In the interval of PhiPT=0.05-0.15, although the total number of MSAP loci was much lower than that obtained from AFLP analysis, the proportions of loci exhibiting significant differences and different PhiPT values were both greater for the former than for the latter. Therefore, it was inferred epigenetic factors were associated with more loci with a moderate degree of differentiation than genetic factors in general. In the two intervals of Phi PT=0.15-0.25 and Phi PT> 0.25, all sites produced by both MSAP and AFLP in these regions exhibited significant differences, and the proportion in the former was greater than that in the latter, suggesting that epigenetic diversity also resulted in more sites with significant and very significant differentiation and that epigenetic differentiation was significantly higher than genetic differentiation. In conclusion, the results of the comparison between epigenetic and genetic differentiation showed that during the formation of epigenetic modifications, most of the sites in the 'Fuji' mutant groups were at least moderately differentiated.

Table 6. Locus-by-locus AMOVA for three different mutant groups of the 'Fuji' line based on MSAP-MSL and AFLP assays.

Interval of PhiPT	Total	P<=0.05	P<=0.01	P>0.05

Formatted: Font: 12 pt

Formatted: Indent: First line: 2 ch

Formatted: Centered

Formatted: Font: 9 pt, Not Bold

	~~AFLP~~	~~MSAP~~	~~AFLP~~	~~MSAP~~	~~AFLP~~	~~MSAP~~	~~AFLP~~	~~MSAP~~
~~0-0.05~~	~~50~~	~~467~~	~~0~~	~~0~~	~~0~~	~~0~~	~~50~~	~~50~~
~~0.05-0.15~~	~~786~~	~~460~~	~~194~~	~~368~~	~~46~~	~~120~~	~~592~~	~~92~~
~~0.15-0.25~~	~~48~~	~~184~~	~~48~~	~~184~~	~~48~~	~~184~~	~~0~~	~~0~~
~~>0.25~~	~~25~~	~~156~~	~~25~~	~~156~~	~~25~~	~~156~~	~~0~~	~~0~~

Comparison of epigenetic and genetic structure between different mutant groups

The results of the UPGMA clustering analysis, PCA, and structure analysis based on AFLP and MSAP data showed that the three ‘Fuji’ mutant groups were genetically similar and that, regardless of what algorithm the analysis was based on, they could not be clearly separated. In terms of epigenetics, there was a close relationship between the ~~spur~~ Spur and the ~~e~~ Early-maturation mutant groups, which were always mixed ~~together~~, similar to their genetic relationship. However, for the ~~individual~~varieties of the ~~color~~ Color mutant group tended to cluster together, and this group was independent of the other two mutant groups, showing its unique epigenetic structure.

Formatted: Font: 12 pt

Comparison of outlier loci between different mutant groups

The numbers of loci with positive and negative alpha values were approximately the same when generated on the basis of AFLP and MSAP. For each marker type, the proportion of loci with a negative alpha value was greater than that with a positive alpha value. Regarding outlier loci, MSAP analysis detected 16 loci, whereas AFLP analysis detected 0, indicating that epigenetics played a large role in the differentiation of the ‘Fuji’ mutant groups~~,~~ ; and from the viewpoint of the frequency of outlier loci between different groups, epigenetics in the ~~color~~ Color mutant group contributed greatly to the differentiation of the ‘Fuji’ mutant groups.

Formatted: Font: 12 pt

Discussion

~~In recent years, extensive~~ Extensive epigenetic research ~~studies have~~ has been carried out in many ~~cultivated crops and model~~ plant species, whereas ~~but~~ study on the fruit crops is still in progress. So far, the available reports mainly consist of several popular fruit crops such as apple [39-48], orange [49-50], banana [51], pear [52-53], strawberry [54], and grape [55-56]. ~~there is still a lack of studies on fruit crops. In particular,~~ ~~e~~Epigenetic studies ~~of~~ in bud mutations are just in the initial stages since limited literature is available [40-41, 45-47, 52-53]. In ‘Fuji’ apple bud mutation occurrence, epigenetic mechanisms mediated by DNA methylation begun only last year [46-48], and all of them were done from the perspective of local DNA methylation. Few studies were performed on global DNA methylation in bud variation with a large sample size even on fruit crops. ~~, which are an important source of new varieties of fruit trees, are still in the initial stage~~. ~~–~~In the present study, we have incorporated a large-scale collection of ‘Fuji’ mutants from around the world as plant materials. Moreover, investigations of the genetic and epigenetic perspectives, at both the individual variety

Formatted: Font: 12 pt

Formatted: Don't adjust space between Latin and Asian text, Don't adjust space between Asian text and numbers

and mutant groups were conducted for the first time. ~~In this study conducted on a large scale, excellent ‘Fuji’ varieties from around the world were collected and investigated from both the genetic and epigenetic perspectives and at both the individual~~varieties ~~and population levels for the first time.~~ Our studies revealed wide DNA methylation alterations between bud mutations in the ‘Fuji’ line. The results ~~will~~ would be helpful for comprehensively understanding the epigenetic characteristics and differentiation of the ‘Fuji’ line ~~and~~ . It also benefits for ~~provide basic information for~~ future further research on the epigenetic mechanism of bud mutations.

From an adaptive perspective, the modification of methylation status may allow trees to rapidly respond to abrupt changes in environmental conditions and contribute to their long-term responses to more general environmental scenarios [57~~[47]. Bud mutations often occur under stresses such as drought, pathogen infection, extreme weath~~er and climate conditions, limited nutrient availability, human activities, and natural or artificial selection and are a result of adaptation to abnormal external environmental conditions [~~48, 49~~58, 59]. The contribution of epigenetic modification to the ability of plants to adapt to various stresses has been well demonstrated [26~~17~~, 22~~32~~]. Therefore, it can be theoretically deduced that a certain mutation is ~~theoretically~~ associated with epigenetic variation to some extent. This study detected nearly 32% epigenetic differentiation between the mutants and their original mother ‘Fuji’ ~~and~~, showing many types of methylation pattern variants, and ~~found many types of methylation variation patterns,~~ indicating that the variation in DNA methylation might be involved in the occurrence of ‘Fuji’ mutations~~, ~~. Our results explain the differentiation of epi-phenotypes, related to the similar genome structure, highlighted by Guarino et al. [60]. This may also verify the hypothesis of significant plant promotion to environmental adaptation, by phenotype diversification, increasing the probability of the plant to thrive when faced with a changing environment [3].~~which was consistent with the theoretical inference and indicated that it was feasible to use epigenetics to study related biological phenomena. Our results could be explained as highlighted by Guarino et al. [14] that different epi-phenotypes, although related to the similar genome, are hypothesized to promote significantly environmental adaptation, because they promote phenotype diversification and increase the probability to win the “war for life” in relation to environment changes~~.

Different levels and degrees of methylation occur in higher plants to maintain normal plant development [59~~49~~]. ~~The~~ In general, the total genome DNA methylation level in different species detected by MSAP analysis is between 4.7% and 60.0% ~~[5]~~ [61]. In this ~~study~~research, the DNA methylation level detected by MSAP was approximately 36%, similar to the findings of Li ~~[50]~~ [62] in apple, ~~which was~~ in accordance with the relatively stable characteristics of DNA methylation levels in this ~~variety~~species.

CG and CHG are two main forms of DNA methylation at CCGG sites~~; t~~. Their relative proportions in the organism vary among different species, ~~tissues, environmental factors and treatment methods,~~ and the majority of available reports present results show~~ing~~ that the proportion of the former is greater than of the latter ~~[51]~~ [11]. In this study, the levels of CG and CHG methylation were found to be 22.311%

Formatted: Font: 12 pt, Font color: Gray-80%, Pattern: Clear (White)

Formatted: Font: 12 pt

Formatted: Indent: First line: 1 ch

Formatted: Indent: First line: 1 ch

and 13.60599%, respectively. The level of the former was significantly higher than that of the latter, indicating that full methylation derived was the main form of DNA methylation in the 'Fuji' line., which conformed to the results in the majority of plant species.Daccord et al. [52] found that the whole-genome DNA methylation levels of Golden Delicious apple leaves were 49% (CG) and 39% (CHG) by whole-genome DNA methylation sequencing. Xu et al. [53] analyzed the stress mechanism of apple in response to drought via specific single-base methylome analysis and found that normal 'Qinguan' leaves exhibited CG and CHG genomic methylation levels of 54% and 38%, respectively. The results of our study were quite different from those of these two previous studies, which can mostly be attributed to the differences in detection methods rather than the examined varieties. Bartels et al. [5] collected complete high-throughput sequencing methylation data for nearly 30 species, and the results generally showed higher values than those produced by another detection method.

In this study, it was found that hypermethylation and demethylation occurred simultaneously in the mutations that developed in 'Fuji', but the frequency of the variation of the latter was higher than that of the former. Since DNA demethylation is often associated with active gene expression [54-55] and transposable element triggering events [56], we will place future emphasis on the biological demethylation process, starting with specific gene activation and transposable factors, to carry out a mechanistic study of the occurrence of mutations in 'Fuji'.

Formatted: Indent: First line: 1.5 ch, Don't adjust space between Latin and Asian text, Don't adjust space between Asian text and numbers

Natural mutations in plants are usually accompanied by changes in the levels of genetic and epigenetic. AFLPs and MSAPs are the two two marker types that are mosttechniques commonly used for the detection of plant genetic and epigenetic variation [13-1413-14]. Expected heterozygosity (He), also known as gene diversity, is an important parameter for the measurement of the general genetic diversity of aamong plant groups and shows wide applicability to any system of polyploidy, self-crossing or asexual reproduction in populations [57][64]. The lower the genetic diversity, the higher the degree of the genetic homogeneity of in a population. It is generally believed known that a heterozygosity value higher than 0.5 indicates genetic diversity between population varietiessthat the population shows rich genetic diversity [65]. A value lower than 0.5 indicates that the genetic diversity is low. In this study, the average genetic diversity values of the two types of molecular markers were 0.179 186 (AFLP) and 0.263 266 (MSAP), suggesting that the genetic and epigenetic diversity levels of the mutant groups of in the 'Fuji' line were relatively low. Comparatively speaking, in the three examined mutant groups, the epigenetic expected heterozygosity of the color Color mutant group was higher than those of the other two groups (P<0.01), indicating implying its relatively high genetic and epigenetic diversity. Additionally, in combination with the results from UPGMA cluster, STRUCTURE analysis, and outlier loci detection, the Color mutant group displayed unique epigenetic characteristics, which contributed largely to the epigenetic differentiation between the three mutant groups in the 'Fuji' line. This phenomenon might be related to the degree of difficulty in bud mutation of certain traits, the breeding and selection objectives in a given period, or the degree of artificial intervention.

Formatted: Indent: First line: 1 ch

The results of this study showed that between the 'Fuji' mutant groups, the pairwise

average similarity coefficient obtained from MSAP analysis was slightly lower than that obtained from AFLP analysis, whereas for AMOVA, the opposite result was obtained. These findings reflected the differences in epigenetic variation and genetic variation between different groups, indicating that epigenetic inheritance played an important role in the differentiation of different mutant groups from the same mother. However, in general, both the genetic and epigenetic characteristics between the groups showed a very high similarity coefficient, indicating very high similarity between the three ‘Fuji’ mutant groups. The samples examined in this study came from bud mutants; therefore, the high similarity between the three ‘Fuji’ mutant groups was related to their origin from the same mutant female parent and was consistent with their budding relationships. At the same time, it implied that there was a certain internal relationship between epigenetic variation and genetic variation; that is, the epigenetic similarity between groups with high genetic similarity was also high.

Our results showed that the degree of genetic differentiation between the mutant groups was generally very low (PhiPT≈0.5). In terms of epigenetic differentiation, with the exception of the spur and early-maturation mutant groups, the differences between the color mutant groups and the spur mutant groups and between the color mutant groups and the early-maturation groups were all moderate. The epigenetic differentiation between the three mutant groups of the ‘Fuji’ line was mainly due to the obvious differentiation of the color mutant group and reflected the unique epigenetic behavior that occurred during the formation and differentiation of the color mutant group. The epigenetic differentiation between the three mutant groups of the ‘Fuji’ line was mainly due to the obvious differentiation of the color mutant group and reflected the unique epigenetic behavior that occurred during the formation and differentiation of the color mutant group. In this study, the results based on genetic and epigenetic diversity index calculations as well as UPGMA clustering, PCA and genetic structure analysis based on MSAP data all supported the above conclusions. This indicated that epigenetic variation might be related to genetic variation but is not completely dependent on classical inheritance; that is, the degree of epigenetic differentiation might be significantly different between samples with high genetic consistency.

The relationship between genetics and epigenetics has been the focus of attention since the study of epigenetics started. Regarding the relationship between genetics and epigenetics, the conclusions differ in different plants. Some studies have shown that there is no association between classical genetics and DNA methylation, indicating that the generation and maintenance of classical genetics and epigenetics are relatively independent [58-59]. However, some studies have found a significant correlation between genetic diversity and epigenetic diversity [60]. In the present study, according to Mantel’s test, genetic variation and epigenetic variation showed some correlation between three examined mutant groups as well as between individuals, indicating that epigenetic variation existed independently of genetic variation in the ‘Fuji’ line. The weak correlation between the genetics and epigenetics of each mutant group in ‘Fuji’ could be explained in terms of the conditions in which these varieties occurred. Most of the variations in fruit crops are caused by different kinds of stresses, which is thought to be an adaptation to the conditions of a poor external environment [48]. Stress is

~~thought to be an important factor contributing to epigenetic variation in plants [5]. Therefore, according to the type of variation, the occurrence of mutations is to a large extent accompanied by epigenetic variation, resulting in genetic and epigenetic differences. The low weak correlation reflected the significant epigenetic role in the formation of different mutant groups in 'Fuji' apple.~~

~~The epigenetic differences between the three mutant groups could be explained by the difficulty of selection, the breeding objectives in a given period, and the degree of artificial intervention. Early-maturation cultivars of fruit trees usually ripen and are brought to market very early, and their economic value is high. However, because of the short growth period, these fruits are generally sour and exhibit a poor taste and storage tolerance. With regard to cultivars with spur branches, they bear fruit early and are compact and short, making them suitable for dense cultivation, and they present a high economic output; however, their life span is short, and their skin is usually thick, which generally results in a poor taste. Intermediate- and late-maturation varieties exhibit a long growth period and are fully developed, meaning that they present general advantages over the first two groups in terms of internal and external quality. Therefore, although much effort has been put into the breeding of early-maturation and spur varieties, most of the best varieties came from the intermediate and late-maturation varieties. The color mutant type of the 'Fuji' line is basically intermediate- or late-maturation mutants. At the beginning of the development of 'Fuji', the greatest problem was poor coloring. Based on the breeding goal of improving appearance quality, the variation of the first generation of the 'Fuji' bud mutant line was subjected to selection. Since then, based on the bud mutants of the first generation of different color lines, multiple generations of variations have been selected, and the quality is being increasingly perfected. In this process, additional artificial selection interventions were applied; many gene loci were identified, and gene expression regulation might be altered and was recorded in the genome. Therefore, the color mutant group presented much more epigenetic variation than the other mutant groups.~~

Epigenetic variations may exhibit the phenotypic differences in different environments or growth periods and even in different tissues and plants organs~~Epigenetic variations arise frequently and easily, and plants may exhibit epigenetic differences in different environments or growth periods and even in different tissues and organs~~ ~~[5]~~[61]. However, in some species such as walnut, the global genomic DNA methylation level in different tissues and organs does not differ significantly ~~[61]~~ [65]. Leaf can provide information about epigenetic modification and adaptation in response to different environmental conditions ~~[62]~~ [60]. In ~~this~~ our study, the leaves were considered as the plant material, as such, ~~our experimental tissue was the leaf, so~~ the results and conclusions of the epigenetic investigation were limited to the analysis of DNA matrix purified from particular tissue collection~~the leaves~~. The differences between the ~~early~~Early-maturation mutant group and the ~~color~~ Color mutant group were mainly reflected in the performance of fruits. Therefore, if ~~fruits~~ fruit samples of ~~the early-maturation mutation type and color~~ these two mutant types were used as the examined material, ~~,~~ the results would not be similar to those obtained for leaves, and remain inconclusive since no similar research has previously been

Formatted: Indent: First line: 2 ch, Don't adjust space between Latin and Asian text, Don't adjust space between Asian text and numbers

Formatted: Font: (Default) MinionPro-Regular, 12 pt

Formatted: Font: 12 pt

Formatted: Indent: First line: 1 ch

conducted in apple. The tissue specificity of epigenetic modifications is also unknown.whether the results would be similar to those obtained for leaves in this study, the results remain inconclusive for now since no similar research has previously been conducted in apple, and the tissue specificity of epigenetic modifications is also unknown. Related work is underway.

Additionally, we found that the mean hypermethylation and demethylation frequencies of CHG type were both significantly higher than those of CG type, indicating that variation in CHG methylation pattern played a key role in DNA methylation pattern variation in the ‘Fuji’ lines. In plants, cytosine methylation is a context-dependent process [9]. Our finding supported it. DNA methylation status reflects the outcome of the dynamic regulation of establishment, maintenance and active-removal activities. CG and CHG methylation are catalyzed by various different enzymes and are under control by different pathways [9]. Hence, in view of our findings, the mechanism of epigenetic involvement in the occurrence of Fuji bud mutation could be further explored, focusing on the act of enzymes and detailed pathways related to CHG methylation.

In conclusion, the present study uncovered abundant changes in methylation levels and patterns between not only bud mutants and their mother ‘Fuji’ but also bud mutants, indicating that it may be possible that epigenetics mediated by DNA methylation was involved in the occurrence of the ‘Fuji’ bud mutation line. The epigenetic mechanism of the Color bud mutant group was unique, which could be the focus of further research. The correlation between epigenetic variation and genetic variation was weak, showing their independence, which provided further support for using ‘Fuji’ line as ideal sets for epigenetic studies in the future.

The main proposition of natural selection is that the fittest survives. That is, the more adaptable an individualvarieties is in a given environment, the greater his chances of survival. Variation is a prerequisite for natural selection, and there are a variety of types of variation, each of which is likely to be retained in a group. Since different groups may arise in different ecological environments, each variation will gradually change in the direction guided by natural selection [63]. The variation of the ‘Fuji’ line is assumed to be a result of both natural selection and artificial selection, and the materials that have been preserved are expected to have been generated from individualvarietiess with a strong survival ability and relatively good traits, which would then accumulate in the group, resulting in group differentiation. Selected sites (outliers) usually occupy a small proportion of the plant genome (e.g., 1.82% in *Andropogon gerardii* (AFLPs) [64], 2.45% in *Alnus glutinosa* (AFLPs) [65], and 3.46% and 10.30% in *Abies alba* and *Larix decidua*, respectively (SNPs) [66], indicating the conservation of the species genome. In this study, percentages of outlier sites of 0 and 0.915% were detected by AFLP and MSAP analysis, respectively, which is consistent with studies in other crops. Moreover, these values were relatively low, which might be related to the consistency of the genetic background among the experimental materials. The epi-outlier loci obtained by MSAP analysis reflect an important contribution of the epigenetic regulation of DNA methylation in different ‘Fuji’ mutant types.

~~In a next step, such loci could be sequenced to further explore the possible mechanism of epigenetic regulation and the variation mechanism of ‘Fuji’.~~

~~Supporting information:~~

~~**S1 Fig. Variation patterns of 15 subclasses of DNA methylation patterns.**~~

~~**(TIF)**~~

~~**S2 Fig. Summary of the changes in the cytosine methylation pattern in the ‘Fuji’ mutation line compared with the standard ‘Fuji’ cultivar.**~~

~~**(TIF)**~~

~~**S1 Table. Adaptors and primer sequences used for preamplification in AFLP and MSAP analyses.**~~

~~**(DOCX)**~~

~~**S2 Table. Summary of MSAP amplification from 23 primer combinations.**~~

~~**(DOCX)**~~

~~**S3 Table. Summary of AFLP amplification in 92 materials.**~~

~~**(DOCX)**~~

~~**S4 Table. Variation of the four major types of DNA methylation patterns (CG hypermethylation, CHG hypermethylation, CG hypomethylation, and CHG hypomethylation) in the ‘Fuji’ mutants.**~~

~~**(DOCX)**~~

Acknowledgments

We are particularly grateful to ~~Thanks to~~ Dr. Changfei Guan, Dr. Dorota Ewa Kruczyńska, and Dr. Sylwia Keller-Przybyłkowicz– for English revision of the text.

Formatted: Font: (Default) Times New Roman, 10.5 pt

Formatted: Font: 10.5 pt, Not Bold

Author Contributions

Conceived and designed the experiments: Xiaoyun Du**,** Laiqing Song. **Performed the experiments:** Xiaoyun Du**.** **Data analysis:** Xiaoyun Du, Yanbo Wang, Yan Tang. **Supervision:** Minxiao Liu, ~~Zhongwu Jiang,~~ Xueqing Liu. Funding acquisition: Zhongwu Jiang, ~~Wrote~~ Writing-~~the paper~~original draft: Xiaoyun Du. Review the paper: Lingling Zhao, Yanxia Sun, Xueyong Zhang, Daliang Liu.

Formatted: Font: Bold

Formatted: Font: Bold

Formatted: Font: Bold

Formatted: Font: Bold

References

1. Shu HR. Apple. 1999. Beijing: China Agriculture Press: 186-196. Chinese.
2. Eccleston A, DeWitt N, Gunter C, Marte B, Nath D. Epigenetics. Nature. 2007; 7143: 395.
3. Balao F, Paun O, Alonso C. Uncovering the contribution of epigenetics to plant phenotypic variation in Mediterranean ecosystems. Plant biol. 2018; 20 (Suppl.1): 38-49.
4. Vogt G. Facilitation of environmental adaptation and evolution by epigenetic phenotype variation: insights from clonal, invasive, polyploid, and domesticated animals. Environ Epigenet, 2017; 3.
5. Law JA, Jacobsen SE. Establishing, maintaining and modifying DNA methylation patterns in plants and animals. Nat Rev Genet. 2010; 11, 204-220.
6. Finnegan EJ, Peacock WJ, Dennis ES. DNA methylation, a key regulator of plant development

and other processes. Curr Opin *Genet* Dev. 2000; 10: 217-223.

7. Sahu PP, Pandey G, Sharma N, Puranik S, Muthamilarasan M, Prasad M. Epigenetic mechanisms of plant stress responses and adaptation. Plant Cell Rep. 2013; 32: 1151-1159.

8. Kapoor A, Agius F, Zhu JK. Preventing transcriptional gene silencing by active DNA demethylation. FEBS Lett. 2005; 579: 5889-5898.

9. Zhang H, Lang Z, Zhu JK. Dynamics and function of DNA methylation in plants. Nat Rev Mol Cell Biol, 2018, 19: 489-506.

10. Niederhuth CE, Bewick AJ, Ji L, Alabady MS, Kim KD, Li Q, et al. Widespread natural variation of DNA methylation within angiosperms. Genome Biol. 2016; 17: 194.

11. Niederhuth CE, Bewick AJ, Ji L, Alabady MS, Kim KD, Li Q, et al. Epigenetic mechanisms of plant stress responses and adaptation. Plant Cell Rep. 2013; 32: 1151-1159.

12. Baránek M, Čechová J, Kovacs T, Eichmeier A, Wang S, Raddová J, et al. Use of Combined MSAP and NGS Techniques to Identify Differentially Methylated Regions in Somaclones: A Case Study of Two Stable Somatic Wheat Mutants. PLoS ONE. 2016; 11(10): e0165749.

13. Chwialkowska K, Korotko U, Kosinska J, Szarejko I, Kwasniewski M. Methylation sensitive amplification polymorphism sequencing (MSAP-Seq)-a method for high-throughput analysis of differentially methylated CCGG Sites in plants with large genomes. Front Plant Sci. 2017; 8: 2056.

14. Guarino F, Cicatelli A, Brundu G, Improta G, Triassi M, Castiglione S. The use of MSAP reveals epigenetic diversity of the invasive clonal populations of *Arundo donax* L.. PLoS ONE. 2019; 14(4): e0215096.

15. Reyna-lópes GE, Simpson J, Ruiz-herrera J. Differences in DNA methylation patterns are detectable during the dimorphic transition of fungi by amplification of restriction polymorphisms. Mol Gen *Genet*. 1997; 253: 703-710.

16. McClelland M, Nelson M, Raschke E. Effect of site-specific modification on restriction endonucleases and DNA modification methyltransferases. Nucleic Acids Research. 1994; 22: 3640-3659.

17. Xiong LZ, Xu CG, Maroof MAS, Zhang Q. Patterns of cytosine methylation in an elite hybrid and its parental lines, detected by a methylation-sensitive amplification polymorphism technique. *Mol Genet* Genomics. 1999; 261: 439-446.

18. Lamo K, Bhat DJ, Kour K, Solanki SPS. Mutation studies in fruit crops: a review. Int J Curr Microbiol Appl Sci. 2017; 6 (12): 3620-3633.

19. Foster TM, Aranzana MJ. Attention sports fans! The far-reaching contributions of bud sport mutants to horticulture and plant biology. Hortic Res. 2018; 5: 44.

20. Shamel AD, Pomeroy CS. Bud mutations in horticultural crops. J Hered. 1936; 27: 487-494.

21. Okie WR. Handbook of Peach and Nectarine Varieties: Performance in the Southeastern United States and Index of Names. Springfield VA, 1998, Washington, DC.

22. Mozgova I, Mikulski P, Pecinka A, Farrona S. Epigenetic mechanisms of abiotic stress response and memory in plants. Epigenetics in Plants of Agronomic Importance: Fundamentals and Applications. 2019; 1-64.

23. Zhang CF. The investigation of Fuji apple bud mutation varieties (lines) and research of S-SAP molecular marker identified technique. Northwest A & F University; 2013. Chinese.

24. Song LQ, Zhao LL, Liu MY, Tang Y, Sun YX, Li YJ. Analysis of main features and influencing factors of 'Fuji' apple sport selection. China Fruit Vegetable. 2016; 36 (2): 54-56. Chinese.

25. Vos P, Hogers R, Bleeker M, Reijans M, Van de Lee T, Hornes M, et al. AFLP: a new technique for DNA fingerprinting. *Nucleic Acids Res.* 1995; 23: 4407-4414.
26. Schulz B, Eckstein RL, Durka W. Scoring and analysis of methylation-sensitive amplification polymorphisms for epigenetic population studies. Mol Ecol Resour. 2013; 13(4): 642-653.
27. Pérez-Figueroa A. msap: a tool for the statistical analysis of methylation-sensitive amplified polymorphism data. Mol Ecol Resour. 2013; 13 (3): 522-527.
28. Rohlf FJ. NTSYS-pc: Numerical taxonomy and multivariate analysis system. 2000; Version 2.1 Exeter Publications, New York, USA.
29. Chen CJ, Xia R, Chen H, He YH. TBtools, a Toolkit for Biologists integrating various HTS-data handling tools with a user-friendly interface. Doi: http://doi.org/10.1101/289660.
30. Jombart T, Devillard S, Dufour AB, Pontier D. Revealing cryptic spatial patterns in genetic variability by a new multivariate method. Heredity. 2008; 101: 92-103.
31. Peakall R, Smouse PE. GenAlEx 6.5: genetic analysis in Excel. Population genetic software for teaching and research-an update. Bioinformatics. 2012; 28: 2537-2539.
32. IBM Corp. Released 2013. IBM SPSS Statistics for Windows, Version 22.0. Armonk, NY: IBM Corp.
33. Pritchard JK, Wen W, Falush D. 2003. Documentation for STRUCTURE software: version 2.
34. Earl DA, VonHoldt BM. Structure Harvester: a website and program for visualizing STRUCTURE output and implementing the Evanno method. Conserv *Genet Resour*. 2012; 4 (2): 359-361.
35. Jakobsson M, Rosenberg NA. CLUMPP: a cluster matching and permutation program for dealing with label switching and multimodality in analysis of population structure. Bioinformatics. 2007; 23: 1801-1806.
36. Rosenberg NA. DISTRUCT: a program for the graphical display of population structure. Mol Ecol *Notes.* 2004; 4 (1): 137-138.
37. Foll M, Gaggiotti O. A genome-scan method to identify selected loci appropriate for both dominant and codominant markers: a Bayesian perspective. Genetics. 2008; 180 (2): 977-993.
38. Jeffreys H. Theory of probability. 1961. Oxford University Press, Oxford.
39. Kalinowski, ST. Counting alleles with rarefaction: Private alleles and hierarchical sampling designs. Conserv *Genet*. 2004; 5(4): 539-543.
40. Telias A, Wang KL, Stevenson DE, Cooney JM, Hellens RP, Allan AC. Apple skin patterning is associated with differential expression of *MYB10. BMC Plant* Biol. 2011; 11: 93.
41. Xu YT, Feng SQ, Jiao QQ, Liu CC, Zhang WW, Chen WY, et al. Comparison of MdMYB1 sequences and expression of anthocyanin biosynthetic and regulatory genes between *Malus domestica* Borkh. cultivar 'Ralls' and its blushed sport. Euphytica. 2012; 185: 157-170.
42. El-Sharkawy I, Dong L, Xu K. Transcriptome analysis of an apple (*Malus*×*domesitica*) yellow fruit somatic mutation identifies a gene network module highly associated with anthocyanin and epigenetic regulation. *J Exp Biol*. 2015; 66 (22): 7359-7376.
43. Bai S, Tuan PA, Saito T, Honda C, Hatsuyama Y, Ito A, et al. Epigenetic regulation of *MdMYB1* is associated with paper bagging-induced red pigmentation of apples. Planta. 2016; 244 (3): 573-586.
44. Daccord N, Celton JM, Linsmith G, Becker C, Choisne N, Schijlen E, et al. High-quality de novo assembly of the apple genome and methylome dynamics of early fruit development. Nat Gent. 2017; 49: 1099-1108.

45. Xu J, Zhou S, Gong X, Song Y, Van Nocker S, Ma F, et al. Single-base methylome analysis reveals dynamic epigenomic differences associated with water deficit in apple. *Plant Biotechnol* J. 2018; 16: 672-687.
46. Jiang SH, Sun QG, Chen M, Wang N, Xu HF, Fang HC, et al. Methylome and transcriptome analyses of apple fruit somatic mutations reveal the difference of red phenotype. BMC Genomics. 2019; 20:117.
47. Zhang L, Hu J, Han XL, Li JJ, Gao Y, Richards CM, et al. A high-quality apple genome assembly reveals the association of a retrotransposon and red fruit colour. Nat Commun. 2019; 10: 1494.
48. Jiang SH, Wang N, Chen M, Zhang R, Sun QG, Xu HF, et al. Methylation of *MdMYB1* locus mediated by RdDM pathway regulates anthocyanin biosynthesis in apple. *Plant* Biotechnol J. 2020; 1-13.
49. Cho HJ, Kim GH, Choi C. Differential gene expression and epigenetic analyses between striped and blushed skinned sports of 'Fuji' apple. Sci Hortic-amsterdam, 2020; 261: 108944.
50. Xu J, Xu H, Liu Y, Wang X, Xu Q, Deng X. Genome-wide identification of sweet orange (Citrus sinensis) histone modification gene families and their expression analysis during the fruit development and fruit-blue mold infection process. Front Plant Sci. 2015; 6: 607.
51. Huang H, Liu R, Niu Q, Tang K, Zhang B, Zhang H, et al. Global increase in DNA methylation during orange fruit development and ripening. P Natl Acad Sci USA. 2019; 116: 1430-1436.
52. Noyer J L, Causse S, Tomekpe K, Bouet A, Baurens F C. A new image of plantain diversity assessed by SSR, AFLP and MSAP markers. Genetica. 2005; 124: 61-69.
53. Wang ZG, Meng D, Wang AD, Li TL, Jiang SL, Cong PH, et al. The methylation of *PcMYB10* promoter is associated with Green skinned sport in 'Max Red Bartlett' pear. Plant Physiol, 2013, 162: 885-896.
54. Qian M, Sun Y, Allan AC, Teng Y, Zhang D. The red sport of 'Zaosu' pear and its red-striped pigmentation pattern are associated with demethylation of the *PyMYB10* promoter. Phytochemistry. 2014; 107: 16-23.
55. Cheng JF, Niu QF, Zhang BF, Chen KS, Yang RH, Zhu JK, et al. Downregulation of RdDM during strawberry fruit ripening. Genome Biol. 2018; 19:212.
56. Guo DL, YuYH, Xi FF, Shi YY, Zhang GH. Histological and molecular characterization of grape early ripening bud mutant. Int J *Genomics.* 2016; 4: 1-7.
57. Schellenbaum P, Mohler V, Wenzel G, Walter B. Variation in DNA methylation patterns of grapevine somaclones (*Vitis vinifera* L.). BMC Plant Biol. 2008; 8(1): 78.
58. Sáez-Laguna E, Guevara MÁ, Díaz LM, Sánchez-Gómez D, Collada C, Aranda I, et al. Epigenetic variability in the genetically uniform forest tree species *Pinus pinea* L. PLoS One. 2014; 9(8): e103145.
59. Jing Shixi works collection Editorial Board. Jing Shixi works collection. 2009; Liaoning University Press: p24-40.
60. Whalley K. Dynamic DNA methylation. Nat Rev Neurosci. 2007; 8: 323.
61. Guarino F, Cicatelli A, Brundu G, Heinze B, Castiglione S. Epigenetic diversity of clonal white Poplar (*Populus alba* L.) populations: could methylation support the success of vegetative reproduction strategy? PLoS ONE. 2015; 10:e0131480.
62. Bartels A, Han Q, Nair P, Stacey L, Gaynier H, Mosley M, et al. Dynamic DNA methylation in plant growth and development. Int J Mol Sci. 2018; 19: 2144.
63. Li LG. Enhancement and identification of ploidy germplasms in apple. Shenyang Agricultural

University; 2008. Chinese.

64. Nei M. Analysis of gene diversity in subdivided populations. Proc Natl Acad Sci USA. 1973; 70: 3321-3323.

65. Sheriff O, Alemayehu K. Genetic diversity studies using microsatellite markers and their contribution in supporting sustainable sheep breeding programs: A review. Cogent Food and Agriculture. 2018; 4: 1459062.

66. Zhou BB. Modification of DNA methylation by grafting to impact on phenotype of walnut. Chinese Academy of Forestry; 2014. Chinese.

Supporting information:

S1 Fig. Variation patterns of 15 subclasses of DNA methylation patterns.

(TIF)

S2 Fig. Summary of the changes in the cytosine methylation pattern in the 'Fuji' mutation line compared with the standard 'Fuji' cultivar.

(TIF)

S1 Table. Adaptors and primer sequences used for preamplification in AFLP and MSAP analyses.

(DOCX)

S2 Table. Summary of MSAP amplification from 23 primer combinations.

(DOCX)

S3 Table. Summary of AFLP amplification in 92 materials.

(DOCX)

S4 Table. Variation of the four major types of DNA methylation patterns (CG hypermethylation, CHG hypermethylation, CG hypomethylation, and CHG hypomethylation) in the 'Fuji' mutants.

(DOCX)

S5 Table. Number and frequency of change in cytosine methylation pattern in the bud sports compared with the standard 'Fuji'.

(XLSX)

S6 Table. Genetic similarity based on MSAP-MSL profiles.

(XLSX)

S7 Table. Genetic similarity based on AFLP data profiles.

(XLSX)

1. Eccleston A, DeWitt N, Gunter C, Marte B, Nath D. Epigenetics. Nature. 2007; 7143: 395.

2. Law JA, Jacobsen SE. Establishing, maintaining and modifying DNA methylation patterns in plants and animals. Nat Rev Genet. 2010; 11, 204-220.

3. Finnegan EJ, Peacock WJ, Dennis ES. DNA methylation, a key regulator of plant development and other processes. Curr Opin Genet Dev. 2000; 10: 217-223.

4. Sahu PP, Pandey G, Sharma N, Puranik S, Muthamilarasan M, Prasad M. Epigenetic mechanisms of plant stress responses and adaptation. Plant Cell Rep. 2013; 32: 1151-1159.

5. Bartels A, Han Q, Nair P, Stacey L, Gaynier H, Mosley M, et al. Dynamic DNA methylation in plant growth and development. Int J Mol Sci. 2018; 19: 2144.

6. Sudan J, Raina M, Singh R. Plant epigenetic mechanisms: role in abiotic stress and their generational heritability. 3 Biotech. 2018; 8: 172.
7. Dyachenko OV, Zakharchenko S, Shevchuk TV, Bohnert H J, Cushman JC, Buryanov YI. Effect of hypermethylation of CCWGG sequences in DNA of *Mesembryanthemum crystallinum* plants on their adaptation to salt stress. Biorak. 2006; 71: 461-465.
8. Zhao YL, Yu X, Ye WW, Wang HM, Wang JJ, Fang BX. Study on DNA cytosine methylation of cotton (*Gossypium hirsutum* L.) genome and its implication for salt tolerance. Agr Sci China. 2010; 9: 783-791.
9. Lewis NL, Zhan S. The plant genome's methylation status and response to stress: implications for plant improvement. Curr Opin Plant Biol. 2007; 10: 317-322.
10. Guo WL, Hussain N, Wu R, Liu B. High hypomethylation and epigenetic variation in fragmented populations of wild barley (*Hordeum Brevisubulatum*). Pakistan J Bot. 2018; 50 (4): 1379-1386.
11. Kapoor A, Agius F, Zhu JK. Preventing transcriptional gene silencing by active DNA demethylation. FEBS Lett. 2005; 579: 5889-5898.
12. Ng HH, Bird A. DNA methylation and chromatin modification. Curr Opin Genet Dev. 1999; 9:158-163.
13. Chwialkowska K, Korotko U, Kosinska J, Szarejko I, Kwasniewski M. Methylation sensitive amplification polymorphism sequencing (MSAP-Seq)-a method for high-throughput analysis of differentially methylated CCGG Sites in plants with large genomes. Front Plant Sci. 2017; 8: 2056.
14. Guarino F, Cicatelli A, Brundu G, Improta G, Triassi M, Castiglione S. The use of MSAP reveals epigenetic diversity of the invasive clonal populations of *Arundo donax* L.. PLoS ONE. 2019; 14(4): e0215096.
15. Reyna-lópes GE, Simpson J, Ruiz-herrera J. Differences in DNA methylation patterns are detectable during the dimorphic transition of fungi by amplification of restriction polymorphisms. Mol Gen Genet. 1997; 253: 703-710.
16. Xiong LZ, Xu CG, Maroof MAS, Zhang Q. Patterns of cytosine methylation in an elite hybrid and its parental lines, detected by a methylation-sensitive amplification polymorphism technique. Mol Genet Genomics. 1999; 261: 439-446.
17. Schulz B, Eckstein RL, Durka W. Scoring and analysis of methylation-sensitive amplification polymorphisms for epigenetic population studies. Mol Ecol Resour. 2013; 13(4): 642-653.
18. Cervera MT, Ruiz-Garcia L, Martinez-Zapater JM. Analysis of DNA methylation in Arabidopsis thaliana based on methylation-sensitive AFLP markers. Mol Genet Genomics. 2002; 268: 543-552.
19. Lamo K, Bhat DJ, Kour K, Solanki SPS. Mutation studies in fruit crops: a review. Int J Curr Microbiol Appl Sci. 2017; 6 (12): 3620-3633.
20. Foster TM, Aranzana MJ. Attention sports fans! The far-reaching contributions of bud sport mutants to horticulture and plant biology. Hortic Res. 2018; 5: 44.
21. Shu HR. Apple. 1999. Beijing: China Agriculture Press: 186-196.
23. Song LQ, Zhao LL, Liu MY, Tang Y, Sun YX, Li YJ. Analysis of main features and influencing factors of 'Fuji' apple sport selection. China Fruit Vegetable. 2016; 36 (2): 54-56. Chinese.
23. Zhang CF. The investigation of Fuji apple bud mutation varieties (lines) and research of S-SAP molecular marker identified technique. Northwest A & F University; 2013. Chinese.

24. Xu YT, Feng SQ, Jiao QQ, Liu CC, Zhang WW, Chen WY, et al. Comparison of MdMYB1 sequences and expression of anthocyanin biosynthetic and regulatory genes between *Malus domestica* Borkh. cultivar 'Ralls' and its blushed sport. Euphytica. 2012; 185: 157-170.
25. Zhang L, Hu J, Han XL, Li JJ, Gao Y, Richards CM, et al. A high-quality apple genome assembly reveals the association of a retrotransposon and red fruit colour. Nat Commun. 2019; 10: 1494.
26. Telias A, Wang KL, Stevenson DE, Cooney JM, Hellens RP, Allan AC. Apple skin patterning is associated with differential expression of *MYB10*. BMC Plant Biol. 2011; 11: 93.
27. Qian M, Sun Y, Allan AC, Teng Y, Zhang D. The red sport of 'Zaosu' pear and its red-striped pigmentation pattern are associated with demethylation of the PyMYB10 promoter. Phytochemistry. 2014; 107: 16-23.
28. El-Sharkawy I, Dong L, Xu K. Transcriptome analysis of an apple (*Malus×domesitica*) yellow fruit somatic mutation identifies a gene network module highly associated with anthocyanin and epigenetic regulation. J Exp Biol. 2015; 66 (22): 7359-7376.
29. Bai S, Tuan PA, Saito T, Honda C, Hatsuyama Y, Ito A, et al. Epigenetic regulation of *MdMYB1* is associated with paper bagging-induced red pigmentation of apples. Planta. 2016; 244 (3): 573-586.
30. Guo DL, YuYH, Xi FF, Shi YY, Zhang GH. Histological and molecular characterization of grape early ripening bud mutant. Int J Genomics. 2016; 4: 1-7.
31. Schellenbaum P, Mohler V, Wenzel G, Walter B. Variation in DNA methylation patterns of grapevine somaclones (*Vitis vinifera* L.). BMC Plant Biol. 2008; 8(1):78.
32. Mozgova I, Mikulski P, Pecinka A, Farrona S. Epigenetic mechanisms of abiotic stress response and memory in plants. Epigenetics in Plants of Agronomic Importance: Fundamentals and Applications. 2019; 1-64.
33. Vos P, Hogers R, Bleeker M, Reijans M, Van de Lee T, Hornes M, et al. AFLP: a new technique for DNA fingerprinting. Nucleic Acids Res. 1995; 23: 4407-4414.
34. Li SY, Xia Q, Wang F, Yu XM, Ma J, Kou HP, et al. Laser irradiation-induced DNA methylation changes are heritable and accompanied with transpositional activation of mPing in rice. Front Plant Sci. 2017; 8: 363.
35. Rohlf FJ. NTSYS-pc: Numerical taxonomy and multivariate analysis system. 2000; Version 2.1 Exeter Publications, New York, USA.
36. Pérez-Figueroa A. msap: a tool for the statistical analysis of methylation-sensitive amplified polymorphism data. Mol Ecol Resour. 2013; 13 (3): 522-527.
37. Jombart T, Devillard S, Dufour AB, Pontier D. Revealing cryptic spatial patterns in genetic variability by a new multivariate method. Heredity. 2008; 101: 92-103.
38. Peakall R, Smouse PE. GenAlEx 6.5: genetic analysis in Excel. Population genetic software for teaching and research-an update. Bioinformatics. 2012; 28: 2537-2539.
39. IBM Corp. Released 2013. IBM SPSS Statistics for Windows, Version 22.0. Armonk, NY: IBM Corp.
40. Pritchard JK, Wen W, Falush D. 2003. Documentation for STRUCTURE softward: version 2.
41. Earl DA, VonHoldt BM. Structure Harvester: a website and program for visualizing STRUCTURE output and implementing the Evanno method. Conserv Genet Resour. 2012; 4 (2): 359-361.
42. Jakobsson M, Rosenberg NA. CLUMPP: a cluster matching and permutation program for dealing with label switching and multimodality in analysis of population structure.

Bioinformatics. 2007; 23: 1801-1806.

43. Rosenberg NA. DISTRUCT: a program for the graphical display of population structure. Mol Ecol Notes. 2004; 4 (1): 137-138.

44. Foll M, Gaggiotti O. A genome-scan method to identify selected loci appropriate for both dominant and codominant markers: a Bayesian perspective. Genetics. 2008; 180 (2): 977-993.

45. Jeffreys H. Theory of probability. 1961. Oxford University Press, Oxford.

46. Kalinowski, ST. Counting alleles with rarefaction: Private alleles and hierarchical sampling designs. Conserv Genet. 2004; 5(4): 539-543.

47. Sáez-Laguna E, Guevara MÁ, Díaz LM, Sánchez-Gómez D, Collada C, Aranda I, et al. Epigenetic variability in the genetically uniform forest tree species *Pinus pinea* L. PLoS One. 2014; 9(8): e103145.

48. Jing Shixi works collection Editorial Board. Jing Shixi works collection. 2009; Liaoning University Press: p24-40.

49. Whalley K. Dynamic DNA methylation. Nat Rev Neurosci. 2007; 8: 323.

50. Li LG. Enhancement and identification of ploidy germplasms in apple. Shenyang Agricultural University; 2008. Chinese.

51. Niederhuth CE, Bewick AJ, Ji L, Alabady MS, Kim KD, Li Q, et al. Epigenetic mechanisms of plant stress responses and adaptation. Plant Cell Rep. 2013; 32: 1151-1159.

52. Daccord N, Celton JM, Linsmith G, Becker C, Choisne N, Schijlen E, et al. High-quality de novo assembly of the apple genome and methylome dynamics of early fruit development. Nat Gent. 2017; 49: 1099-1108.

53. Xu J, Zhou S, Gong X, Song Y, Van Nocker S, Ma F, et al. Single-base methylome analysis reveals dynamic epigenomic differences associated with water deficit in apple. Plant Biotechnol J. 2018; 16: 672-687.

54. Steward N, Ito M, Yamaguchi Y, Koizumi N, Sano H. Periodic DNA methylation in maize nucleosomes and demethylation by environmental stress. J Biol Chem. 2002; 277: 37741-37746.

55. Bharti P, Mahajan M, Vishwakarma AK, Bhardwaj J, Yadav SK. *AtROS1* overexpression provides evidence for epigenetic regulation of genes encoding enzymes of flavonoid biosynthesis and antioxidant pathways during salt stress in transgenic tobacco. J Exp Bot. 2015; 66: 5959-5969.

56. Saze H, Tsugane K, Kanno T, Nishimura T. DNA methylation in plants：relationship to small RNAs and histone modifications, and functions in transposon inactivation. Plant Cell Physio. 2012; 53: 766-84.

57. Nei M. Analysis of gene diversity in subdivided populations. Proc Natl Acad Sci USA. 1973; 70: 3321-3323.

58. Ashikawa I. Surveying CpG methylation at 5'-CCGG in the genomes of rice cultivars. Plant Mol Biol. 2001; 45:31-39.

59. Vaughn MW, Tanurdzic M, Lippman Z, Jiang H, Carrasquillo R, Rabinowicz PD, et al. Epigenetic natural variation in *Arabidopsis thaliana*. PLos Biol. 2007; 5: 1617-1629.

60. Keyte AL, Ryan P, Liu B, Wendel JF. Infraspecific DNA methylation polymorphism in cotton (*Gossypium hirsutum* L.). J Hered. 2006; 97 (5): 444-450.

61. Zhou BB. Modification of DNA methylation by grafting to impact on phenotype of walnut. Chinese Academy of Forestry; 2014. Chinese.

62. Guarino F, Cicatelli A, Brundu G, Heinze B, Castiglione S. Epigenetic diversity of clonal white

Poplar (*Populus alba* L.) populations: could methylation support the success of vegetative reproduction strategy? PLoS ONE, 2015; 10:e0131480.

63. Futuyma DJ. Evolutionary Biology. 1986. Sinauer Associates, Sunderland, MA.
64. Gray MM, Amand PS, Bello NM, Galliart MB, Knapp M, Garrett KA, et al. Ecotypes of an ecologically dominant prairie grass (*Andropogon gerardii*) exhibit genetic divergence across the U.S. Midwest grasslands' environmental gradient. Mol Ecol. 2014; 23: 6011-6028.
65. Cox K, Vanden BA, Van C, Mergeay J. Temperature- related natural selection in a wind-pollinated tree across regional and continental scales. Mol Ecol. 2011; 20: 2724-2738.
66. Mosca E, González-Martínez SC, Neale DB. Environmental versus geographical determinants of genetic structure in two subalpine conifers. New Phytol. 2014; 201: 180-192.

Dear editor:

Thank you for the thoughtful and thorough review. We are very grateful for your and reviewer's nice comments on our manuscript. According to the advices, we amended the relevant parts in manuscript (No. PONE-D-20-01600).

The editorial decision on our previous manuscript was major revision. Moreover, some problems in language were existed. So, in general, the present manuscript has been greatly revised in contents as well as language. Some problems in grammar raised by reviewers were corrected and rephrased; some were removed due to the adjustment in contents. Please see the new revision for details. The followings are mainly answers to the questions raised by reviewers. Thank you very much!

To #1 reviewer:

Comment	Response
■ -Text is wordy and language not well structured. English should be improved throughout.	The section of the Introduction and Discussion have been rewritten. Also, we asked AJE: English Editing & Author Services for Research Publication as well as our friends oversea to help do correction in the revised manuscript. The certificate from AJE company will be attached in this manuscript. We are really sorry for the troubles caused to you because of our poor English.
■ line 91-94. Not clear. Score '1' we also get by digestion by either HpaII, MspI enzymes due to no methylation (band present in both).	This is done in order to distinguish between MSLs (methylation-susceptible) and NMLs (nonmethylated) data in the MSAP amplification results since only the MSP data are meaningful. We use the R package *msap* for relevant statistics, not manual score. Therefore, in the revised manuscript, avoiding wordy, the explanation of this part was removed in the introduction section. Actually, the '1' here refers to two situations that a certain sample is cut by *Hpa*II and *Msp*I. For situation of H0M1 or H0M1, when the band is scored to make MSLs matrix, both will be recorded '1'.
■Is not clear if Fuji mutants are considered varieties or genotypes.	Fuji mutants are considered varieties. We have unified all varieties in the new version manuscript.
■It is not clear how many individual plants were used from each of the 91 elite varieties	We used pooled individuals (ca. 5 plants being clonally propagated from a single mother variety) of each variety for analysis. (see line 141 in the new version manuscript)
■It is not clear how many technical repeats were used in the AFLP and MSAP experiments	The repeatability of banding patterns assessed by conducting two sets of independent MSAP and AFLP analyses and only the consistent bands were included.

	(see line 159-160 in the new version manuscript)
■line 157. How were leaves stored?	Young leaves immediately frozen in liquid nitrogen and then stored at -80℃ prior to DNA isolation. (see line 142 in the new version manuscript)
■lines 157-158. Which were these developmental and phenological stages? Perhaps authors could be more specific.	We sampled plant materials with the same age on the same dates (12 June 2016) when they were bearing fully expanded leaves. (see line 138 in the new version manuscript)

To #2 reviewer:

Content	Comment	Response
Introduction	■it seems that more than one author wrote the introduction. English must be improved in several parts (especially the first half).	The section of the Introduction and Discussion have been rewritten. Also, we asked AJE: English Editing & Author Services for Research Publication as well as our friends oversea to help do correction in the revised manuscript. The certificate from AJE company will be attached in this manuscript. We are really sorry for the troubles caused to you because of our poor English.
	■line85. more details about these isozymes	Done. (See line 86 in the new version manuscript)
Materials and methods	■Several small corrections are included in the attached file.	All done in the new version manuscript.
	■line156. How many samples per variety?	We used pooled individuals (ca. 5 plants being clonally propagated from a single mother variety) of each variety for analysis (see line 141 in the new version manuscript). One sample was used per variety in this study.
	■line171 and 177. Its better to be presented in a table as a supplementary data	Done.
	■line201. this table goes to results section	Done.
Results	■ Statistics must be clearly improved. It should be clear to the readers which results are statistically significant and which are not.	In the new revised version, we have tried our best doing complementary in statistically significant analysis for items that could be conducted for significant analysis. But we are really sorry.

		Because we used one mixed sample per variety in our study, for some items, like CVs between different mutant groups, it was impossible to perform significance analysis.
	■line278. what statistical analysis have you conducted to say that??	In the new revised draft, we performed a statistical analysis of the significant difference between CG and CHG methylation levels.
	■line291. In how many cases do they occur these changes in the patterns of cytosine methylation?	We have tabulated the statistical results in the supplementary materials section. (See new added S5 Table)
	■line303. 91 varieties???	Yes. It should be 91 varieties.
	■line305. this paragraph better fits to the discussion section.<br>line313. also this paragraph better fits to the discussion section.	The relevant content in these two paragraphs has been simplified and doing statement in the discussion section.
	Line447. not a clear seperation between the different groups	Yes. Because this is the clustering result based on AFLP data. The results showed that, unlike the epigenetic results, there was not a clear separation between the different groups genetically. They just presented local clustering and mixed phenomena.
	■Line452. tested<br>you have to be consistent using either genotypes, samples or varieties	Thank for your advice very much. We have changed all of them to variety as a unified statement.
Discussion	The discussion should be more focused on results. It's not clear If the epigenetic differences are significantly different between the three groups.	We adjusted the content in the discussion section to make our discussion more focus on results. Moreover, we did difference significance analysis of epigenetic differences between the three groups. The result was significantly different between the three groups.
	The discussion is very big and some points are very assumptive. it should be shortened and provide a clear conclusion which correlates directly with the results.	According to your advice, discussion was shorted. Additionally, we also added conclusions in the discussion section.
	■line667. Can you provide any literature?	Done. Please see literature 39-56.
	■line716. is it statistically higher? please verify	Yes. We performed a statistical analysis of the significant difference between CG and CHG methylation levels. The result exhibited significant difference ($P<0.01$).
	■line720-729. Is there any literature supporting these results too?	Because of the adjustment in content, this part of the analysis was removed in the new version manuscript.

	■line733. moderate? what does this mean?	Because of the adjustment in content, this part of the analysis was removed in the new version manuscript.
	■line776. not very convincing explanation	Because of the adjustment in content, this part of the analysis was removed in the new version manuscript.
	■line792. please rephrase	This part of the content seemed not directly related to our results. So the analysis of this part was removed in the new version manuscript.
	■line806. More future perspectives? which are briefly, the financial advantages of a further research of epigenetic regulation mechanisms?	In view of our findings, we will focus on the study on the act of enzymes and detailed pathway related to the CHG methylation in order to explore the much deeper mechanism of epigenetic involvement in the occurrence of Fuji bud mutation.
