# Supplementary material for: Mapping QTLs underpin nutrition components in aromatic rice germplasm
Source: PLoS One. 2020 Jun 11;15(6):e0234395. doi: 10.1371/journal.pone.0234395 (PMC7289389; doi:10.1371/journal.pone.0234395)
Supplement: Attachment — Submitted filename: Response to Reviewers comments.docx [file pone.0234395.s004.docx]

PONE-D-19-31392
Nutritional associated QTLs and diversity analysis in aromatic rice germplasm

**Comment:** We suggest you thoroughly copyedit your manuscript for language usage, spelling, and grammar. If you do not know anyone who can help you do this, you may wish to consider employing a professional scientific editing service.

**Response:** Prof Barry Pittendrigh, Michigan State University, USA edited the paper.

**Additional Editor Comments (if provided):**

**Comment:** The manuscript entitled: “Nutritional associated QTLs and diversity analysis in aromatic rice germplasm” presents new QTL for aromatic rice varieties in relation with Fe and Zn concentrations. The manuscript, first of all, needs English editing and I also have some major comments below.

**Response:** Thank you so much. The manuscript has been edited by a native English expert Prof Barry Pttendrigh, potential coauthor

**Comment:** Abstract: needs rewriting and English editing

**Response:** It has been revised and edited.

**Comment:** How cluster analysis has been performed? It must be written in the abstract also. How was the QTL were produced?

**Response:** The cluster analysis and QTL identification procedures are written in the materials and methods section and also briefly added in the abstract. Line 351-361, Line 340-342.

**Comment:** Line 80: write LSD as Least Square Distance

**Response:** Thanks, we elaborated the full meaning of LSD that indicate in our paper. LSD indicates Least Significant Difference. This full name has been added in the text.

**Comment:** Line 222: here you report that 45 polymorphic markers were distributed across the 12 chromosomes, how do you know that? Did you perform the analysis or you got this information from previously published papers? I suppose that you want to say that you elected these SSR from the database in order that SSR will be distributed across the 12 chromosomes …etc

**Response:** Yes, you are right. We get these SSR information from Gramene website (<https://archive.gramene.org/markers/microsat/>). This can be found in the reference number 44.

**Comment:** Line 257: probability must be indicated with small letter

**Response:** Okay, thank you. The P is corrected with p in the text.

**Comment:** Line 95: while you discuss about results of cluster analysis, there is no description on materials and methods of the algorithm used for the construction of clusters

**Response:** Thank you very much for your nice comment. Now we added the method which was used to construct cluster. A UPGMA cluster dendrogram of 113 aromatic rice germplasm based on 4 physio-chemical traits was constructed using PAST software package (46) following Ward's hierarchical clustering method. Line 340-342.

**Comment:** Line 137: why this variety had highest concentration, discuss more about this issue.

**Response:** Thank you very much this comment. We discussed this issue elaborately in discussion section. Line 170-177.

**Comment**: Line 143: did you have any common genotypes or varieties with other studies?

**Response:** Yes. We used common germplasm which was already used in other studies both phenotypic and genotypic analysis (Islam et al. 2017). In this study, we used four quantitative physico-chemical data (Fe, Zn, protein and amylose content) only which were not published anywhere.

Islam MZ, M Khalequzzaman, M K Bashar, N A Ivy, MA K Mian, BR Pittendrigh, MM Haque and **MP Ali** (2018) Variability assessment of aromatic rice germplasm by pheno-genomic traits and population structure analysis. Scientific Reports volume 8, Article number: 9911.

**Comment:** Line 155: what is GLM analysis?

**Response:** GLM stands for General linear model. This model was used for assessment of marker-trait associations (MTA). Line 353.

**Comment**: Line 157: what is GY?

**Response**: GY stands grain yield. Corrected in the text.

**Comment**: Line 158: what is IRRI?

**Response:** IRRI stands for International Rice Research Institute. Now added in text.

**Comment:** I cannot understand from the manuscript quite a lot points. Did you perform association mapping and after CIM for QTL identification? please provide details about the method. This is not clearly described in the materials and methods.

**Response:** We performed association mapping for four quantitative traits viz., Fe, Zn, amylose and protein content in rice grain. We also performed CIM for QTLs identification and map was constructed using MAPdisto version 2.0 [51]. Line 352-361.

**Comment:** A LOD equal to 2.02 is not a strong LOD in order to state that you found a highly significant QTL, Also you do not discuss about the variance explained from the QTL which is also low (7.89%), provide examples from other studies with LOD scores and variance explained.

**Response:** Thank for this important comment which strongly improve our manuscript. We added this information in discussion section. Lines 222-244.

**Comment:** Also you do not discuss about positions of QTL; this is only written in the abstract.

**Response:** Thanks for this comment. Now we have added the position of all QTLs in the discussion part. Line 143-150. Line 218-234.
